# Radiolytic degradation of dodecane substituted with common energetic functional groups†

**DOI:** 10.1039/d3ra00998j

**Published:** 2023-03-21

**Authors:** Patricia L. Huestis, Nicholas Lease, Chris E. Freye, Daniel L. Huber, Geoffrey W. Brown, Daniel L. McDonald, Tammie Nelson, Christopher J. Snyder, Virginia W. Manner

**Affiliations:** a Los Alamos National Laboratory USA vwmanner@lanl.gov

## Abstract

Explosives exist in and are expected to withstand a variety of harsh environments up to and including ionizing radiation, though little is known about the chemical consequences of exposing explosives to an ionizing radiation field. This study focused on the radiation-induced chemical changes to a variety of common energetic functional groups by utilizing a consistent molecular backbone. Dodecane was substituted with azide, nitro, nitrate ester, and nitramine functional groups and γ-irradiated with ^60^Co in order to study how the functional group degraded along with what the relative stability to ionizing radiation was. Chemical changes were assessed using a combination of analysis techniques including: nuclear magnetic resonance (NMR) spectroscopy, gas chromatography of both the condensed and gas phases, Raman spectroscopy, and Fourier transform infrared (FTIR) spectroscopy. Results revealed that much of the damage to the molecules was on the energetic functional group and often concentrated on the trigger linkage, also known as the weakest bond in the molecule. The general trend from most to least susceptible to radiolytic damage was found to be D–ONO_2_ → D–N_3_ → D–NHNO_2_ → D–NO_2_. These results also appear to be in line with the relative stability of these functional groups to things such as photolysis, thermolysis, and explosive insults.

## Abstract 2

The relative radiolytic stability of dodecane functionalized with common energetic functional groups was explored with gamma irradiation and probed by various analytical techniques.

## Graphical abstract

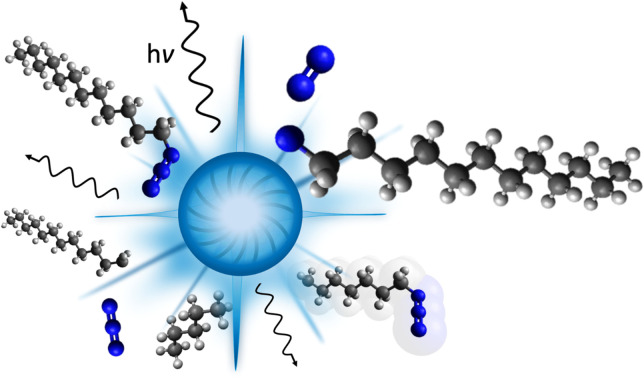

## 1. Introduction

Explosives find use in many applications such as military,^1^ mining,^2^ medicine,^3–5^ and even space exploration.^6–8^ The variety of applications require explosives to be exposed to a large range of harsh conditions, one of which is ionizing radiation. Very little is known about how explosives are affected by ionizing radiation fields, and the logistical challenges associated with irradiating high explosives leads to little progress on that front. Additionally, the large number of explosives that are currently in use make it impractical to study the effects of radiation on every known explosive. Instead, a more generalized approach should be used to make broad predictions on the radiolytic stability for both currently used and not yet discovered explosives.

Ionizing radiation is able to drive chemistry far from equilibrium through the creation of radicals and highly excited states. These reactive species lead to complicated chemistry as they react with the surrounding system, and the resultant products are not often easy to predict. Different structures, for instance, can display different tolerances to ionizing radiation even when the same atoms are present.^9^ Because explosives tend to utilize different backbone structures in addition to different energetic functional groups (FGs), it can be difficult to differentiate between backbone and FG resistance to ionizing radiation when comparing across multiple explosives. This study aims to understand the relative stability of commonly used energetic FGs themselves, so a common molecular backbone was used: dodecane.

**Fig. 1:**
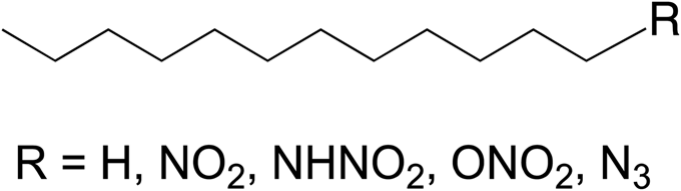
Generalized molecular structures of the materials utilized in this study.

Though many backbones could have in theory been used for this study, dodecane was a reasonable first choice due to the fact that it is commonly used in nuclear reprocessing^10,11^ and thus its stability to ionizing radiation has been studied.^11–13^ In addition, it is a low volatility liquid at ambient temperature and pressure and relatively cheap and easy to work with which make it a good candidate as the common backbone molecule for this study. Although several of the substituted dodecane molecules used in this study have been synthesized before, none were commercially available and thus were synthesized in-house. The energetic FGs chosen for this study were: azide (–N_3_), nitro (–NO_2_), nitrate ester (–ONO_2_), and nitramine (–NHNO_2_); the generalized molecular structure for these materials can be found in Fig. 1. These same molecules were also the focus of an earlier photolytic computational study.^14^ Although photolysis and radiolysis are different processes, it is still interesting to make broad comparisons across the different energy regimes.

In this paper, the various substituted dodecanes were synthesized, irradiated with ^60^Co under vacuum, and then analyzed post radiolysis in order to compare with the unirradiated (control) material. Unsubstituted dodecane was also irradiated and analyzed in a similar manner to provide a benchmark system for radiation effects of the backbone structure. Analysis was completed using a suite of techniques. Nuclear magnetic resonance (NMR) spectroscopy, gas chromatography-time of flight mass spectrometry (GC-TOFMS), Raman spectroscopy, and Fourier transform infrared (FTIR) spectroscopy were used to analyze the condensed phase for differences resulting from irradiation. Radiolytically-produced gas was analyzed using gas chromatography-mass spectrometry (GC-MS) and the total gas produced during irradiation was used as a general benchmark for radiolytic activity.

In this manuscript, all details related to the synthesis, sample preparation and irradiation, and analysis are discussed in detail, including the synthesis of a compound used for proper identification of a suspected radiolytic degradation product that was not able to be purchased. The most important degradation products are identified and organized by compound. The results are then benchmarked against previous studies on the irradiation of alkanes and relevant explosives along with photolysis, thermolysis, and small-scale sensitivity measurements and computations in order to make broader statements about explosive degradation. Notably, this study is the first of its kind to investigate radiolytic degradation of targeted energetic FGs by combining the disparate fields of energetic material synthesis, trace analytical techniques, and high-energy radiation.

## 2. Methods and materials

Laboratory grade dodecane (D–H, D = C_12_H_25_) was purchased from Fisher Chemical and used as is. Analytical standard grade chemicals used for GC-TOFMS identification of octanol, octanal, decanal, dodecanol, and dodecene were purchased from Sigma Aldrich and used as is. All other compounds were synthesized in-house and had a purity >97% as measured with ^13^C- and ^1^H-NMR spectroscopy.

### 2.1 Synthesis of dodecane-based compounds

Nitric acid (fuming), triflouroacetic anhydride were purchased from Acros chemical and used as received. Dodecanol, dodecylamine and 1-bromododecane were all purchased from Sigma Aldrich (reagent grade) and used as received. Deuterated solvents (chloroform d) were purchased from Cambridge Isotope Laboratories and used as received. ^1^H-NMR and ^13^C-NMR spectra were recorded on a 400 MHz Bruker spectrometer. NMR signals were referenced to residual solvent signals in the deuterated solvents. Synthesis reaction schemes can be found in Fig. S2 in the ESI.†

#### 2.1.1 1-Azidododecane (D–N_3_)

1-Azidododecane was synthesized using 1-bromododecane and sodium azide in DMF following known literature procedures.^15^^1^H-NMR (400 MHz, CDCl_3_) *δ* 0.88 (t, *J* = 6.7 Hz, 3H, *CH*_*3*_), 1.26 (s, 16H, *CH*_*2*_), 1.36 (m, 2H, ***CH***_***2***_–CH_2_–CH_2_–N_3_), 1.71 (p, *J* = 7.0, 2H, ***CH***_***2***_–CH_2_–N_3_), 3.25 (t, *J* = 7.0, 2H, ***CH***_***2***_–N_3_). ^13^C{^1^H}-NMR (100 MHz, CDCl_3_) *δ* 14.26, 22.84, 26.87, 28.99, 29.31, 29.49, 29.64, 29.70, 29.77, 30.06, 51.64.

#### 2.1.2 1-Nitrododecane (D–NO_2_)

1-Nitrododecane was synthesized using 1-bromododecane and silver nitrite in diethyl ether following known literature procedures.^16^^1^H-NMR (400 MHz, CDCl_3_) *δ* 0.88 (t, *J* = 6.7 Hz, 3H, *CH*_*3*_), 1.26 (s, 16H, *CH*_*2*_), 1.36 (m, 2H, ***CH***_***2***_–CH_2_–CH_2_–NO_2_), 2.00 (p, *J* = 7.1, 2H, ***CH***_***2***_–CH_2_–NO_2_), 4.37 (t, *J* = 7.1, 2H, ***CH***_***2***_–NO_2_). ^13^C{^1^H}-NMR (100 MHz, CDCl_3_) *δ* 14.26, 22.83, 26.36, 27.56, 28.98, 29.40, 29.47, 29.59, 29.72, 29.73, 32.04, 75.90.

#### 2.1.3 Dodecyl nitrate ester (D–ONO_2_)

Fuming nitric acid (20 g) was added to a 250 mL beaker and cooled in an ice bath. To the cooled nitric acid dodecanol (10 g, 54 mmol) was added dropwise. The solution was allowed to stir in the ice bath for 15 minutes. Next sulfuric acid (30 g) was added to the solution dropwise. The solution stirred for one hour at 0 °C and one hour at room temperature. After stirring, the solution was poured into an ice water solution of 250 mL and an oil was observed. The oil was extracted with ethyl acetate (3×, 200 mL). The combined organic layer was then washed with water (2×), aqueous sodium bicarbonate solution (4×), and water again (3×). The organic layer was then dried on magnesium sulfate and filtered. The solvent was removed leaving a clear oil. ^1^H-NMR (400 MHz, CDCl_3_) *δ* 0.88 (t, *J* = 6.7 Hz, 3H, *CH*_*3*_), 1.26 (s, 16H, *CH*_*2*_), 1.36 (m, 2H, ***CH***_***2***_–CH_2_–CH_2_–ONO_2_), 1.71 (p, *J* = 6.9, 2H, ***CH***_***2***_–CH_2_–ONO_2_), 4.44 (t, *J* = 6.7, 2H, ***CH***_***2***_–ONO_2_). ^13^C{^1^H}-NMR (100 MHz, CDCl_3_) *δ* 14.26, 22.84, 25.77, 26.87, 29.26, 29.48, 29.53, 29.64, 29.75, 32.06, 73.63. A LECO Pegasus HRT operated in negative chemical ionization mode with methane gas was used to obtain a high resolution mass spectrum. D–ONO_2_ was detected as [M*]^−^ at a mass of 231.1842 with the theoretical mass being 231.1829, an error of 0.0013 Da.

#### 2.1.4 N-Dodecylnitramide (D–NHNO_2_)

Fuming nitric acid (20 mL) was added to a 250 mL beaker and cooled in an ice bath. Trifluoroacetic anhydride (40 mL) was added slowly to the cooled nitric acid solution. Once fully added the solution was allowed to stir in the ice for 15 minutes. Next dodecylamine (10 gram, 54 mmol) was added slowly to the cooled solution. After addition of the dodecylamine, the solution was stirred in ice for 1.5 hours and then warmed to room temperature and stirred for another 1.5 hours. After stirring, the solution was added to an ice water solution of 500 mL and an oil was observed. The solution was extracted with ethyl acetate (3×, 300 mL). The combined organic layer was then washed with water (2×), aqueous sodium bicarbonate solution (4×), and water again (3×). The organic layer was then dried on magnesium sulfate and filtered. The solvent was removed leaving a white solid material. This was the only synthesized material that resulted in a solid product. Yield = (10.5 g) (80.5%). ^1^H-NMR (400 MHz, CDCl_3_) *δ* 0.88 (t, *J* = 6.8 Hz, 3H, *CH*_*3*_), 1.25 (s, 16H, *CH*_*2*_), 1.38 (m, 2H, ***CH***_***2***_–CH_2_–CH_2_–NHNO_2_), 1.76 (p, *J* = 7.4, 2H, ***CH***_***2***_–CH_2_–NHNO_2_), 3.00 (q, *J* = 7.8, 7.1, 2H, ***CH***_***2***_–NHNO_2_), 7.94 (broad s, 1H, *NHNO*_*2*_). ^13^C{^1^H}-NMR (100 MHz, CDCl_3_) *δ* 14.26, 22.84, 26.74, 29.27, 29.52, 29.66, 29.76, 29.81, 32.07, 40.38. A SCIEX X500R QTOF operated in negative mode with an atmospheric pressure chemical ionization source was used to obtain a high resolution mass spectrum. D–NHNO_2_ was detected as [M + Cl]^−^ at a mass of 265.1469 with the theoretical mass being 265.1688, an error of 0.0219 Da.

#### 2.1.5 Dodecanenitrile

Dodecanenitrile was synthesized according to literature procedures^17^ in order to identify a potential degradation product in the irradiated materials. 1-Bromoundecane (0.5 grams) and sodium cyanide (0.3125 grams) were added to a round bottom flask followed by the addition of 20 mL of DMF. The solution was heated to 100 °C overnight. The next day the solution was cooled to room temperature and diluted with 100 mL of water. The solution was then extracted with hexane (3×). The organic layer was then washed with water (3×). The organic layer was then dried on magnesium sulfate, filtered and the solvent was removed. The product was isolated as a clear oil. ^1^H-NMR (400 MHz, CDCl_3_) *δ* 0.88 (t, *J* = 6.7 Hz, 3H, *CH*_*3*_), 1.26 (s, 14H, *CH*_*2*_), 1.44 (m, 2H, ***CH***_***2***_–CH_2_–CH_2_–C≡N), 1.65 (p, *J* = 7.36, 7.23, 2H, ***CH***_***2***_–CH_2_–C≡N), 2.33 (t, *J* = 7.11, 2H, ***CH***_***2***_–C≡N). ^13^C{^1^H}-NMR (100 MHz, CDCl_3_) *δ* 14.3, 17.3, 22.8, 25.5, 28.8, 28.9, 29.4, 29.6, 29.7, 32.0, 120.0.

### 2.2 Gamma irradiations

Materials for gamma irradiation were first placed into custom built irradiation vessels. Each vessel consisted of a glass ampoule hermetically bonded to a 1.33 in conflat flange (Accuglass) that was connected to a series of VCR fittings (Swagelok) to form a gas tight apparatus (see Fig. S1†). The final vessel had three “arms”: one contained a removable 50 cm^3^ gas bottle and was used for headspace gas analysis; one contained an MKS Baratron Capacitance Manometer; and the last one contained a metal seated valve (Swagelok) that was used to connect to the evacuation manifold before isolating the vessel. An 11.5 psi burst disk (Accuglass) was also connected as a pressure relief in the event of overpressurization. The total free space volume for the vessels before sample addition was approximately 105 cm^3^.

After approximately 2–5 g of the materials were placed into the glass ampoule, the samples were evacuated to a final pressure of 25 hPa using the freeze–pump–thaw methodology. The vessels were then isolated using the valve, transported to the Gamma Irradiation Facility (GIF) at Sandia National Laboratories (SNL), and arranged in an arc around a ^60^Co source (γ-ray energies 1.17 and 1.33 MeV). The dose rate was measured with an ionization chamber and found to be ∼1 Gy s^−1^ with respect to CaF_2_. At this dose rate, the temperature during irradiation would not exceed 30 °C. Materials were irradiated for approximately 84 hours to achieve a total absorbed dose of 300 kGy-CaF_2_ before being transported back to LANL for analysis. The dose was kept consistent with respect to CaF_2_, and a simple conversion of the absorbed dose with respect to the various materials can be found in the ESI (Table S1†). The dose was chosen to induce a large enough change in the material that could be detected using the techniques chosen for analysis, and the dose rate was chosen for convenience with facility availability. No detectable amounts of solids were produced as a result of irradiation with the exception of dodecyl nitramine which was synthesized as a solid. Irradiations were completed twice on each material.

*NOTE:* ^*60*^ *Co is a high energy gamma emitter that can cause serious harm or death. Experiments using* ^*60*^ *Co should only be conducted in an approved facility with trained personnel.*

### 2.3 Experimental investigations

Following the synthesis of suitably pure materials (as determined by NMR spectroscopy), the samples were irradiated and analyzed using a variety of analytical techniques. By using materials with similar purity, the expectation was that the effects of impurities in the samples would be comparable. For all analytical techniques completed on the condensed phase, the focus was simply on the differences between the irradiated and unirradiated materials; impurities in the starting material revealed using more sensitive analytical techniques were not evaluated. Both irradiated and unirradiated materials were analyzed at the same time to ensure the most direct comparison between the samples.

#### 2.3.1 Condensed phase analysis using gas chromatography time-of-flight mass spectrometry (GC-TOFMS)

Trace analysis and identification of the condensed phase was completed using gas chromatography time-of-flight mass spectrometry (GC-TOFMS). This data was obtained using a GC-TOFMS consisting of an Agilent 7890 GC (Agilent Technologies, Palo Alto, CA, USA) equipped with an Agilent 7683 autoinjector coupled to a Pegasus HT (LECO, St. Joseph, MI). The electron impact ionization voltage for the TOFMS was set to −70 eV, with a detector voltage of 1260 V, and the data was collected from 35 amu to 350 amu at unit mass resolution at a rate of 5 Hz. The samples were prepared by dissolving approximately 0.5 g of sample in ∼3 mL of dichloromethane. Prior to injection, dichloromethane was used as a solvent rinse. The transfer line temperature was set to 250 °C and the source was set to 225 °C. The GC column was a Rxi-5 ms stationary phase (Restek, Bellefonte, PA, USA), with a 30 m length, 250 μm inner diameter, and 0.25 μm film thickness. 1 μL volume of the sample was mixture was injected at a split of 10 : 1 with an inlet temperature of 250 °C. Ultra-high purity helium (Grade 5, 99.999%) was used as the carrier gas at a constant flow of 2.0 mL min^−1^. The oven was held at 40 °C for 1.5 min and ramped at 10 °C min^−1^ to 250 °C where it was held for 1 min. 6 replicates of each sample were collected and the resulting data was exported as a .csv file and then imported into Matlab 2021a for visualization and subsequent evaluation using Fisher ratio analysis.^18^ Standards of dodecanenitrile, dodecyl amine, dodecanol, dodecanal, dodecene, octanol, and octanal were also run.

After the data was imported into Matlab, the chromatograms were baseline corrected using in-house software, smoothed using the Savitzky–Golay function native to Matlab, and then normalized to the total ion current. A tile size of 20 data points (4 seconds) was used. A S/N threshold of 3 was applied and the Fisher ratios were calculated at each tile for each *m*/*z*. The average Fisher ratio for each tile and grid scheme was calculated by taking the average Fisher ratio of the top ten *m*/*z* Fisher ratios with the requirement that there be at least 3 *m*/*z* present in the tile above the S/N threshold. If a tile contained at least 3 *m*/*z* ratios above the threshold but fewer than 10 *m*/*z*, then average Fisher ratio was calculated using those m/zs (*e.g.* if there were only 6 *m/z*s above the S/N threshold, then the average Fisher ratio was calculated with those 6 *m/z*s). The redundant hits were removed by using a “pinning and clustering” method with a cluster window of 15 data points (3 seconds).^18,19^ Finally, a statistical threshold was obtained using a combinatorial null distribution. After a chemical change was discovered using Fisher ratio analysis, the resulting spectra was matched to the NIST database to obtain identification and match value where possible.

#### 2.3.2 ^1^H nuclear magnetic resonance (^1^H-NMR) spectroscopy

Quantitative trace analysis was achieved with ^1^H Nuclear Magnetic Resonance (^1^H-NMR) spectroscopy. While ^13^C-NMR spectra were also collected, no discernible differences were seen in these spectra; ^1^H-NMR spectroscopy was found to be uniquely suited for analysis of these systems. NMR spectra were recorded on a 400 MHz Bruker spectrometer. A small amount of material was dissolved in dry chloroform-d_6_ and all peaks were referenced to the residual solvent peak. In order to detect and integrate decomposition at the <1% level, concentrated solutions (∼10 mg/0.5 mL) were prepared for trace analysis using 64 scans and a delay time of 1 second. Spectra were then imported into MestReNova and new signals were integrated with respect to larger signals present in the parent material; in each integration, the peak choice and reason is explained in the text.

#### 2.3.3 Headspace gas analysis using gas chromatography mass spectrometry (GC-MS)

A gas chromatography method was developed to quantify hydrogen and identify unknown compounds in headspace gas samples that were collected during radiolysis. An Agilent Technologies 8890 gas chromatograph equipped with a 5977C mass spectrometer and a thermal conductivity detector (GC-MS/TCD) was used for all quantitative and semi-quantitative analyses of headspace gases (Agilent Technologies, Santa Clara, CA). A Wasson-ECE AS201B gas autosampler was used to inject all samples and calibration standards at consistent pressures (Wasson-ECE Instrumentation, Fort Collins, CO, USA). The autosampler oven and transfer line were heated to 90 °C to facilitate consistent transfer of all gas-phase constituents of interest to the GC columns. Two different analytical columns were used in this work. A CP-volamine capillary column (Agilent J&W) of 60 m length, 0.32 mm i.d., and 7 μm film thickness was connected from the GC valve box to the mass spectrometer, while a ShinCarbon packed column of 2 m length and 1/16′′ o.d. was used with a TCD detector to separate and quantify hydrogen in each sample (Restek Corporation, Bellefonte, PA, USA). Samples were injected at a total volume of 1.25 mL, where 1 mL was sent to the TCD detector and 0.25 mL went to the GCMS. A 4 mL min^−1^ constant flow rate was used for the GCMS flow path, while the following pressure ramp program was used for analysis on the TCD detector: initial pressure 50 psi (2 min hold), to 75 psi at 10 psi min^−1^. Ultra-high purity helium and argon were used as the carrier gas for the GCMS and TCD detector, respectively (Airgas, >99.999%). The oven program was: oven start 40 °C (5 min hold) to 265 °C at 20 °C min^−1^ (5 min hold).

Hydrogen was calibrated using certified ppm-level standards (Airgas). A semi-quantitative method was used to provide mole percent (mol%) level estimates of unknown gas-phase species detected by GC-MS in each sample. Chromatographic peak areas were calculated for all unknown compounds with a library match probability greater than eighty percent. Peak areas of each unknown compound that passed the library match criteria were divided by the total peak area sum for all compounds to obtain a percent-level estimate. Semi-quantitative estimates of unknown compounds were tabulated and reported for each sample. It is important to note that quantified hydrogen was accounted for in each sample prior to calculating an estimated concentration for unknown compounds. The estimated uncertainty for the mol% calculation is 10% of the calculated value.

## 3. Results

In the case of all condensed phase analyses, direct comparisons were made between the irradiated and unirradiated samples. For this study, we chose a single radiation dose, 300 kGy-CaF_2_, in replicate. This allowed us to benchmark the relative stability of the various functional groups without the added complexity of various doses, which would be more appropriate for a follow-on mechanistic study. The initial state of the materials, *i.e.* synthesis impurities, is not considered in the analysis; purity was determined using ^1^H-NMR, and anything above 97% was considered pure enough for this study. Both irradiated and unirradiated samples were analyzed at the same time to provide the best comparison and to ensure that any observed differences could be attributed to irradiation. Less sensitive analyses were completed using Raman and FTIR spectroscopy. No differences were seen for any of the samples, and thus the results are not discussed in the main text. However, all Raman and FTIR spectra can be found in the ESI (Fig. S13–S22†). Headspace gas analysis revealed many different products that were identified using a database or, in the case of hydrogen, with a standard. The total ion chromatographs (TICs) and tables containing the identified products can be found in the SI. This section will only focus on the most relevant results. A full list of GC-MS results can be found in the ESI (Tables S5–S9 and Fig. S23–S27†). The rest of the data used in this paper can also be found in the ESI.†

### 3.1 Dodecane (D–H)

**Fig. 2:**
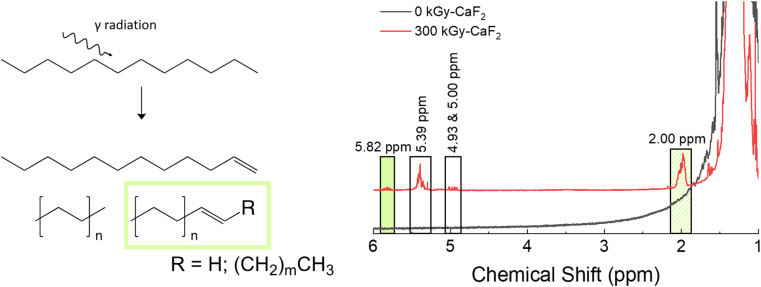
Summary of results for condensed phase analysis of irradiated D–H. Left: Various products identified with GC-TOFMS. Right: Various products identified with ^1^H-NMR spectroscopy.

Unsubstituted dodecane was irradiated to understand how the backbone molecule degraded as well as provide a reference point for the derivatized dodecane materials, and the summary of the results from chemical analyses on the solid phase can be found in Fig. 2. GC-TOFMS coupled with Fisher ratio analysis on the condensed phase revealed a total of 25 statistically significant compounds (*e.g.* hits), though most of these hits had a Fisher ratio below 100 indicating that the chemical changes were minor; this was the case for all the samples analyzed (see Fig. S28–S35 and Tables S10–S13†). This result matches previous results where the limit of discovery is often below the limit of identification.^18,20^ Of the 25 hits, only dodecene was able to be identified through matching of the NIST database as well as confirmation by a dodecene standard, though the exact isomer of dodecene is not known and the hit is likely a mixture of several isomers. The formation of dodecene requires the removal of two hydrogen atoms and the formation of a carbon–carbon double bond. Many others have also found evidence of the formation of carbon double bonds,^12,13,21^ so this result is not surprising. For the other hits, the most significant *m*/*z* was 43, 57, and 71, which correspond with alkane derivatives. These are likely chain scission products, which again has been seen in previous dodecane irradiations.^12,22^

Chain scissions resulting in smaller alkanes yield NMR peaks that fall within the broad dodecane peaks at ∼2 ppm, so ^1^H-NMR spectroscopy was not useful for identifying these products and thus no quantification could be made for them. Instead, a few new peaks were seen in the ^1^H-NMR spectra: a multiplet at 2.00, a doublet of sextets as 4.93, a doublet of quintets at 5.00, a multiplet at 5.39, and a multiplet at 5.82 ppm. Spiking the irradiated sample with dodecene revealed that the multiplet at 5.82 ppm and at least some of the multiplet at 2.00 ppm are due to alkenes. Integrations of all peaks with respect to the large characteristic dodecane multiplets indicative of dodecane yielded an approximate decomposition of 0.1%.

The total radiolytic gas yield, also known as the *G*-value with traditional units of molecules produced per 100 eV of energy absorbed, was found to be 4.2 molecules/100 eV, roughly 29% lower than the previously measured yield for de-aerated dodecane.^11^ The exact reasoning for this is not known but could be due to the scavenging of chemistry-inducing precursors (*i.e.* radicals) due to impurities in the starting material. Although Fisher Chemical did not give a numerical value for the purity of the material, it was likely lower than the 99+% pure material utilized by LaVerne and Kleemola. The yield measured in this study matched better with the aerated dodecane yield measured by LaVerne and Kleemola, so it is also possible that the samples were not evacuated to a high enough vacuum level. GC-MS analysis on the headspace gas revealed the formation of many species, including some that support the incomplete evacuation (see Table S6†). Approximately 64 mol% was hydrogen gas, while a further 16 mol% was identified as volatile alkanes such as isobutane, pentane, heptane, *etc.* that were formed due to chain scission as discussed above. Also seen was roughly 8 mol% of volatile alkenes, which suggests a significant amount of the degradation resulted in the formation of carbon double bonds. Overall, these results indicate that multiple degradation events were likely occurring on a dodecane molecule, though it is certainly possible that some amount of the degradation seen in the gas phase occurred due to gas phase radiolysis. This phenomenon would be the case for all irradiated samples.

### 3.2 Dodecyl azide (D–N_3_)

**Fig. 3:**
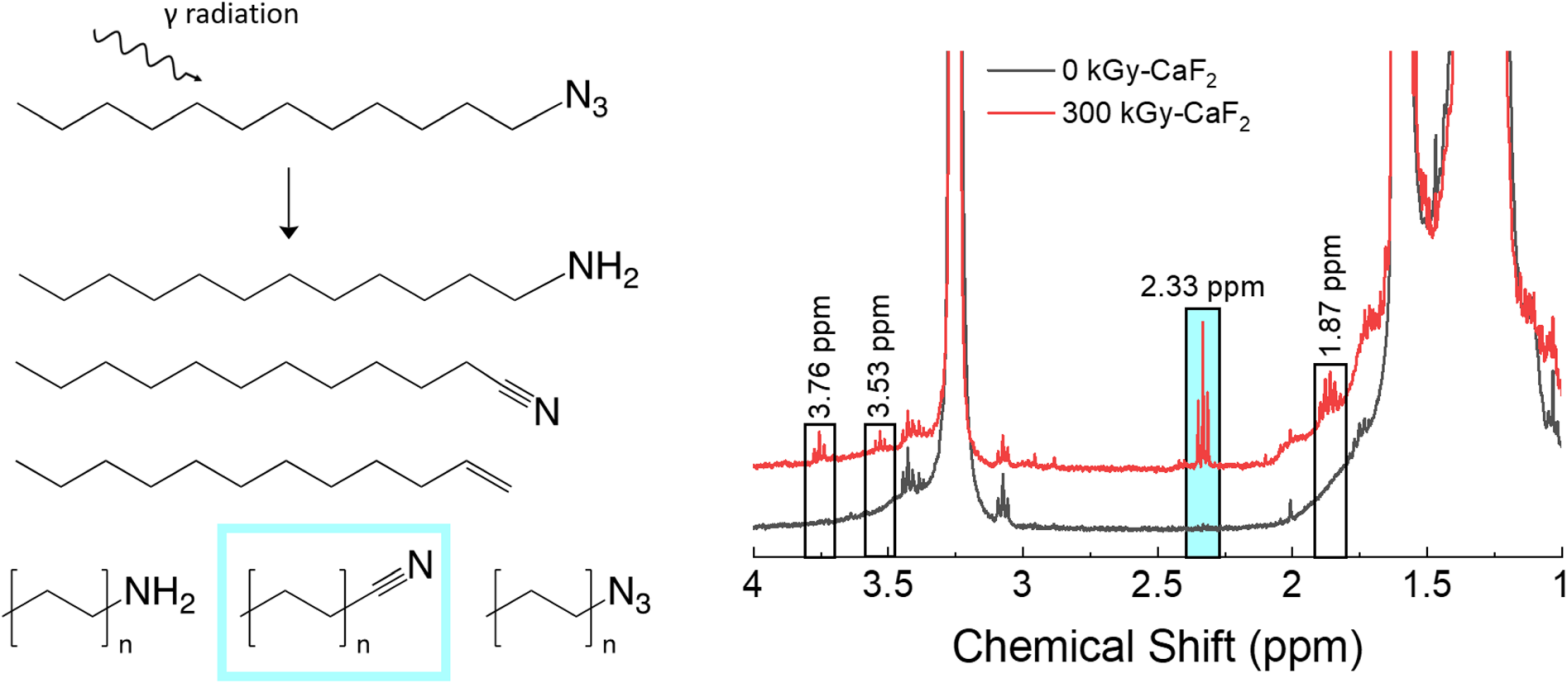
Summary of condensed phase results for irradiated D–N_3_. Left: Various products as identified by GC-TOFMS. Right: Various peaks as identified by ^1^H-NMR spectroscopy.

A summary of the chemical analyses on the condensed phase of irradiated D–N_3_ can be found in Fig. 3. Trace analysis identification with GC-TOFMS and Fisher ratio analysis resulted in 64 hits above the statistical threshold. From these hits, numerous degradation products were identified: dodecene, dodecyl amine, octyl azide, octanenitrile, nonanenitrile, decanenitrile, undecanenitrile, and dodecanenitrile. The identities of dodecene, dodecyl amine, and dodecanenitrile were confirmed with standards. The presence of these compounds indicates scission of part of the FG (nitriles and amine), removal of the entire FG (dodecene), and chain scission (octyl azide) as well as a combination of FG scissions and chain scissions. The identities of the remaining hits are not known.

For the unirradiated D–N_3_, the main identifying NMR signal is a triplet at 3.25 ppm. This triplet is due to the CH_2_ adjacent to the N_3_, and thus significant shifts of this triplet are likely due to changes in the N_3_ FG. Quantitative analysis with ^1^H-NMR spectroscopy revealed several small signals in the baseline: a quintet at 1.86 ppm and triplets at 2.33, 3.53, and 3.76 ppm. Due to the abundance of nitrile species revealed using GC-TOFMS, the ^1^H-NMR spectrum of dodecanenitrile was compared to the irradiated D–N_3_ spectrum. The triplet at 2.33 ppm was confirmed to be the CH_2_ adjacent to the nitrile. Although shorter alkane chains may give measurable peak shifts in ^1^H-NMR spectra, samples containing octanal, decanal, and dodecanal were indistinguishable from one another (see Table S5†). As a result, it was deemed not possible to distinguish the various alkane nitrile species identified using GC-TOFMS. This fact is presumed to be the same for all similar species identified using ^1^H-NMR spectroscopy in this study. Integration of the nitrile triplet with respect to the azide triplet gives an estimated decomposition of approximately 1.9%. Both dodecene and dodecyl amine were analyzed using ^1^H-NMR spectroscopy, but none of the peaks were visible in the irradiated D–N_3_ sample. The identity of the quintet at 1.86 ppm is therefore not known. The 3.53 and 3.76 ppm triplets can be assumed to be related to the 3.25 ppm triplet (*i.e.* corresponding to a change in the FG), though they do not appear to be a nitrile or an amine. In this case, the 3.53 ppm triplet integrates to about a 0.3% degradation while the 3.76 ppm signal integrates to approximately 0.4% degradation.

The radiolytic gas yield of all species for D–N_3_ was found to be 18.6 molecules/100 eV. Of this gas, approximately 4 mol% was hydrogen gas, 49 mol% was volatile alkanes, and 3 mol% was volatile alkenes. Interestingly, there was also a 16 mol% contribution due to cyclopentane and a 1 mol% contribution due to cyclopentene, both of which are ring structures and not seen in large quantities in the headspace gas of irradiated dodecane. The N_2_ content was not able to be assessed with this technique, though N_2_ is thought to be a large fraction of the headspace gas. The ratio of alkanes to alkenes was also significantly different from dodecane as was the mol% of hydrogen gas. Overall, these results suggest that a significant portion of the degradation occurred on the energetic FG, and that the degradation seen with D–N_3_ is not simply a combination of the degradation of the dodecane backbone and the degradation of the azide energetic FG.

### 3.3 Dodecyl nitro (D–NO_2_)

**Fig. 4:**
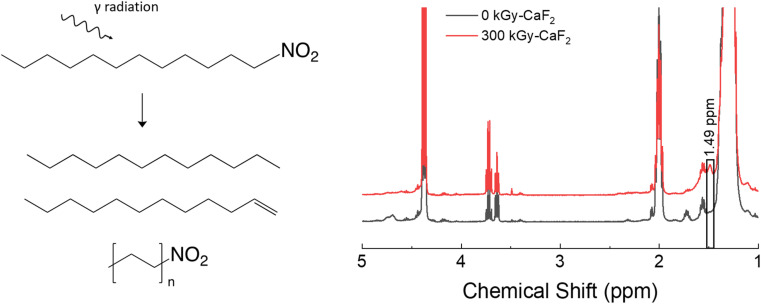
Summary of results for the condensed phase analysis of irradiated D–NO_2_. Left: Various products identified by GC-TOFMS. Right: ^1^H-NMR spectra for both samples.

A summary of the condensed phase chemical analyses performed on D–NO_2_ is shown in Fig. 4. GC-TOFMS analysis resulted in a total of 80 hits above the statistical threshold, but only 3 products were able to be identified: dodecene, dodecane, and hexyl nitro. Both dodecene and dodecane indicate a removal of the nitro energetic FG while the hexyl nitro indicates a chain scission. It should be noted that the match value for the hexyl nitro was not very high (849) and no other nitrated alkane was found in the NIST database making the identification tentative, though the product is positively identified as a product of chain scission due to the characteristic *m*/*z* signals. It could therefore be a longer chain nitro alkane, but the conclusion is still the same regardless of the chain length.

^1^H-NMR spectroscopy did not reveal meaningful changes. Of the identified hits on GC-TOFMS, only dodecene would result in a visible change to the ^1^H-NMR spectra. However, no peaks belonging to dodecene could be discerned from the baseline; a broad signal at 1.49 ppm was the only visible change, and the identity of this peak is not known. Given the lack of meaningful results, integration of the quantity of degradation products was not possible with D–NO_2_.

Gas analysis of the radiolytically-produced headspace gas gave a total yield of 3.8 molecules/100 eV, the lowest yield of any of the samples. GC-MS identified 22 mol% was hydrogen gas while 6 mol% was identified as volatile alkenes and 3 mol% was identified as volatile alkanes. The largest contributor was nitrous oxide at 39 mol%, followed by an amine at 24 mol%. Nitrous oxide has also been seen as a major component in the gas phase of irradiated explosives^23–25^ containing the nitro energetic FG, so this result was expected. Overall, these results indicate that D–NO_2_ is a relatively stable molecule to irradiation when compared to the other molecules used in this study, though degradation of the nitro energetic FG is a large contributor to the degradation gas products observed.

### 3.4 Dodecyl nitrate ester (D–ONO_2_)

**Fig. 5:**
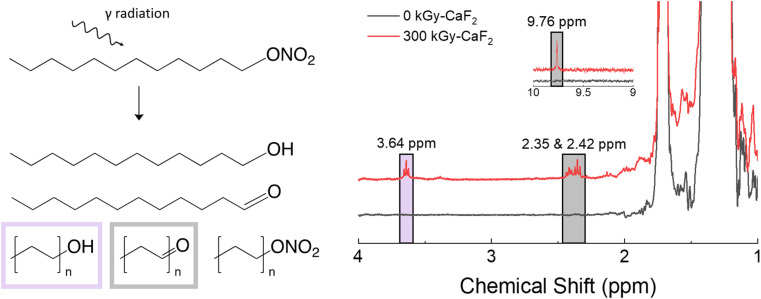
Summary of condensed phase chemical analysis results for irradiated D–ONO_2_. Left: Various products identified with GC-TOFMS. Right: Products identified with ^1^H-NMR spectroscopy.

A summary of the results from the condensed phase chemical analyses completed on the D–ONO_2_ system is shown in Fig. 5. Trace analysis with GC-TOFMS revealed a total of 57 hits above the statistical threshold, and several were able to be identified: octanal, decanal, undecanal, dodecanol, undecanol, heptyl nitrate ester, octyl nitrate ester, and nonyl nitrate ester. The aldehydes and alcohols indicate a scission along the O–NO_2_ bond of the nitrate ester where the NO_2_ is released but the O remains with the parent molecule. This bond is referred to as the trigger linkage, or weakest bond, for the nitrate ester FG^26^ and it is thought to be the first bond to break during thermal and explosive decomposition.^27,28^ Interestingly, GC-TOFMS did not identify products indicative of the removal of the entire nitrate ester energetic FG (scission of the C–ONO_2_ bond). Shorter chain nitrate esters were seen, indicating chain scission was also a significant degradation product.

^1^H-NMR spectroscopy showed the formation of several new species: triplets at 2.35, 2.42, 3.64, and a small singlet at 9.76 ppm. The CH_2_ group next to the nitrate ester FG has a characteristic triplet at 4.47 ppm, so significant shifts in that triplet are likely due to changes in the nitrate ester FG. Based on the GC-TOFMS results, samples were spiked with octanol and octanal to discern the location of the alcohol and aldehyde peaks, respectively. The triplet at 3.64 ppm was identified as an alcohol signal through spiking with an independently prepared sample and integrated to 0.8% of the parent triplet at 4.47 ppm. Similarly, the triplet peaks at 2.35 and 2.42 ppm as well as the singlet at 9.76 ppm were identified as aldehyde signals and integrated to ∼2.2% of the parent triplet.

The total radiolytic gas yield of D–ONO_2_ was measured at 20.6 molecules/100 eV, the largest yield of the materials used in this study. GC-MS revealed 3 mol% of that gas was hydrogen gas, 2 mol% was volatile alkenes, and 4 mol% was volatile alkanes. A further 12 mol% was due to volatile hydrocarbons containing at least oxygen, of which 8 mol% contained aldehyde and 1 mol% contained alcohol groups. Lastly, 1 mol% was due to volatile nitrate ester-containing hydrocarbons, and 75 mol% was due to nitrous oxide. The relative ratios of backbone degradation to FG degradation suggests that a large majority of the radiolytic degradation of D–ONO_2_ occurs on the energetic FG. Overall, these results indicate that D–ONO_2_ is particularly susceptible to radiolytic degradation, and most of that degradation occurs on the nitrate ester FG. Furthermore, the degradation tends to occur along the trigger linkage and leads to the formation of alcohol and aldehyde FGs, though aldehyde formation is more common.

### 3.5 Dodecyl nitramine (D–NHNO_2_)

**Fig. 6:**
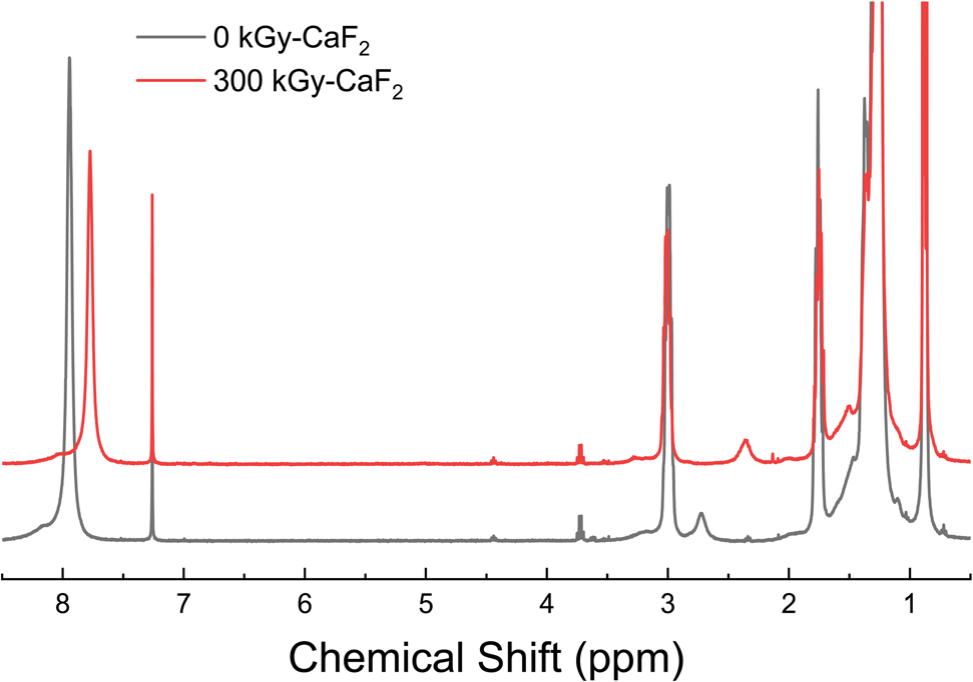
^1^H-NMR spectra for control (bottom black line) and irradiated (top red line) D–NHNO_2_.

D–NHNO_2_ was a solid at ambient temperature and pressure and did not vaporize below 250 °C, so GC-TOFMS could not be completed on the material. Surprisingly, the ^1^H-NMR spectra (shown in Fig. 6) resulted in the broad peak at 7.94 ppm (*NHNO*_*2*_) shifting to 7.77 ppm following irradiation rather than visible changes in the baseline. Addition of acid to the NMR sample of unirradiated D–NHNO_2_ resulted in shifts in the opposite direction, suggesting that the change is not due to the formation of an acid from the release of H^+^. The only insightful data that could be collected was the radiolytically-produced gas. The total gas yield was calculated to be 16.6 molecules/100 eV, and GC-MS revealed 2 mol% was due to hydrogen gas, <1 mol% was due to volatile alkenes, and 3 mol% was due to volatile alkanes. A further 40 mol% was due to partial degradation of the nitramine FG, of which 37 mol% was identified as an amine. 26 mol% was attributed to nitrous oxide, and 19 mol% was attributed to oxygen gas. From the gas analysis, it appears that the energetic FG is once again more susceptible to radiolytic degradation than the backbone molecule is.

## 4. Discussion

The purpose of this study was to assess the relative radiation stability of various energetic FGs by utilizing a common molecular backbone. To that degree, the level of degradation to the backbone molecule is not entirely important, though the ratio of energetic FG degradation and backbone degradation can give some interesting insights. In the following section, we will discuss comparisons of gas production for dodecane *versus* the substituted dodecanes in order to estimate how the energy is localized on the overall molecule as well as the energetic functional group.

Radiolytic gas yields are often used as an indication of the radiolytic activity of a material. In the case of these materials, many gas species were created and identified. If only the total *G*-value is considered, then the stability of the studied substituted molecules from most to least susceptible to radiation is: D–ONO_2_ → D–N_3_ → D–NHNO_2_ → D–NO_2_. To the extent that ^1^H-NMR spectroscopy results could be collected, the integrated amount of decomposition products relative to the parent peaks show a similar trend to the total as yield analysis, as shown in Table 1; the uncertainties listed are systematic uncertainties that were propagated through for the *G*-value calculation. Interestingly, the order of the results matches well with both photolytic molecular dynamics simulations of these molecules at 8 eV^14^ as well as the general trend for the sub-shock impact sensitivity of various explosives.^31–33^ Although a more limited study, these results also provide an explanation why, for a previous study exploring radiolytic damage to a variety of explosives,^25^ the only explosive that was found to degrade with low-level radiation in large enough quantities to be detectable using ^1^H-NMR spectroscopy was pentaerythritol tetranitrate (PETN), a nitrate ester-based explosive. The total gas yields, however, are a measure of how radiolytically active the entire molecule is rather than just the energetic FG. Based on the amount of data able to be collected for these systems, the total gas yield is the only value to give results across the entire substitution series and thus is the only true means of comparison. It is important to note that this simplistic analysis will convolute factors such as the different phase of D–NHNO_2_ (solid) with respect to the other materials (liquid) and the synthesis impurities of the various materials, both of which could have an effect on the results. However, as the general trend seen in these results closely follows the trend seen in photolytic simulations and sub-shock impact sensitivity tests, the total *G*-value seems to be an overall valid approach to ranking the relative radiolytic susceptibility of the investigated energetic FGs.

**Table 1:** Decomposition results from ^1^H-NMR spectroscopy and GC-MS on the gas phase^a^

Molecule	^1^H-NMR decomposition (%) [does not include chain scissions]	*G*-value (molecules/100 eV) [total gas yield]
D–H	0.1	4.2 ± 0.2
D–N_3_	2.6	18.6 ± 0.8
D–NO_2_	Below detection limit	3.8 ± 0.2
D–ONO_2_	3.0	20.6 ± 0.9
D–NHNO_2_	Inconclusive^b^	16.6 ± 0.7

a Note: D = C_12_H_25_.

b Meaningful data was not able to be obtained from the ^1^H-NMR spectra of D–NHNO_2_.

For the dodecane backbone, most of the degradation involves the removal of H atoms which forms both hydrogen gas as well as various alkenes. This result has been seen in previous studies, as well.^11,22,29,30^ Carbon–carbon bonds do also break, as evidenced by the presence of shorter chain alkanes and alkenes, though to a lesser degree. All the substituted dodecane samples showed both alkanes and alkenes being formed, though these were often at a much lower rate than degradation to the energetic FG. Degradation to the dodecane backbone was approximated by the amount of hydrogen gas produced during irradiation; C–C bond breaks were left out of the analysis due to the uncertainty of which side of the molecule the break occurred on and how or when the FG was involved. As a result, the best gas species for tracking where the damage is occurring was determined to be hydrogen. Hydrogen is a major component of the dodecane backbone and is not present in any FG except for the nitramine. Therefore, although some amount of H_2_ gas in irradiated D–NHNO_2_ could come from the nitramine FG, most of it likely comes from the dodecane backbone, and all hydrogen from the other materials must come from the backbone. Hydrogen gas is therefore considered to be evidence of alkane backbone degradation.

Using hydrogen gas as a comparison, the substituted dodecane materials produced only 12–31% the amount of hydrogen gas that pure dodecane produced. This result suggests that the addition of an energetic FG actually reduces the damage to the dodecane backbone structure even though the overall degradation to the molecule generally increases. There is a difference in the energy directly absorbed by each functional group (see ESI†), however the degradation trend suggests that these differences are small in comparison to other factors such as energy transport. One possible explanation for the above observation is exciton migration. During γ-radiolysis, both ionizations and excitations are created throughout the entire system. Excitations can lead to the formation of excitons that operate as an energy transfer mechanism and have been found to be an important mode of energy transport in alkanes.^34^ In fact, *n*-alkanes are considered to be efficient molecular exciton transporters even including the transport of excitons between molecules.^29,30,35^ Scission of the C–H bond in alkanes is the most likely degradation pathway,^11,22^ and this process is thought to occur quickly.^22^ C–C excitons, on the other hand, would be free to travel along and beyond the length of the molecule, assuming the molecule maintains the alkane characteristics. In the case of the substituted dodecane molecules, however, the energetic FG would likely prevent the exciton from moving to another molecule by acting as an exciton trap. The excitation energy would therefore be located on the energetic FG where degradation could occur. Exciton migration and localization on the energetic FG was also seen with non-adiabatic excited state molecular dynamics (NEXMD) calculation performed on the same molecules.^14^ By concentrating the energy on the energetic FGs, less degradation of the dodecane backbone could occur and thus most of the H_2_ gas measured must be due to direct interactions with the C–H bonds of the dodecane molecule. Another possible explanation for the decrease in the hydrogen gas *G*-value for the substituted dodecane molecules with respect to pure dodecane involve the general delocalization of the molecular exciton in alkanes rather than localized transport.^36^ Electron paramagnetic resonance (EPR) spectroscopy has revealed that the *n*-alkane cations are delocalized along the entire length of the alkane chain.^37^ In this instance, the exciton would simply need to couple to the energetic FG to induce damage on it. In either explanation, however, energy does appear to localize on the energetic FG regardless of where the energy is initially deposited, and this localization results in a larger fraction of damage on the FG than on the molecular backbone.

The exact location of the degradation on the energetic FG is also worth noting. For D–N_3_, GC-TOFMS results indicate that the azide FG is broken both at the C–N_3_ and the CN–N_2_ bonds. The N–N bond is the trigger linkage for the azide FG^26,38^ and is considered to be the most susceptible bond to be broken. GC-TOFMS results for D–NO_2_ indicate only the removal of the NO_2_ FG; the C–N bond is the trigger linkage for the nitro FG.^26^ This result is consistent with the observation that the irradiation of trinitrotoluene (TNT) only revealed the formation of dinitrotoluene^23^ which would be formed by the removal of a single NO_2_ group from the molecular structure. As mentioned earlier in the text, D–ONO_2_ was found to degrade only along the O–NO_2_ bond which is the trigger linkage for the nitrate ester FG. This bond has also been found to be particularly susceptible to damage in radiolytic,^23,25^ thermal,^39–41^ and explosive insult^42,43^ degradation events. No instances were observed with the loss of –ONO_2_; instead, the damage to the nitrate ester FG appears to be exclusively through the N–O bond. Though condensed phase analysis for D–NHNO_2_ were unsuccessful, the gas phase analysis reveals at least a partial degradation of the nitramine FG. It is unclear where exactly the bond is breaking for these products, though radiolytic degradation with X-rays of the nitramine-containing explosive RDX suggests the most likely bond to break is the N–N bond^44^ which is the trigger linkage for the nitramine FG.^26^

It is interesting to compare the above results with the 8 eV results found in the photolytic NEXMD calculations completed on the same molecules.^14^ Of course, radiolysis and photolysis are not the same process, but a significant portion of the primary decomposition in alkanes can be attributed to low-energy electronically excited states generated through absorption of scattered radiation.^22^ Additionally, the photolytic NEXMD calculations allow for a look at the fast time scales for degradation involving the *S*_1_ excited state, an insight that is unachievable for the radiolysis experiments in this study. The photolytic NEXMD calculations were also completed on the same molecules utilized in this study, which allows for a more direct comparison between the computational results and these experimental results. In the 8 eV computations involving the substituted dodecane molecules, the exciton was initially localized on the dodecane backbone where it then quickly relaxed to the *S*_1_ excited state and moved to the energetic FG to undergo degradation.^14^ For the D–N_3_ molecule, the degradation would mostly involve the scission of the N–N bond, but contributions involving the scission of the C–N_3_ as well as degradation to the dodecane backbone were also seen. For the D–NO_2_ molecule, only the C–NO_2_ bond was broken. The D–ONO_2_ molecule underwent scission of the O–NO_2_ bond but would sometimes also include some degradation to the dodecane backbone, and the D–NHNO_2_ molecule would either break at the N–N bond or it would remove a hydrogen ion from the nitramine; the ion nature was simply due to how the calculations were completed. In all cases, the primary degradation pathways were in line with what has been seen in this experimental investigation. Obviously, higher energy radiation results in additional degradation pathways, but much of the degradation can be explained by lower energy excitations. This result also follows for what is currently known about the radiolysis of explosives: most of the degradation occurs not on the carbon molecular structure supporting the energetic FGs, but rather on the energetic FGs themselves, and particularly along the trigger linkage.^23,25,44^

## 5. Conclusions

In order to study the effects of ionizing radiation on various commonly used energetic functional groups, a selection of molecules were synthesized with a common molecular backbone and irradiated with γ-rays using a ^60^Co source. The studied materials included: dodecyl azide, dodecyl nitro, dodecyl nitrate ester, and dodecyl nitramine. These materials were irradiated under vacuum and analyzed post-radiolysis which was directly compared to the unirradiated materials. Trace analyses revealed the formation of several species not originally present in the sample that were indicative of chain scission, trigger linkage scission, removal of the entire energetic functional group in the case of some materials, and a combination of all of the above. Analysis of the radiolytically-produced gas revealed that a significant portion of the damage took place on the energetic functional group, specifically along the trigger linkage. A cursory ranking of the radiolytic susceptibility was determined using the total gas *G*-value and was found to be D–ONO_2_ → D–N_3_ → D–NHNO_2_ → D–NO_2_ when ranked from most to least susceptible. These results have some caveats, but do follow the same trend that photolytic calculations and sub-shock impact tests show which lends credence to this approach.

## Author contributions

Patricia L. Huestis – conceptualization, investigation, writing-original draft, visualization. Nicholas Lease – investigation, resources, writing-review & editing. Chris E. Freye – investigation, writing – review & editing. Daniel L. Huber – investigation, writing-review & editing. Geoff Brown – supervision. Daniel L. McDonald – resources. Tammie Nelson – supervision, writing-review & editing. Chris Snyder – resources. Virginia W. Manner – conceptualization, writing-review & editing, supervision, funding acquisition.

## Conflicts of interest

There are no conflicts to declare.

## Supplementary Material

RA-013-D3RA00998J-s001
