# Supplementary material for: Radiolytic degradation of dodecane substituted with common energetic functional groups
Source: RSC Adv. 2023 Mar 21;13(14):9304–15. doi: 10.1039/d3ra00998j (PMC10028498; doi:10.1039/d3ra00998j)
Supplement: RA-013-D3RA00998J-s001 [file RA-013-D3RA00998J-s001.pdf]

Electronic Supplementary Material (ESI) for RSC Advances.
This journal is © The Royal Society of Chemistry 2023

Supporting Information for Radiolytic Degradation of Dodecane Substituted with Common Energetic Functional Groups

Patricia L. Huestis, Nicholas Lease, Chris E. Freye, Daniel L. Huber, Geoffrey W. Brown, Daniel L. McDonald, Tammie Nelson, Christopher J. Snyder, Virginia W. Manner*

CONTENTS:

Irradiation vessel design

Dose corrections for each molecule

Fractional energy absorption for functional groups

Synthesis reaction schemes

^{1}H-NMR spectra

- Control dodecane (D-H)
- Irradiated dodecane (D-H)
- Control dodecyl azide (D-N_3)
- Irradiated dodecyl azide (D-N_3)
- Control dodecyl nitro (D-NO_2)
- Irradiated dodecyl nitro (D-NO_2)
- Control dodecyl nitrate ester (D-ONO_2)
- Irradiated dodecyl nitrate ester (D-ONO_2)
- Control dodecyl nitramine (D-$NHNO_2$)
- Irradiated dodecyl nitramine (D-$NHNO_2$)
- Table with major ^{1}H-NMR signals for control samples
- Table with major ^{1}H-NMR signals for irradiated samples
- Table with major ^{1}H-NMR signals for HPLC-identified species

^{13}C-NMR spectra

- Control dodecane (D-H)
- Irradiated dodecane (D-H)
- Control dodecyl azide (D-N_3)
- Irradiated dodecyl azide (D-N_3)
- Control dodecyl nitro (D-NO_2)
- Irradiated dodecyl nitro (D-NO_2)
- Control dodecyl nitrate ester (D-ONO_2)

- Irradiated dodecyl nitrate ester (D-ONO_2)
- Control dodecyl nitramine (D-$NHNO_2$)
- Irradiated dodecyl nitramine (D-$NHNO_2$)

Raman spectra

- Control dodecane (D-H)
- Irradiated dodecane (D-H)
- Control dodecyl azide (D-N_3)
- Irradiated dodecyl azide (D-N_3)
- Control dodecyl nitro (D-NO_2)
- Irradiated dodecyl nitro (D-NO_2)
- Control dodecyl nitrate ester (D-ONO_2)
- Irradiated dodecyl nitrate ester (D-ONO_2)
- Control dodecyl nitramine (D-$NHNO_2$)
- Irradiated dodecyl nitramine (D-$NHNO_2$)

FTIR spectra

- Control dodecane (D-H)
- Irradiated dodecane (D-H)
- Control dodecyl azide (D-N_3)
- Irradiated dodecyl azide (D-N_3)
- Control dodecyl nitro (D-NO_2)
- Irradiated dodecyl nitro (D-NO_2)
- Control dodecyl nitrate ester (D-ONO_2)
- Irradiated dodecyl nitrate ester (D-ONO_2)
- Control dodecyl nitramine (D-$NHNO_2$)
- Irradiated dodecyl nitramine (D-$NHNO_2$)

GC-MS of headspace gas

- Irradiated dodecane (D-H)
- Irradiated dodecyl azide (D-N_3)
- Irradiated dodecyl nitro (D-NO_2)
- Irradiated dodecyl nitrate ester (D-ONO_2)
- Irradiated dodecyl nitramine (D-$NHNO_2$)

GC-TOFMS of condensed phases

- Control dodecane (D-H)
- Irradiated dodecane (D-H)
- Control dodecyl azide (D-N_3)
- Irradiated dodecyl azide (D-N_3)

- Control dodecyl nitro (D-NO_2)
- Irradiated dodecyl nitro (D-NO_2)
- Control dodecyl nitrate ester (D-ONO_2)
- Irradiated dodecyl nitrate ester (D-ONO_2)
- Control dodecyl nitramine (D-$NHNO_2$)
- Irradiated dodecyl nitramine (D-$NHNO_2$)

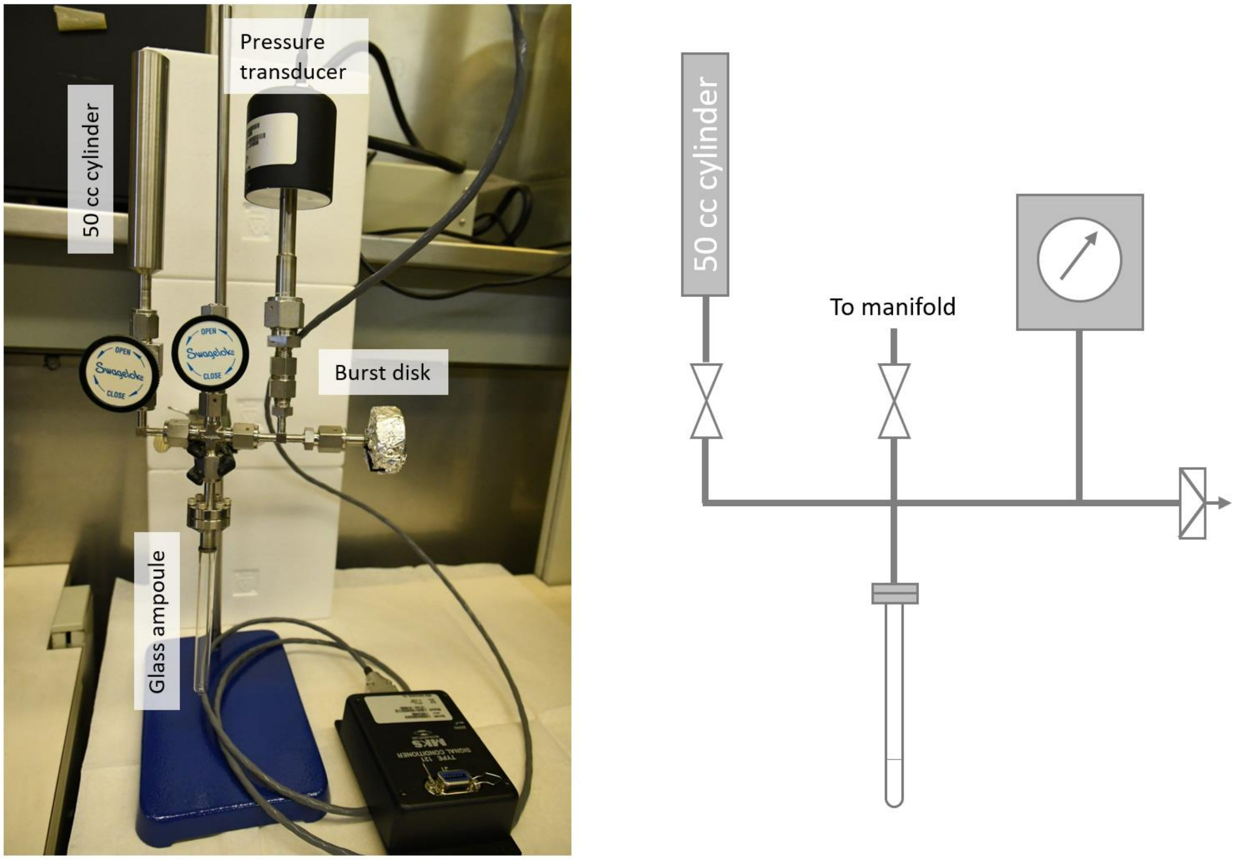

Figure S1: Irradiation vessel (left) along with the schematic (right).

The irradiation vessel is shown in Figure S1. The vessel consisted of 3 “arms”: one with a removable 50 cm^3 gas cylinder for headspace gas analysis, one with a pressure transducer, and one which was used to connect the vessel to the manifold for evacuation. A burst disk was also connected to alleviate pressure in the event too much gas was created, and the sample was loaded into a glass ampoule that was connected and held upright to ensure the sample did not travel back up into the vessel.

Table S1: Corrected absorbed dose values and the mass-energy coefficients used for the dose correction. The mass energy-absorption coefficients were calculated using the NIST Standard Reference Database 126[1] for a photon energy of 1.25 MeV.

**Compound**	**Mass Energy-Absorption Coefficient [μ_{en}/ρ] (cm^2/g)**	**Corrected Dose (kGy)**
Calcium Fluoride	$2.591 * 10^{-2}$	300
Dodecane	$3.075 * 10^{-2}$	356
Dodecyl Azide	$2.984 * 10^{-2}$	344
Dodecyl Nitrate Ester	$2.957 * 10^{-2}$	342
Dodecyl Nitro	$2.9768* 10^{-2}$	345
Dodecyl Nitramine	2.970s * 10^{-2}	344

The above numbers were computed using the mass energy-absorption coefficients (μ_{en}/ρ) which are used to calculate the dosimetric quantity known as kerma (**k**inetic **e**nergy **r**eleased per unit **ma**ss). We can use this same method to determine the relative amount of energy absorbed by each energetic functional group. The mass energy-absorption coefficients were evaluated at 1.25 MeV (the average of the two photons emitted by ^{60}Co as was done in Table S1) and the contributions of the backbone and the energetic functional group were separated out, giving the results shown in Table S2.

Table S2: Values for the fractional energy absorption by direct interaction for the various substituted dodecanes used in this study.

Compound	μ_{en}/ρ – backbone (cm^2/g)	μ_{en}/ρ – functional group (cm^2/g)	Energy absorption % - backbone	Energy absorption % - functional group
D-N_3	$2.453 * 10^{-2}$	$0.531 * 10^{-2}$	82	18
D-NO_2	$2.408 * 10^{-2}$	$0.570 * 10^{-2}$	81	19
D-ONO_2	$2.241 * 10^{-2}$	$0.715 * 10^{-2}$	76	24
D-$NHNO_2$	$2.251 * 10^{-2}$	$0.719 * 10^{-2}$	76	24

From the results shown in Table S2, there does appear to be more energy directly absorbed by the functional groups in D-ONO_2 and D-$NHNO_2$ than in D-N_3 and D-NO_2. However, from the results in the main text, we know that the largest amount of degradation was seen in D-ONO_2, followed by D-N_3, D-$NHNO_2$, and finally D-NO_2. The trend we saw with stability therefore does not seem to be explainable by how much energy was directly absorbed by the functional group. Instead, energy transfer seems to play a larger role.

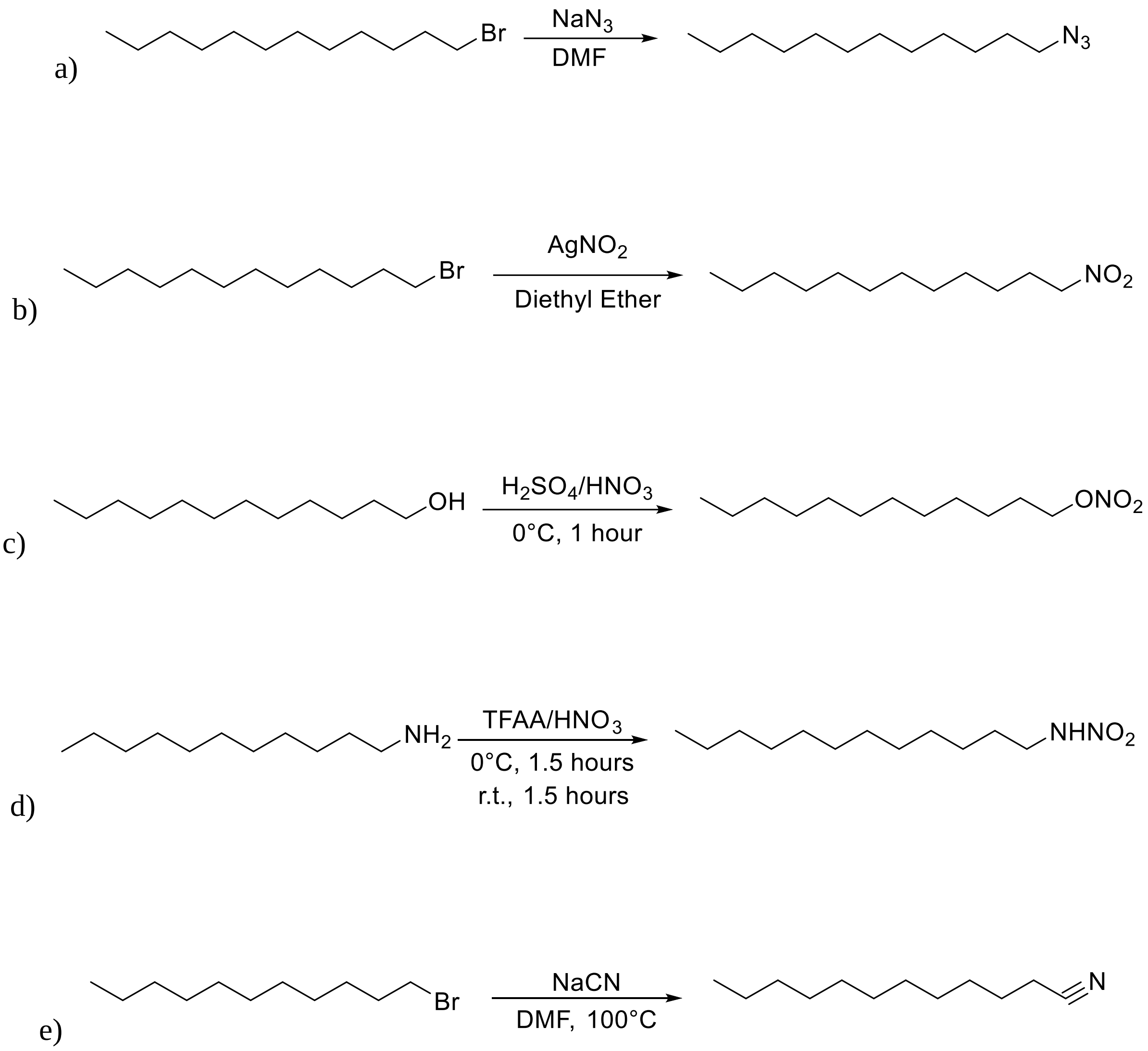

Figure S2: Synthesis reaction schemes for: a) D-N_3, b) D-NO_2, c) D-ONO_2, d) D-$NHNO_2$, and e) dodecanenitrile.

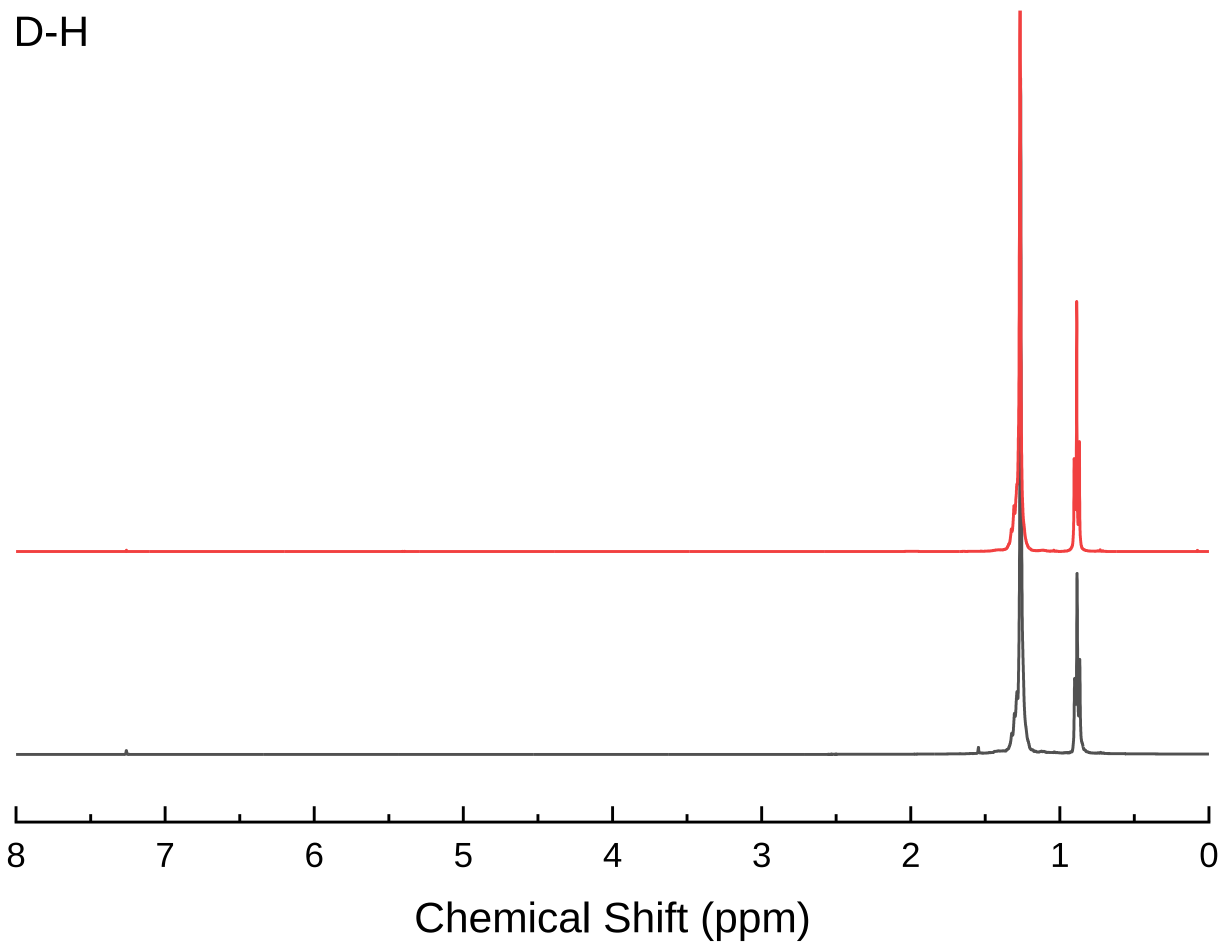

Figure S3: Control (bottom black line) and irradiated (top red line) ^{1}H-NMR spectra of D-H.

The ^{1}H-NMR spectrum for D-H showed the expected peaks at 0.88 (t, J = 6.7 Hz, 6H, CH_3) and 1.26 (s, 20H, CH_2) ppm. Irradiation to 300 kGy-CaF_2 did produce new signals, as described in the main text.

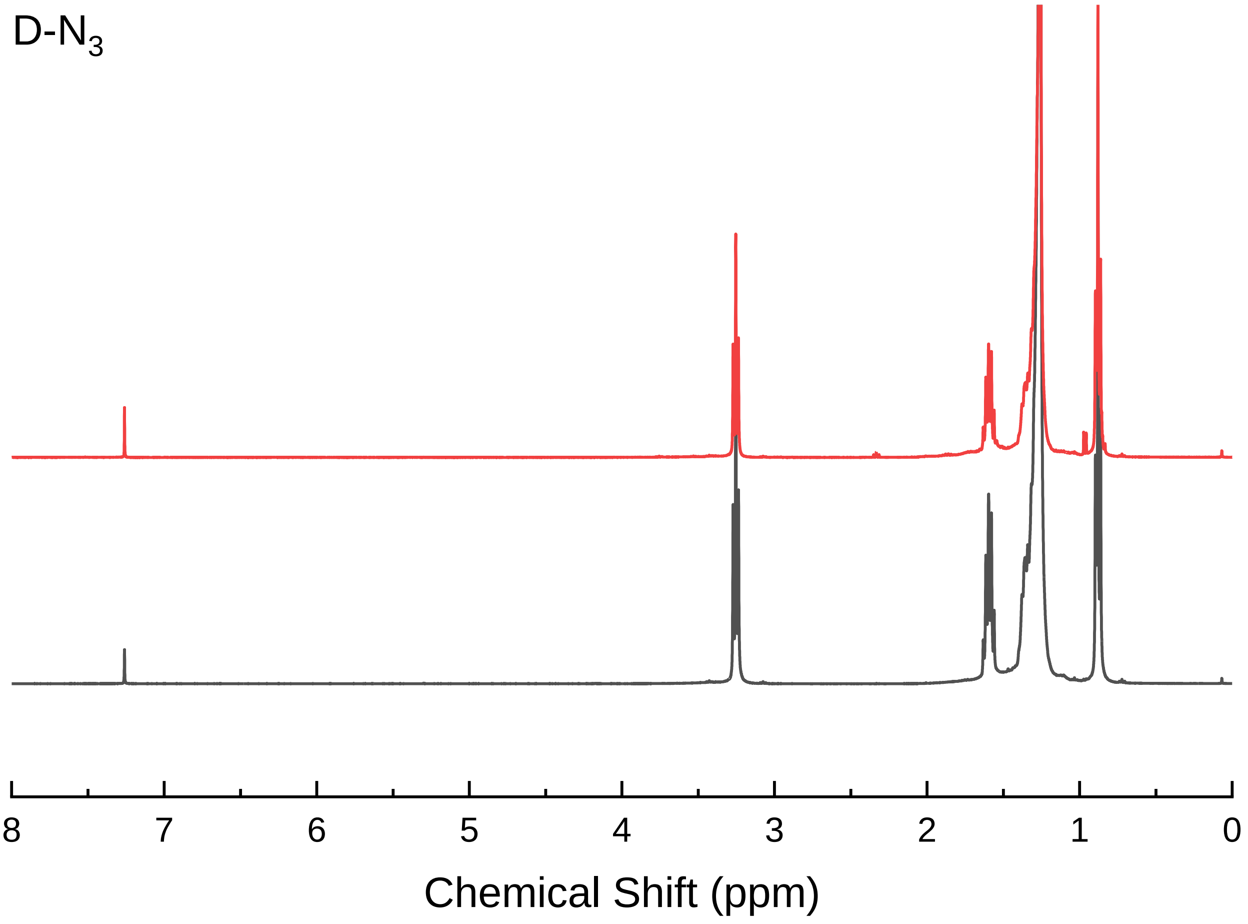

Figure S4: Control (bottom black line) and irradiated (top red line) ^{1}H-NMR spectra of D-N_3.

The ^{1}H-NMR spectrum for D-N_3 showed the expected peaks at 0.88, 1.26, 1.36, 1.71, and 3.25 ppm. Irradiation to 300 kGy-CaF_2 did produce new signals, as described in the main text.

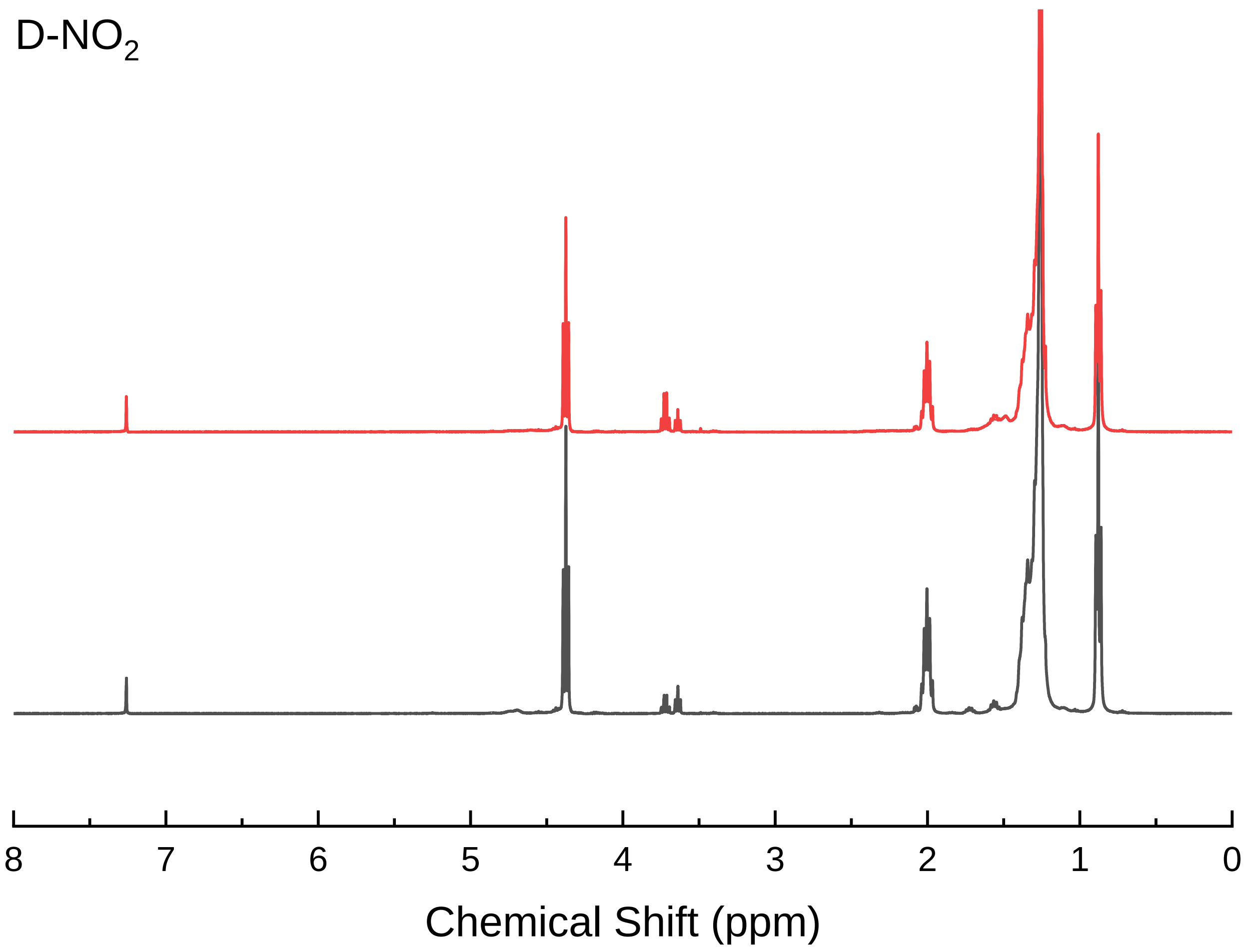

Figure S5: Control (bottom black line) and irradiated (top red line) ^{1}H-NMR spectra of D-NO_2.

The ^{1}H-NMR spectrum for D-NO_2 showed the expected peaks at 0.88, 1.26, 1.36, 2.00, and 4.37 ppm. Irradiation to 300 kGy-CaF_2 did produce new signals, as described in the main text.

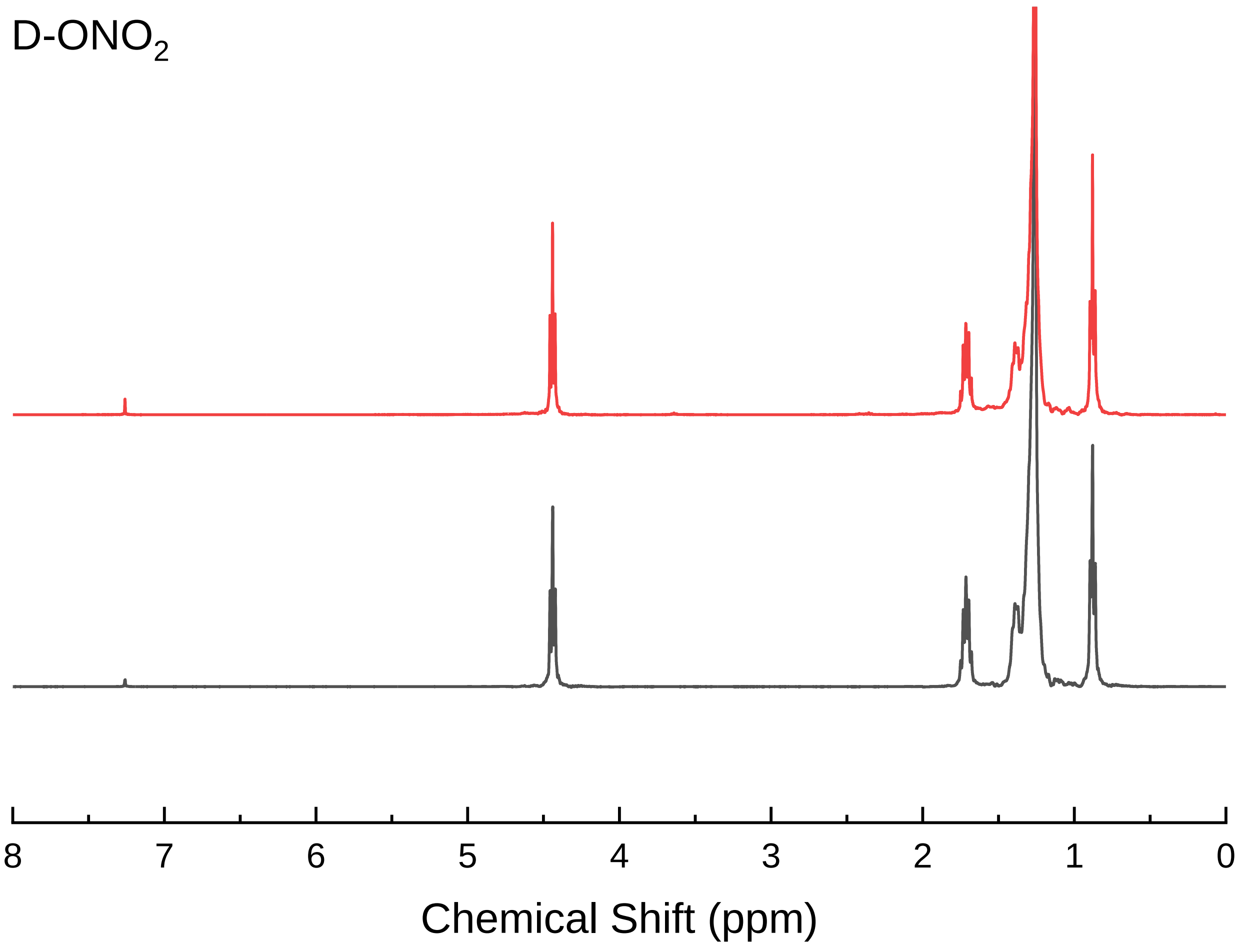

Figure S6: Control (bottom black line) and irradiated (top red line) ^{1}H-NMR spectra of D-ONO_2.

The ^{1}H-NMR spectrum for D-ONO_2 showed the expected peaks at 0.88, 1.26, 1.36, 1.71, and 4.44 ppm. Irradiation to 300 kGy-CaF_2 did produce new signals, as described in the main text.

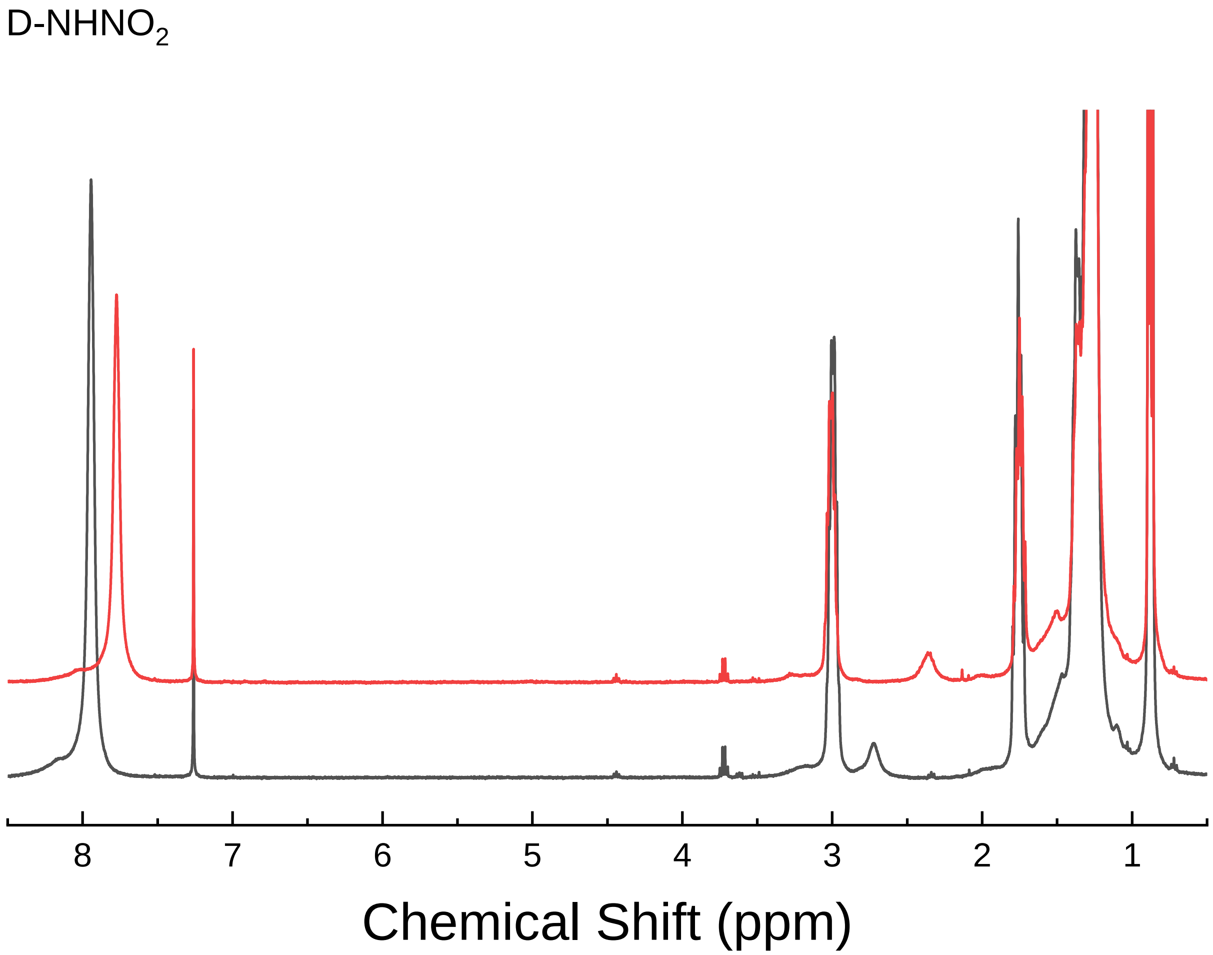

Figure S7: Control (bottom black line) and irradiated (top red line) ^{1}H-NMR spectra of D-NHNO$_2$.

The ^{1}H-NMR spectrum for D-NHNO$_2$ showed the expected peaks at 0.88, 1.25, 1.38, 1.76, 3.00, and 7.94 ppm. Irradiation to 300 kGy-CaF$_2$ produced changes, as described in the main text.

Table S3: Summary of characteristic ^{1}H-NMR spectroscopy peaks for studied materials.

MOLECULE	^{1}H-NMR PEAK	ASSIGNMENT	NMR COUPLING	GC-TOFMS IDENTIFIED
**D-N_3**	1.36 ppm	***CH_2***-CH_2-CH_2-N_3	m	D-N_3
	1.71 ppm	***CH_2***-CH_2-N_3	p	
	3.25 ppm	***CH_2***-N_3	t	
**D-NO_2**	1.36 ppm	***CH_2***-CH_2-CH_2-NO_2	m	D-NO_2
	2.00 ppm	***CH_2***-CH_2-NO_2	p	
	4.37 ppm	***CH_2***-NO_2	t	
**D-ONO_2**	1.36 ppm	***CH_2***-CH_2-CH_2-ONO_2	m	D-ONO_2
	1.71 ppm	***CH_2***-CH_2-ONO_2	p	
	4.44 ppm	***CH_2***-ONO_2	t	
**D-$NHNO_2$**	1.38 ppm	***CH_2***-CH_2-CH_2-$NHNO_2$	m	D-$NHNO_2$
	1.76 ppm	***CH_2***-CH_2-$NHNO_2$	p	
	3.00 ppm	***CH_2***-$NHNO_2$	q	
	7.94 ppm	***$NHNO_2$***	broad s	

Table S4: Summary of new ^{1}H-NMR spectroscopy peaks seen in irradiated materials.

MOLECULE	NEW ^{1}H-NMR PEAK	NMR COUPLING	ASSIGNMENT
**300 kGy D-H**	2.00 ppm	m	dodecene (partial)
	4.93 ppm	d of sext	--
	5.00 ppm	d of quin	--
	5.39 ppm	m	--
	5.82 ppm	m	dodecene
**300 kGy D-N_3**	1.86 ppm	p	--
	2.33 ppm	t	nitrile
	3.53 ppm	t	--
	3.76 ppm	t	--
**300 kGy D-NO_2**	1.49 ppm	broad s	--
**300 kGy D-ONO_2**	2.35 ppm	t	aldehyde
	2.42 ppm	t	aldehyde
	3.64 ppm	t	alcohol
	9.76 ppm	s	aldehyde

Table S5: Summary of ^{1}H-NMR spectroscopy peaks unique to species identified in irradiated materials using GC-TOFMS.

MOLECULE	^{1}H-NMR PEAK	ASSIGNMENT	NMR COUPLING	GC-TOFMS IDENTIFIED
**Dodecyl amine**	1.19 ppm	***NH_2***	m	D-N_3
	2.68 ppm	***CH_2***-NH_2	m	
**Dodecene**	2.04 ppm	***CH_2***-CH=CH_2	m	D-H, D-N_3, D-NO_2
	4.94 ppm	***CH_2***	m	
	4.96 ppm	***CH_2***	m	
	5.81 ppm	***CH***=CH_2	m	
**Dodecanenitrile**	1.44 ppm	***CH_2***-CH_2-CH_2-C≡N	m	D-N_3
	1.65 ppm	***CH_2***-CH_2-C≡N	p	
	2.33 ppm	***CH_2***-C≡N	t	
**Octanol**	3.64 ppm	***OH***	t	--
**Dodecanol**	3.63 ppm	***OH***	t	D-ONO_2
**Octanal**	2.35 ppm<br>2.41 ppm	***CH_2***-CH=O	d of t	D-ONO_2
	9.76 ppm	***CH***=O	s	
**Decanal**	2.35 ppm<br>2.42 ppm	***CH_2***-CH=O	d of t	D-ONO_2
	9.76 ppm	***CH***=O	s	
**Dodecanal**	2.35 ppm	***CH_2***-CH=O	t	--
	2.42 ppm	***CH_2***-CH=O	t	
	9.76 ppm	***CH***=O	s	

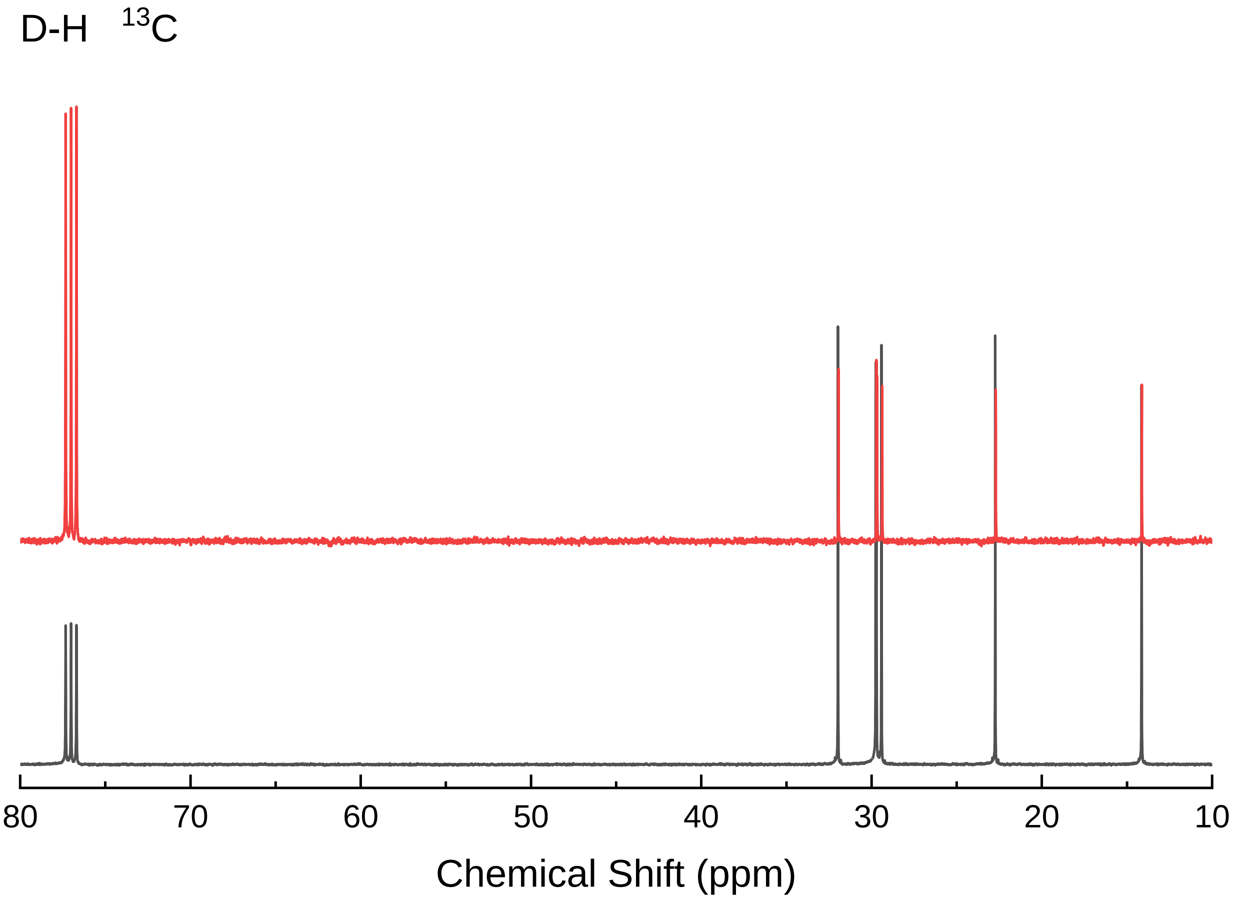

Figure S8: Control (bottom black line) and irradiated (top red line) ^{13}C-NMR spectra of D-H.

The ^{13}C-NMR spectrum for D-H showed the expected peaks at 14.26, 22.85, 29.54, 29.85, 29.88, and 32.10 ppm. Irradiation to 300 kGy-CaF_2 did not result in measurable changes.

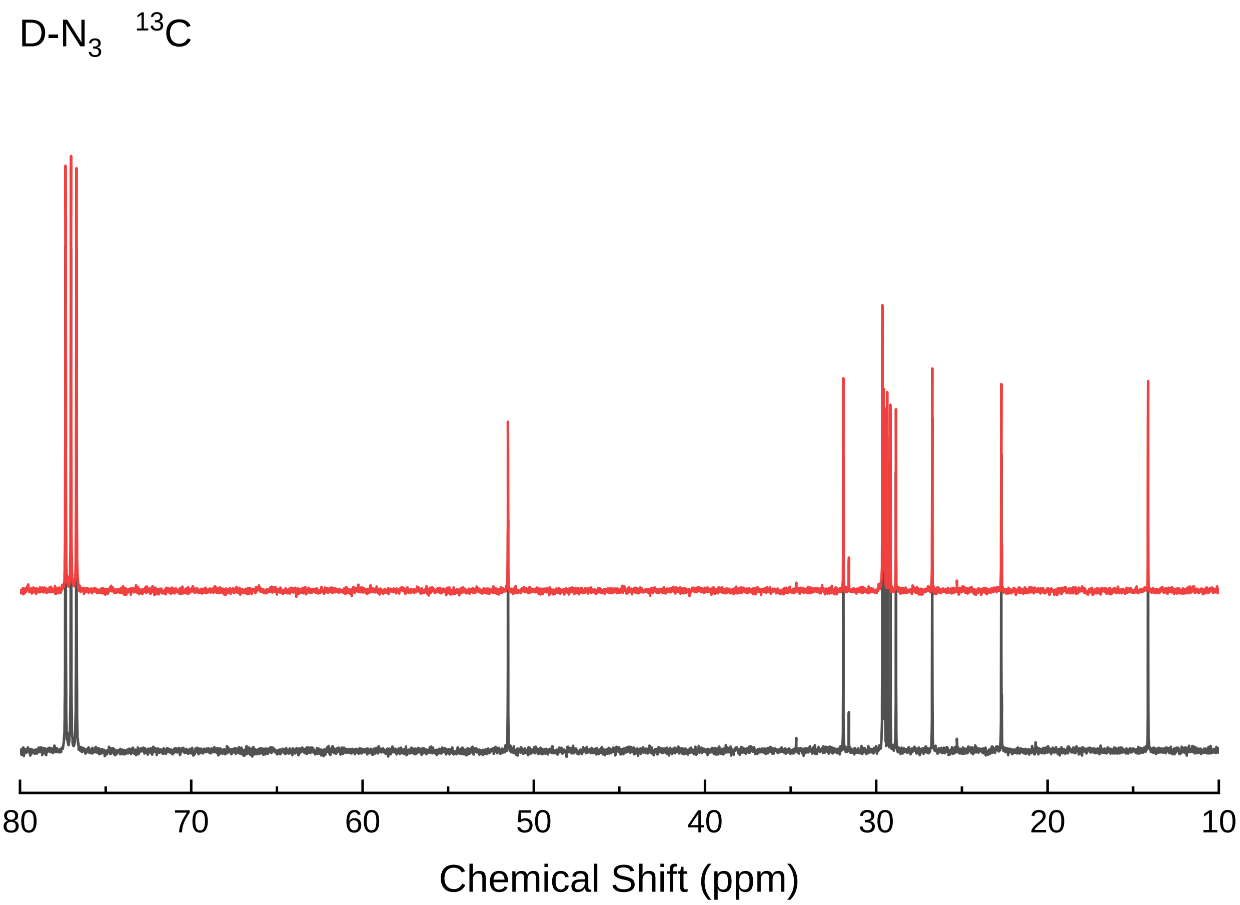

Figure S9: Control (bottom black line) and irradiated (top red line) ^{13}C-NMR spectra of D-N_3.

The ^{13}C-NMR spectrum for D-N_3 showed the expected peaks at 14.26, 22.84, 26.87, 28.99, 29.31, 29.49, 29.64, 29.70, 29.77, 30.06, and 51.64 ppm. Irradiation to 300 kGy-CaF_2 did not result in measurable changes.

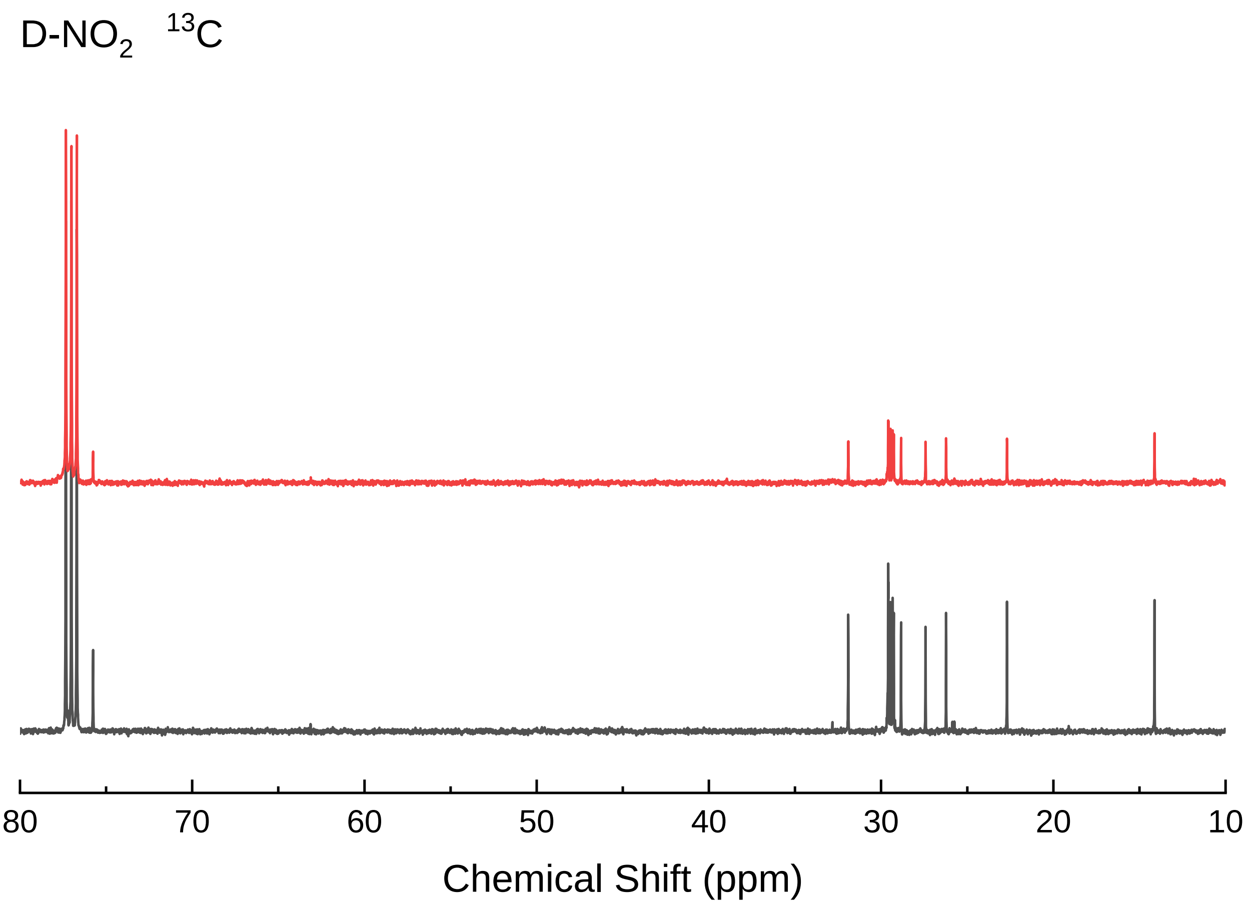

Figure S10: Control (bottom black line) and irradiated (top red line) ^{13}C-NMR spectra of D-NO_2.

The ^{13}C-NMR spectrum for D-NO_2 showed the expected peaks at 14.26, 22.83, 26.36, 27.56, 28.98, 29.40, 29.47, 29.59, 29.72, 29.73, 32.04, and 75.90 ppm. Irradiation to 300 kGy-CaF_2 did not result in measurable changes.

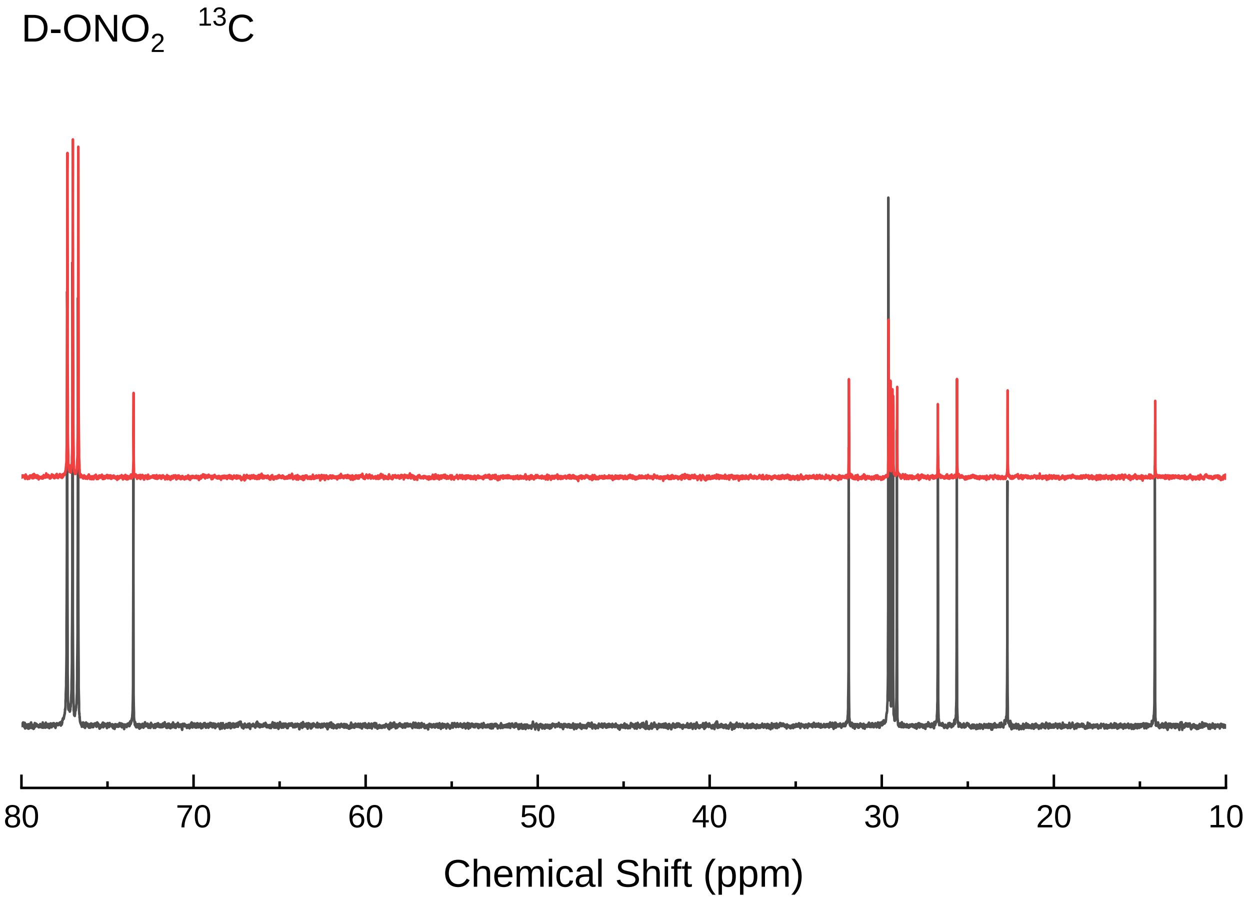

Figure S11: Control (bottom black line) and irradiated (top red line) ^{13}C-NMR spectra of D-ONO_2.

The ^{13}C-NMR spectrum for D-ONO_2 showed the expected peaks at 14.26, 22.84, 25.77, 26.87, 29.26, 29.48, 29.53, 29.64, 29.75, 32.06, and 73.63 ppm. Irradiation to 300 kGy-CaF_2 did not result in measurable changes.

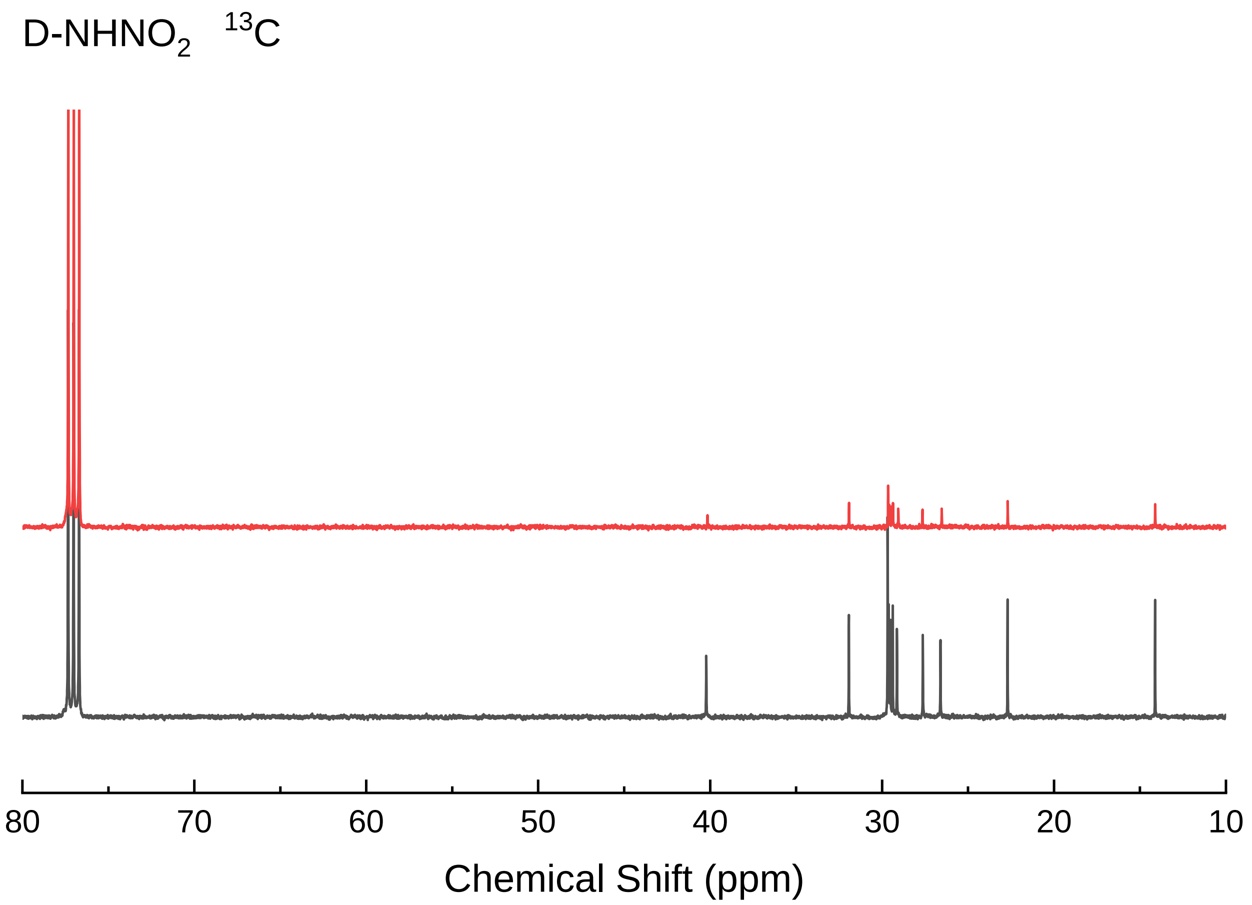

Figure S12: Control (bottom black line) and irradiated (top red line) ^{13}C-NMR spectra of D-NHNO$_2$.

The ^{13}C-NMR spectrum for D-NHNO$_2$ showed the expected peaks at 14.26, 22.84, 26.74, 29.27, 29.52, 29.66, 29.76, 29.81, 32.07, and 40.38 ppm. Irradiation to 300 kGy-CaF$_2$ did not result in measurable changes.

Attenuated Total Reflectance Fourier Transform Infrared (ATR-FTIR) spectroscopy

IR spectra were collected via Attenuated Total Reflectance Fourier Transform Infrared (ATR-FTIR) spectroscopy using a Nicolet iS50 spectrometer. This instrument was equipped with a diamond crystal and a KBr light filter. Each spectrum was the sum of 32 scans that ranged from 400 to 4000 cm^{-1}. The resolution of the spectra was 2 cm^{-1} with a background gain of 4.0. Happ-Genzel apodization was used with an aperture of 87 and an optical velocity of 0.4747. The ATR crystal was covered with a small amount of material sufficient for analysis, and the crystal was cleaned with technician grade acetone between each sample.

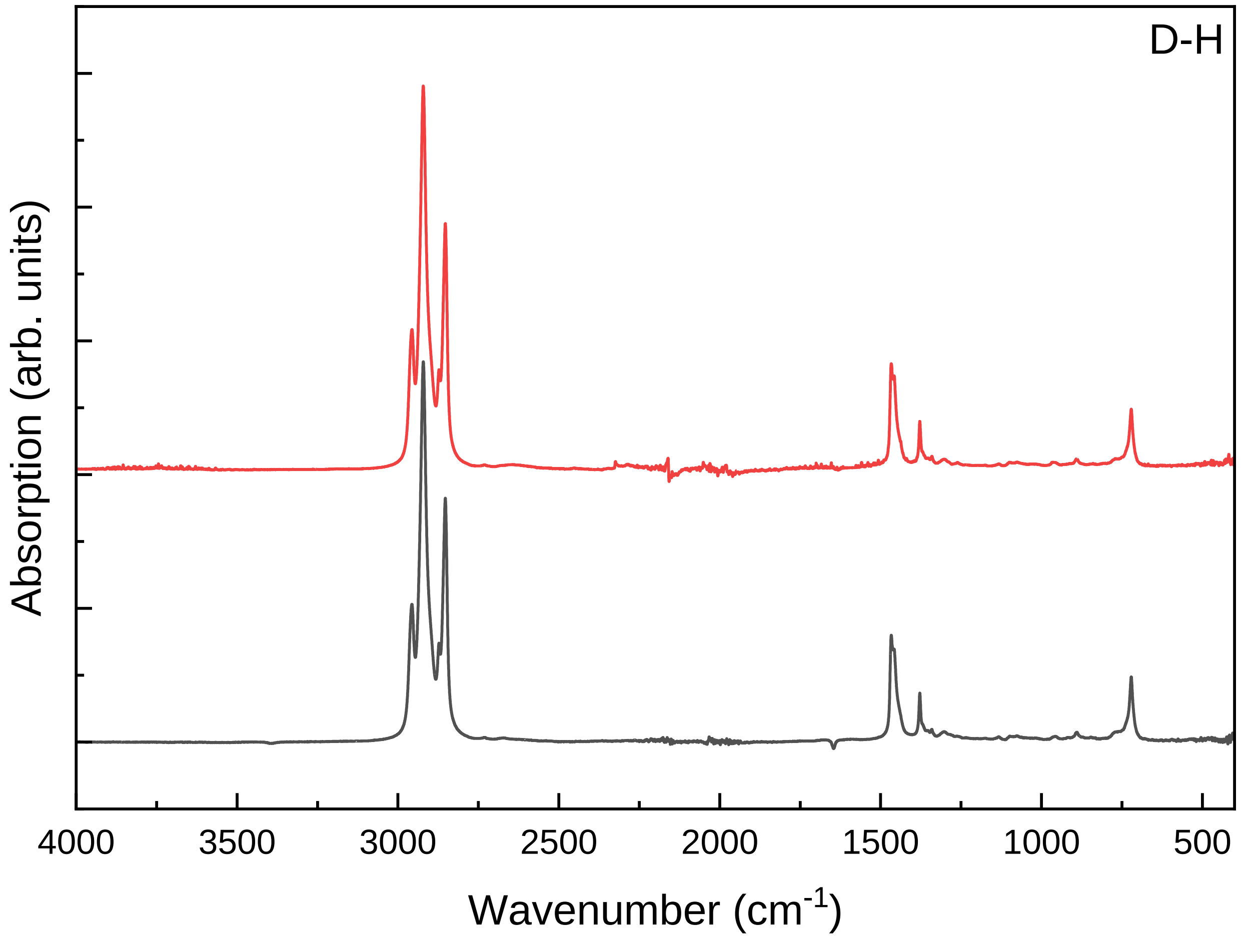

Figure S13: FTIR absorption spectra for control (bottom black line) and irradiated (top red line) D-H.

The FTIR spectra for both the control and irradiated dodecane (D-H) is found in Figure S12 above. All characteristic peaks for *n*-dodecane were present: 2957 (CH_3 asymmetric stretch), 2921 (CH_2 asymmetric stretch), 2873 (CH_3 symmetric stretch), 2853 (CH_2 symmetric stretch), 1466 (CH_2 symmetric deformation), 1458 (CH_3 symmetric deformation), 1378 (CH_2 wag), 889 (C-C-C angular deformation), and 721 (CH_2 rock).[2]

A dose of 300 kGy-CaF_2 gamma rays did not result in measurable changes in the FTIR spectrum.

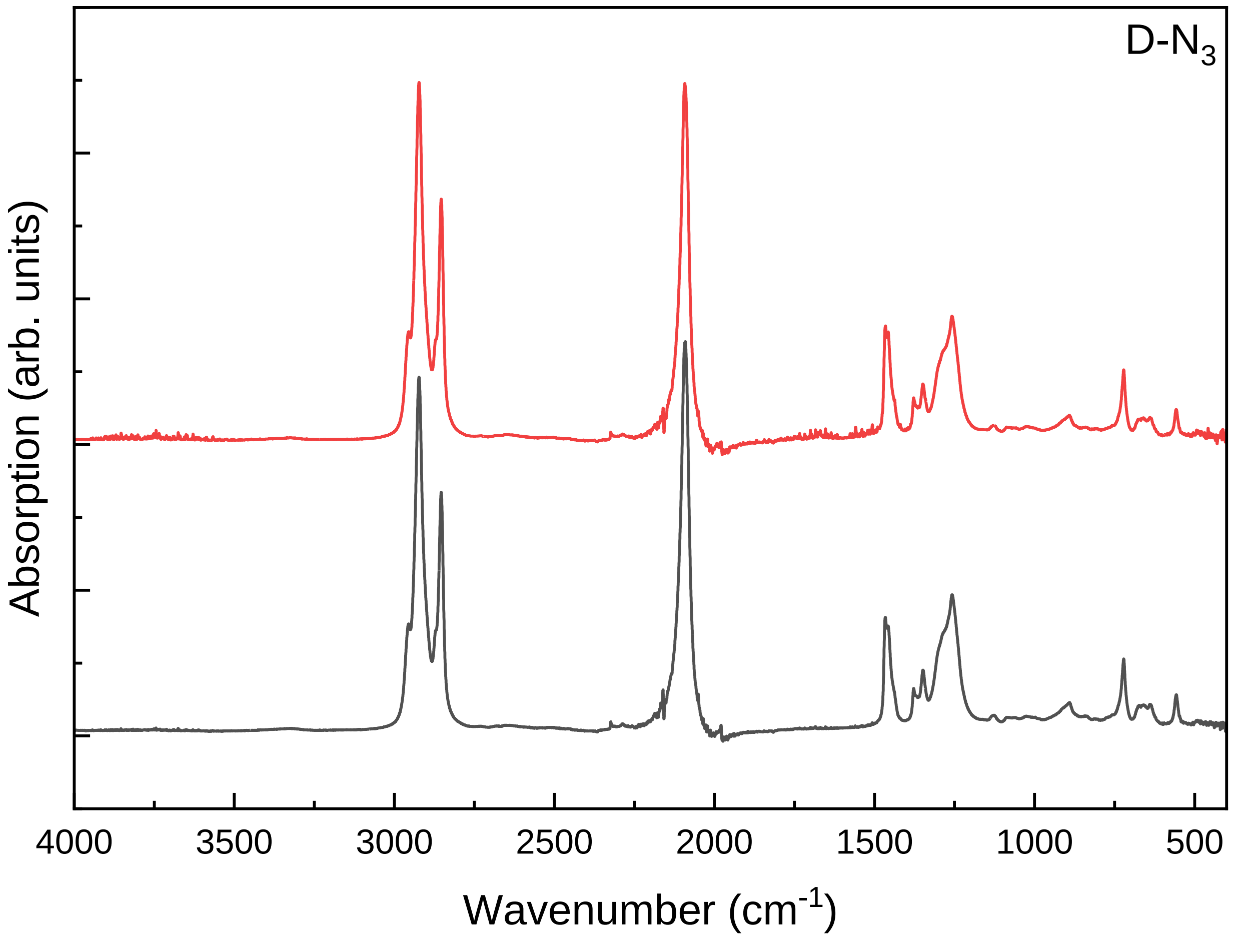

Figure S14: FTIR absorption spectra for control (bottom black line) and irradiated (top red line) D-N_3.

The FTIR spectra for both the control and irradiated dodecyl azide (D-N_3) is found in Figure S13 above. All characteristic peaks for *n*-dodecane were once again present: 2955 (CH_3 asymmetric stretch), 2923 (CH_2 asymmetric stretch), 2871 (CH_3 symmetric stretch), 2854 (CH_2 symmetric stretch), 1466 (CH_2 symmetric deformation), 1457 (CH_3 symmetric deformation), 1378 (CH_2 wag), 891 (C-C-C angular deformation), and 721 (CH_2 rock).[2] In addition to the *n*-dodecane peaks, several other peaks were present. A strong broad peak at 2091 cm^{-1} belonging to the N-N-N asymmetric stretch was the most dominant peak, followed by peaks at 1348, 1288, and 1258 cm^{-1}. The peak at 1258 cm^{-1} is likely the N-N-N symmetric stretch peak,[3,4] though the bands at 1348[5] and 1288[3] have also been classified as such.

A dose of 300 kGy-CaF_2 gamma rays did not result in measurable changes in the FTIR spectrum.

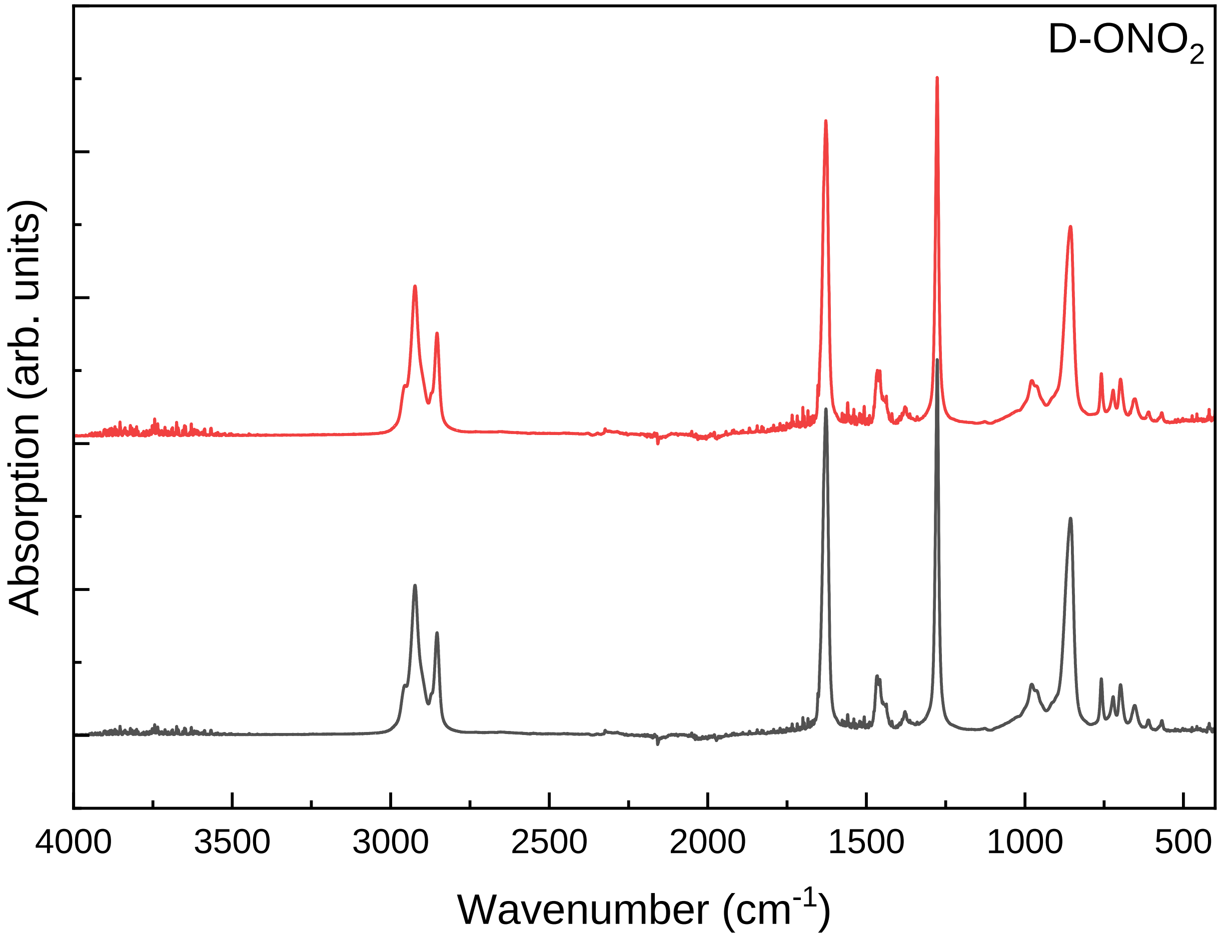

Figure S15: FTIR absorption spectra for control (bottom black line) and irradiated (top red line) D-ONO_2.

The FTIR spectra for both the control and irradiated dodecyl nitrate ester (D-ONO_2) is found in Figure S14 above. Almost all characteristic peaks for *n*-dodecane were once again present; the C-C-C angular deformation peak was masked by other, larger peaks: 2956 (CH_3 asymmetric stretch), 2923 (CH_2 asymmetric stretch), 2873 (CH_3 symmetric stretch), 2854 (CH_2 symmetric stretch), 1466 (CH_2 symmetric deformation), 1457 (CH_3 symmetric deformation), 1378 (CH_2 wag), and 722 (CH_2 rock).[2] In addition to the *n*-dodecane peaks, several new peaks were present. Two very large peaks were present at 1627 and 1276 cm^{-1}, belonging to the NO_2 asymmetric stretch and the CH_2 wag + NO_2 symmetric stretch, respectively.[6] A peak at 978 cm^{-1} belonging to the C-O stretch was also present, as well as a peak at 856 cm^{-1} belonging to the O-N stretch + NO_2 symmetric stretch and a peak at 759 cm^{-1} belonging to the ONO_2 out of plane bend.[6]

A dose of 300 kGy-CaF_2 gamma rays did not result in measurable changes in the FTIR spectrum.

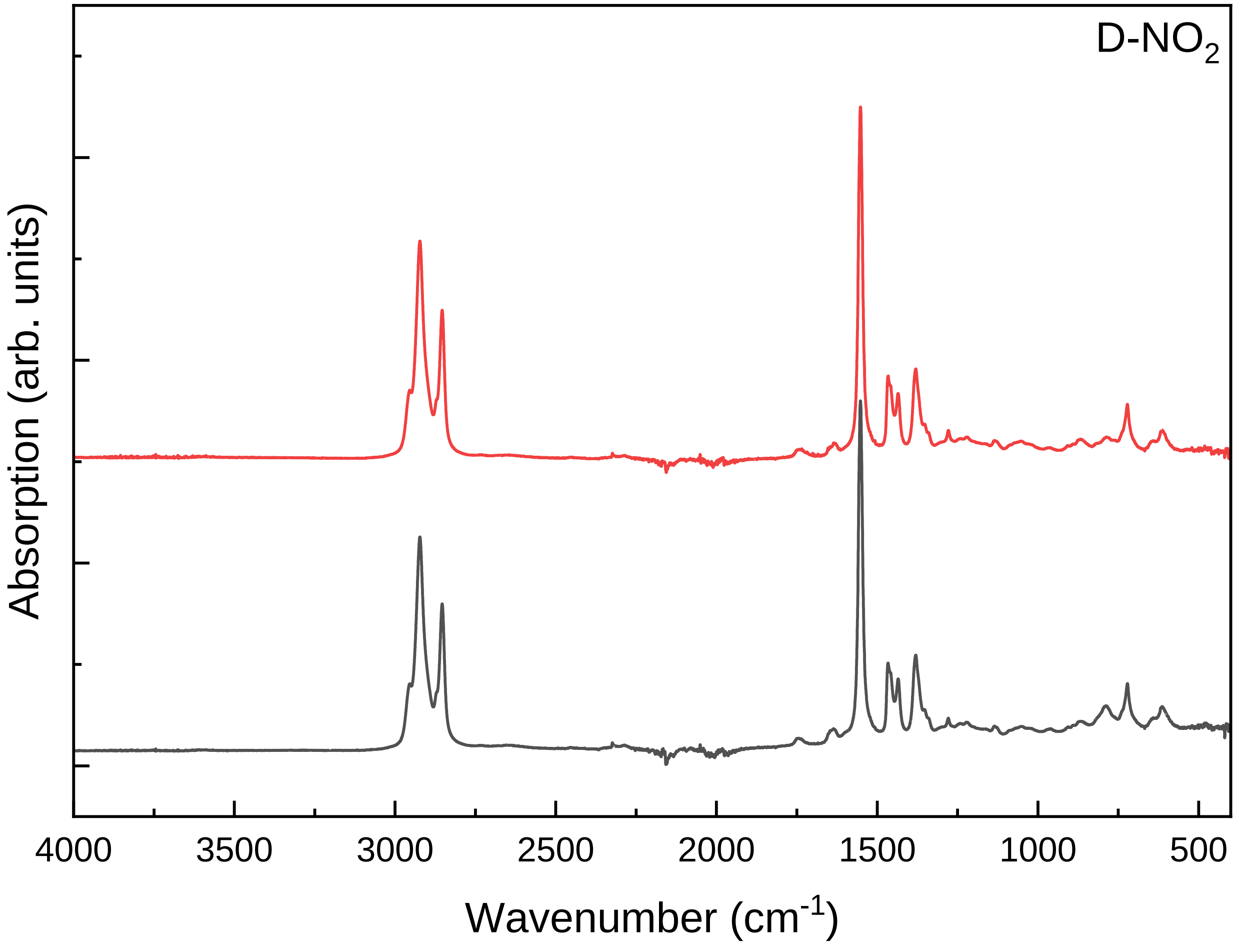

Figure S16: FTIR absorption spectra for control (bottom black line) and irradiated (top red line) D-NO_2.

The FTIR spectra for both the control and irradiated dodecane (D-H) is found in Figure S15 above. Almost all characteristic peaks for *n*-dodecane were once again present; the C-C-C angular deformation peak was not discernable: 2954 (CH_3 asymmetric stretch), 2923 (CH_2 asymmetric stretch), 2872 (CH_3 symmetric stretch), 2853 (CH_2 symmetric stretch), 1467 (CH_2 symmetric deformation), 1458 (CH_3 symmetric deformation), 1379 (CH_2 wag), and 722 (CH_2 rock).[2] In addition to the *n*-dodecane peaks, two new peaks were seen: a peak at 1552 cm^{-1} belonging to the NO_2 asymmetric stretch, and a peak at 1433 cm^{-1} belonging to the NO_2 symmetric stretch.[7]

A dose of 300 kGy-CaF_2 gamma rays did not result in measurable changes in the FTIR spectrum.

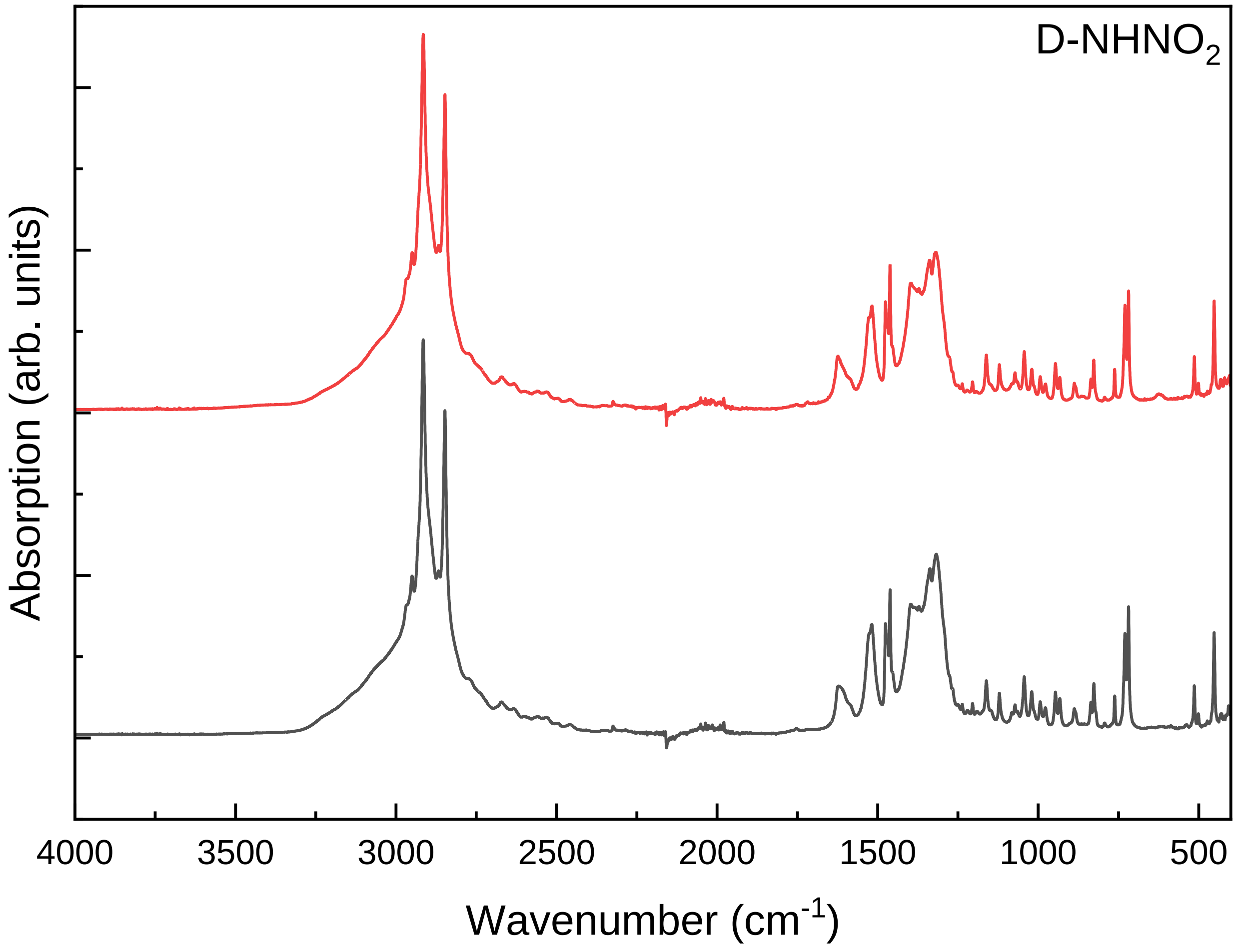

Figure S17: FTIR absorption spectra for control (bottom black line) and irradiated (top red line) D-NHNO$_2$.

The FTIR spectra for both the control and irradiated dodecane (D-H) is found in Figure S16 above. Most of the characteristic peaks for *n*-dodecane were present; the peaks below 1400 cm^{-1} were masked by the many peaks present in nitramine. The *n*-dodecane peaks were: 2950 (CH_3 asymmetric stretch), 2915 (CH_2 asymmetric stretch), 2868 (CH_3 symmetric stretch), 2848 (CH_2 symmetric stretch), 1469 (CH_2 symmetric deformation), and 1462 (CH_3 symmetric deformation).[2] In addition to the *n*-dodecane modes, there were several peaks in the ~1600-400 cm^{-1} region. Due to the complexity of the signal, published FTIR spectra do not make an attempt to index the pattern which makes comparison with literature quite difficult.

A dose of 300 kGy-CaF_2 gamma rays did not result in measurable changes in the FTIR spectrum.

Raman spectroscopy

Bulk bond analysis was completed using a combination of Raman and IR spectroscopies. Raman spectroscopy was achieved with a Thermo Scientific iS50 Raman spectrometer equipped with the CaF_2 beamsplitter. Each spectrum was the sum of 64 scans that ranged from 100 to 3700 cm^{-1}. The resolution of the spectra was 4 cm^{-1} and there was no background gain. Happ-Genzel apodization was used with an aperture of 75 and an optical velocity of 0.3165. The material was placed into an aluminum boat and the laser was focused onto the sample until a maximum signal strength was reached.

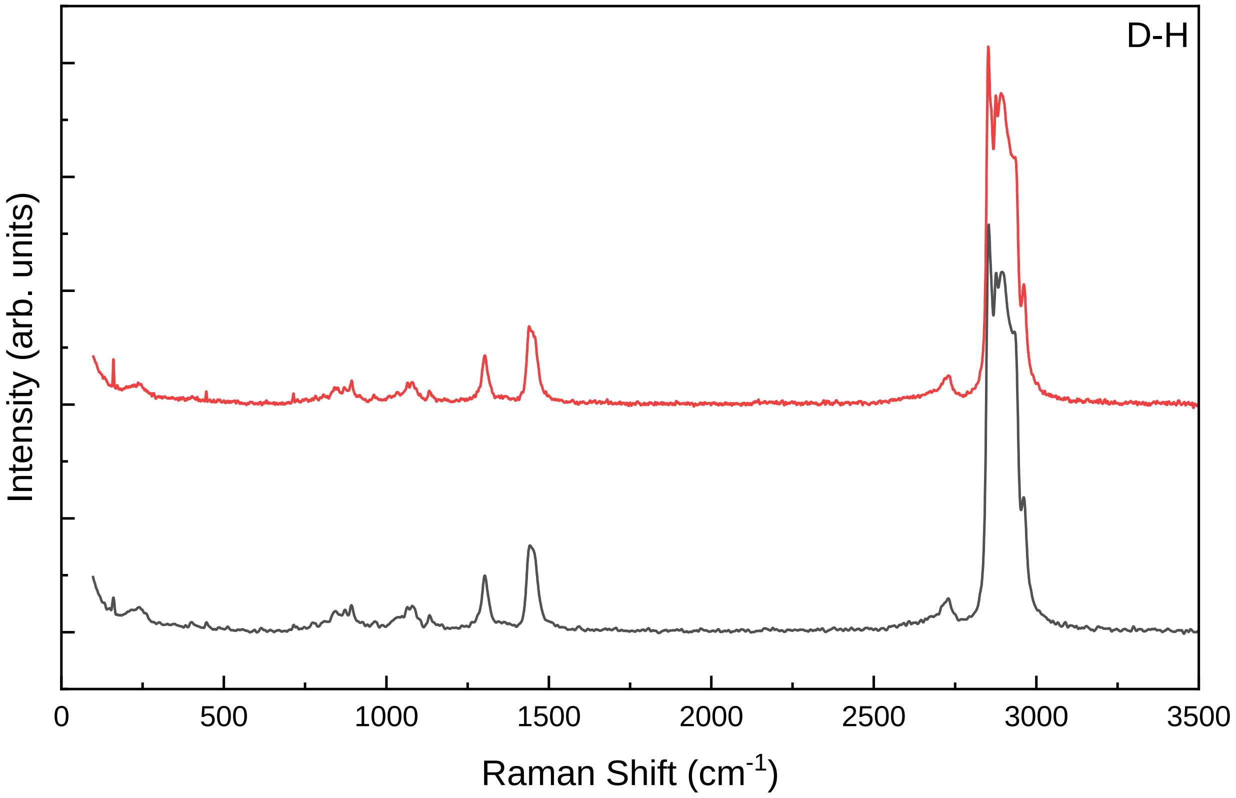

Figure S18: Raman spectra for control (bottom black line) and irradiated (top red line) D-H.

The Raman spectra for both the control and irradiated dodecane (D-H) is found in Figure S17 above. All characteristic peaks for *n*-dodecane were present. The C-H stretching modes (both CH_2 and CH_3 symmetric and asymmetric stretch) were found at 2961, 2934, 2896, 2876, and 2854 cm^{-1}.[8,9] The CH_2 and CH_3 scissor overtone was at 2729 cm^{-1},[9] the CH_3 bending modes were at 1451 and 1442 cm^{-1},[8] and the diamond first order mode was at 1303 cm^{-1}.[8] The C-C stretch modes were at 1133, 1081, and 1065 cm^{-1} while the CH_3 rocking mode was at 893 cm^{-1}.[8]

A dose of 300 kGy-CaF_2 gamma rays did not result in measurable changes in the Raman spectrum.

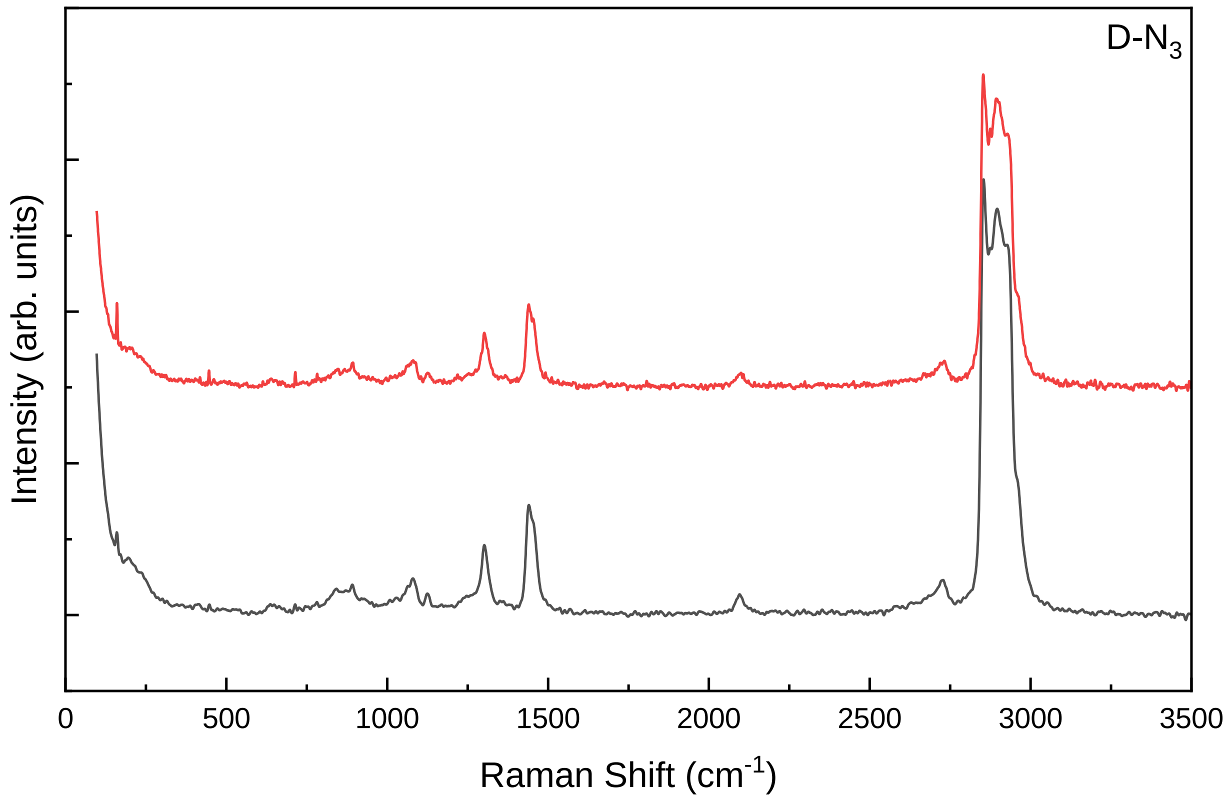

Figure S19: Raman spectra for control (bottom black line) and irradiated (top red line) D-N_3.

The Raman spectra for both the control and irradiated dodecyl azide (D-N_3) is found in Figure S18 above. All characteristic peaks for *n*-dodecane were present. The C-H stretching modes (both CH_2 and CH_3 symmetric and asymmetric stretch) were found at 2960, 2931, 2896, 2875, and 2854 cm^{-1}.[8,9] The CH_2 and CH_3 scissor overtone was at 2729 cm^{-1},[9] the CH_3 bending modes were at 1454 and 1440 cm^{-1},[8] and the diamond first order mode was at 1302 cm^{-1}.[8] The C-C stretch modes were at 1125, 1079, and 1066 cm^{-1} while the CH_3 rocking mode was at 892 cm^{-1}.[8] In addition to the *n*-dodecane modes, a peak at 2096 cm^{-1} belonging to the N_3 stretching mode[10,11] and a peak at 1244 cm^{-1} belonging to the bending N_3 mode[10] were seen. Interestingly, the ~1340 cm^{-1} peak characteristic of azides was not present.

A dose of 300 kGy-CaF_2 gamma rays did not result in measurable changes in the Raman spectrum.

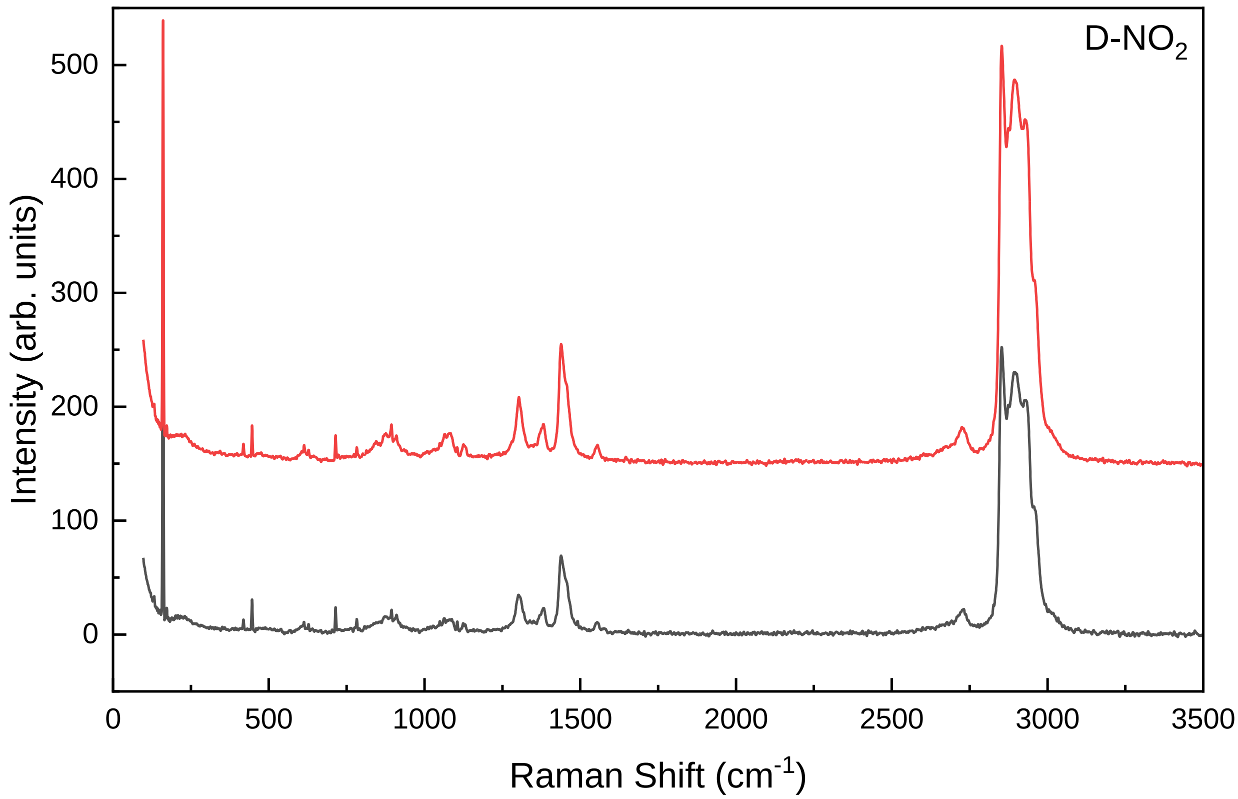

Figure S20: Raman spectra for control (bottom black line) and irradiated (top red line) D-NO_2.

The Raman spectra for both the control and irradiated dodecyl nitro (D-NO_2) is found in Figure S19 above. All characteristic peaks for *n*-dodecane were present. The C-H stretching modes (both CH_2 and CH_3 symmetric and asymmetric stretch) were found at 2962, 2934, 2897, 2875, and 2853 cm^{-1}.[8,9] The CH_2 and CH_3 scissor overtone was at 2730 cm^{-1},[9] the CH_3 bending modes were at 1457 and 1439 cm^{-1},[8] and the diamond first order mode was at 1303 cm^{-1}.[8] The C-C stretch modes were at 1126, 1082, and 1063 cm^{-1} while the CH_3 rocking mode was at 894 cm^{-1}.[8] In addition to the *n*-dodecane modes, the NO_2 asymmetric stretch was at 1555 cm^{-1}, the NO_2 symmetric stretch was at 1383 cm^{-1}, and the NO_2 scissor mode was at 783 cm^{-1}.[12,13]

A dose of 300 kGy-CaF_2 gamma rays did not result in measurable changes in the Raman spectrum.

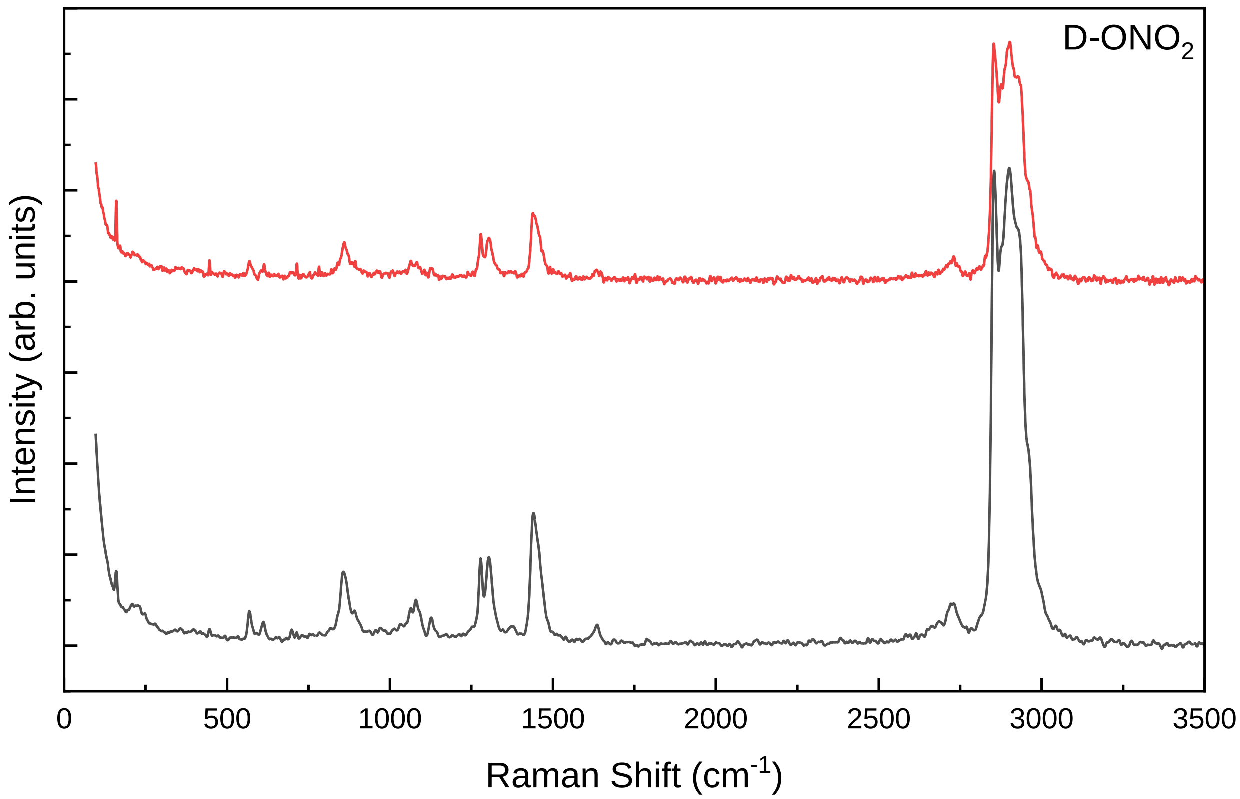

Figure S21: Raman spectra for control (bottom black line) and irradiated (top red line) D-ONO_2.

The Raman spectra for both the control and irradiated dodecyl nitrate ester (D-ONO_2) is found in Figure S20 above. All characteristic peaks for *n*-dodecane were present. The C-H stretching modes (both CH_2 and CH_3 symmetric and asymmetric stretch) were found at 2962, 2934, 2901, 2876, and 2854 cm^{-1}.[8,9] The CH_2 and CH_3 scissor overtone was at 2731 cm^{-1},[9] the CH_3 bending modes were at 1455 and 1441 cm^{-1},[8] and the diamond first order mode was at 1304 cm^{-1}.[8] The C-C stretch modes were at 1127, 1080, and 1064 cm^{-1} while the CH_3 rocking mode was at 892 cm^{-1}.[8] In addition to the *n*-dodecane modes, the NO_2 symmetric stretch was at 1278 cm^{-1} and the NO_2 asymmetric stretch was likely at 1450 cm^{-1}, though the asymmetric mode would be masked by the CH_3 bending modes.[12] Additionally, the O-N stretch was at 858 and the ONO_2 rock was at 611 cm^{-1}.[12]

A dose of 300 kGy-CaF_2 gamma rays did not result in measurable changes in the Raman spectrum.

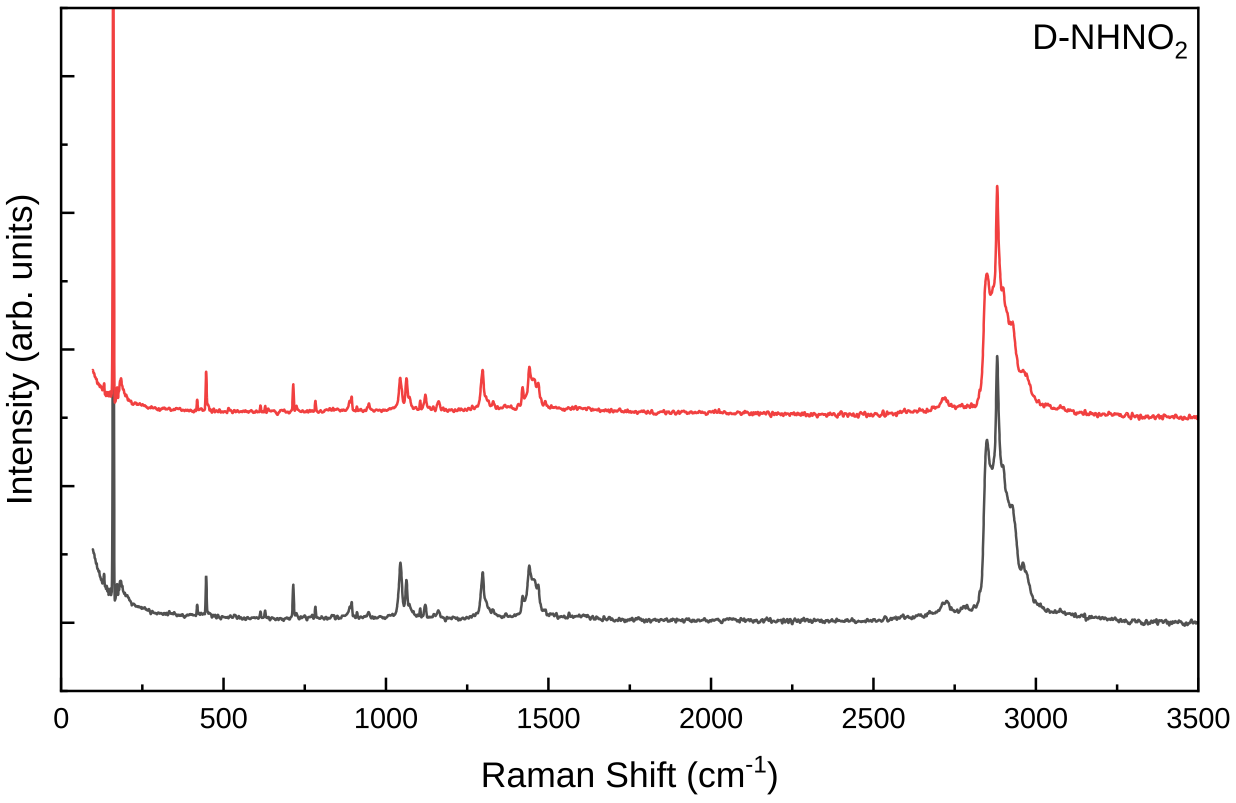

Figure S22: Raman spectra for control (bottom black line) and irradiated (top red line) D-NHNO2.

The Raman spectra for both the control and irradiated dodecyl nitramine (D-$NHNO_2$) is found in Figure S21 above. All characteristic peaks for *n*-dodecane were present. The C-H stretching modes (both CH_2 and CH_3 symmetric and asymmetric stretch) were found at 2960, 2927, 2899, 2881, and 2850 cm^{-1}.[8,9] The CH_2 and CH_3 scissor overtone was at 2725 cm^{-1},[9] the CH_3 bending modes were at 1456 and 1442 cm^{-1},[8] and the diamond first order mode was at 1297 cm^{-1}.[8] The C-C stretch modes were at 1122, 1062, and 1043 cm^{-1} while the CH_3 rocking mode was at 895 cm^{-1}.[8] In addition to the *n*-dodecane modes, there were multiple peaks in the ~600-1500 cm^{-1} range. Many of the published nitramine Raman spectra are based on ring structures and do not attempt to index each peak, making comparison with literature difficult for this material.

A dose of 300 kGy-CaF_2 gamma rays did not result in measurable changes in the Raman spectrum.

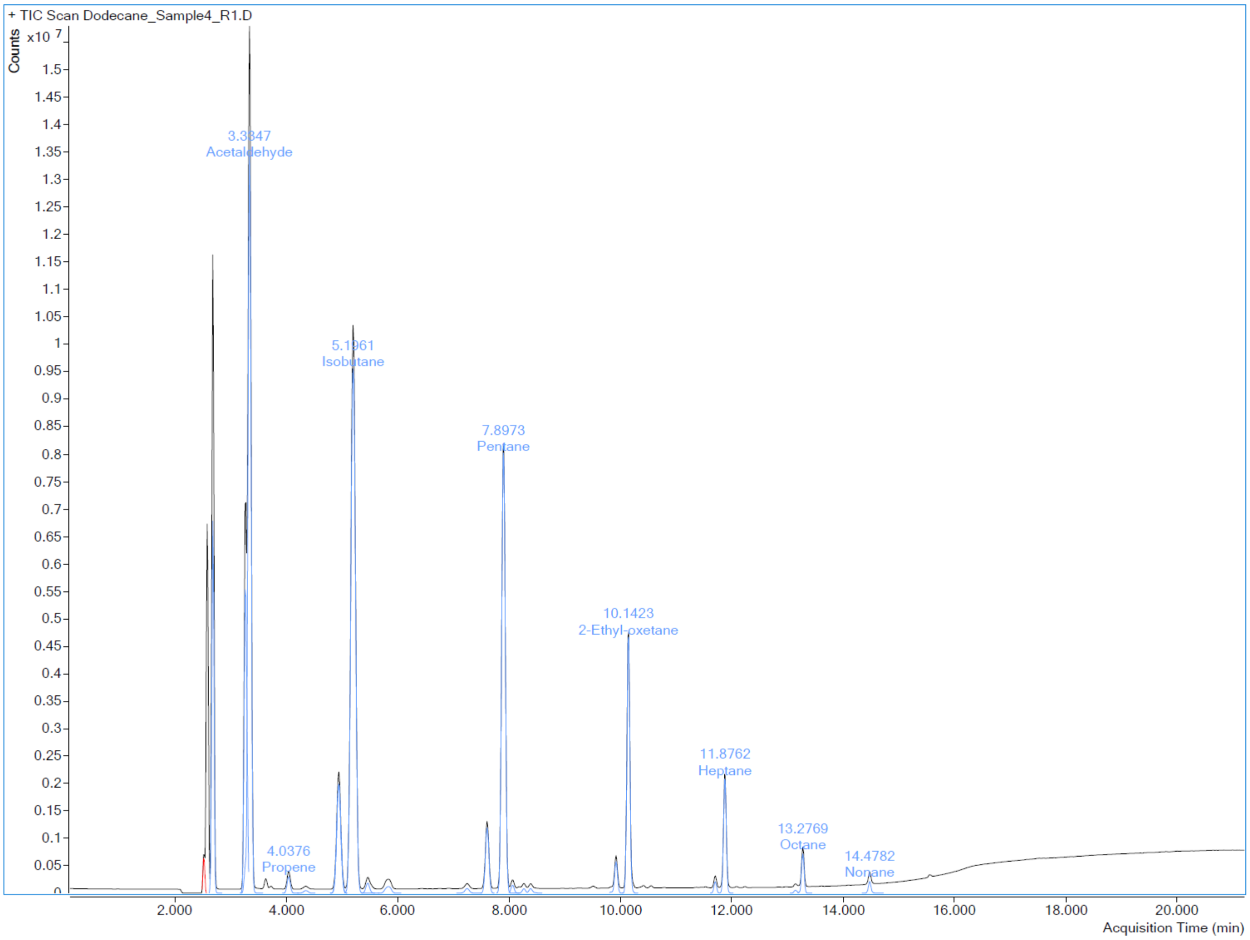

Figure S23: Total ion chromatograph (TIC) for the headspace gas of dodecane (D-H).

The TIC for the headspace gas of irradiated D-H is found above while the tabulated information is found in the table following. The only species displayed were those which had a library match factor of at least 80, so not all of the gas was able to be identified. The results show a mixture of alkanes and alkenes, as discussed in the main text.

Table S6: Quantitative H_2 and semi-quantitative unknowns for GC-MS of headspace gas in dodecane (D-H).

**Mol% H_2 in sample**	**64.22**					
**Compound Name**	**Match Factor**	**Base Peak Deconvoluted Area**	**Area %**	**Formula**	**Area mole fraction**	**Mol% of detected compound**
Nonane	99	139279	0.18	C_9H_{20}	0.00179	0.06
Octane	98	503202	0.65	C_8H_{18}	0.00647	0.23
Heptane	98	1376503	1.77	C_7H_{16}	0.01769	0.63
1-Heptene	98	110644	0.14	C_7H_{14}	0.00142	0.05
1-Hexene	98	373441	0.48	C_6H_{12}	0.00480	0.17
Pentane	98	11150155	14.33	C_5H_{12}	0.14331	5.13
2-Ethyl-oxetane	97	4108668	5.28	$C_5H_{10}O$	0.05281	1.89
1-Pentene	97	1352973	1.74	C_5H_{10}	0.01738	0.62
2-Methyl butane	96	103725	0.133	C_5H_{12}	0.00133	0.05
1,2-Diethyl-, cis-cyclobutane	94	26433	0.03	C_8H_{16}	0.00034	0.01
2-Pentene	94	208028	0.27	C_5H_{10}	0.00267	0.10
Isobutane	94	22146224	28.47	C_4H_{10}	0.28464	10.18
3-(1-Methylethyl)-oxetane	93	118361	0.15	$C_6H_{12}O$	0.00152	0.05
2-Methyl, 1-Propene	93	3772084	4.85	C_4H_8	0.04848	1.73
2-Butene	93	3506124	4.51	C_4H_8	0.04506	1.61
Propene	89	7153941	9.19	C_3H_6	0.09195	3.29

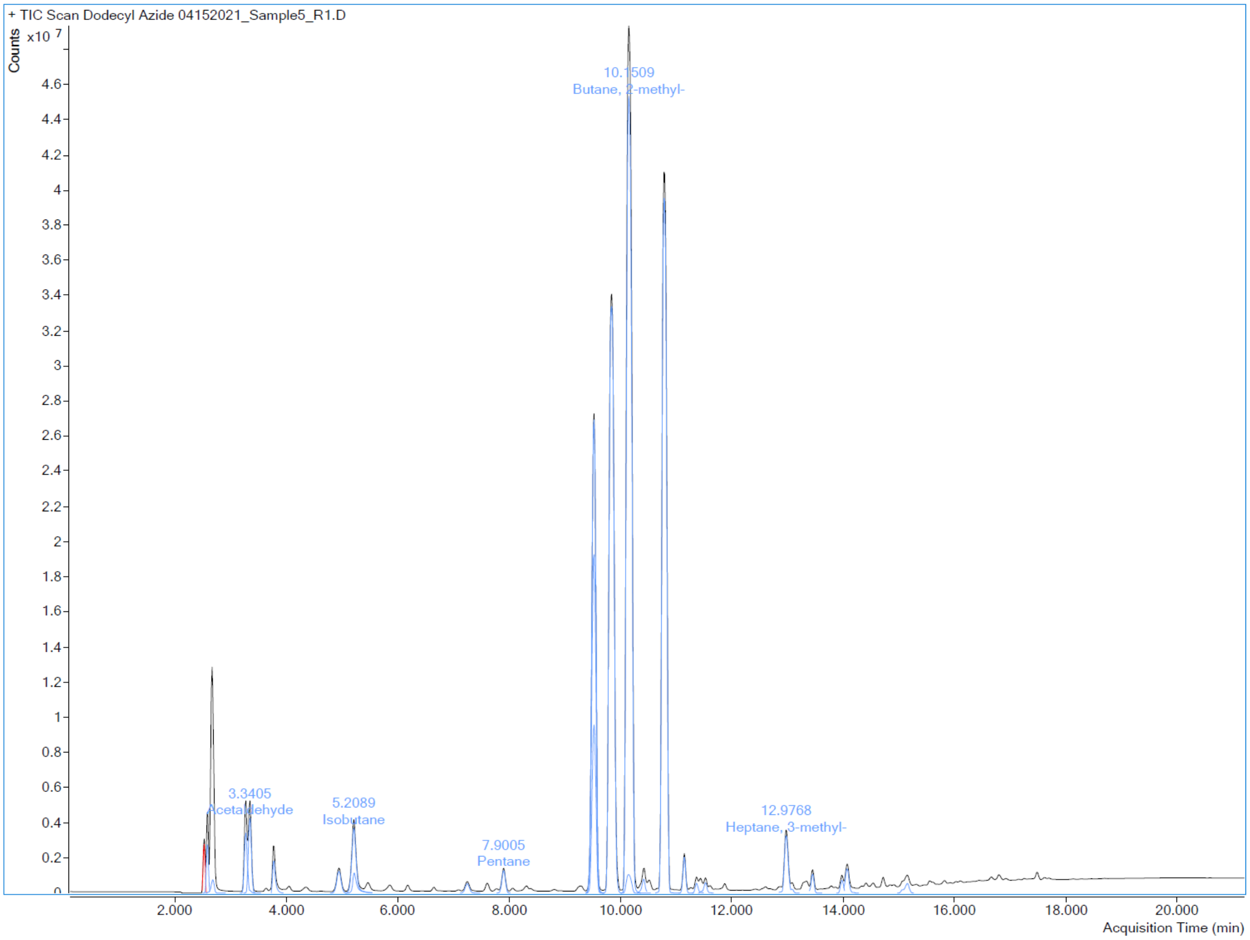

Figure S24: Total ion chromatograph (TIC) for the headspace gas of dodecyl azide (D-N_3).

The TIC for the headspace gas of irradiated D-N_3 is found above while the tabulated information is found in the table following. The only species displayed were those which had a library match factor of at least 80, so not all of the gas was able to be identified. The results show a mixture of alkanes and alkenes, as well as products which suggest a break in the trigger linkage, as discussed in the main text.

Table S7: Quantitative H_2 and semi-quantitative unknowns for GC-MS of headspace gas in dodecyl azide (D-N_3).

**Mol% H_2 in sample**	**3.89**					
**Compound Name**	**Match Factor**	**Base Peak Deconvoluted Area**	**Area %**	**Formula**	**Area mole fraction**	**Mol% of detected compound**
1-Methyl cyclopentene	98	2832585	1.08	C_6H_{10}	0.01085	1.04
3-Methyl pentane	98	42614283	16.32	C_6H_{14}	0.16318	15.68
2-Methyl pentane	98	31005233	11.87	C_6H_{14}	0.11873	11.41
Pentane	98	1774534	0.68	C_5H_{12}	0.00680	0.65
2,3-dimethyl heptane	97	1442881	0.55	C_9H_{20}	0.00553	0.53
3-Methyl hexane	97	555832	0.21	C_7H_{16}	0.00213	0.20
Methyl-cyclopentane	97	41389735	15.85	C_6H_{12}	0.15849	15.23
2-Methyl butane	97	570022	0.22	C_5H_{12}	0.00218	0.21
3-Ethyl-2-methyl hexane	96	641313	0.25	C_9H_{20}	0.00246	0.24
1-Ethyl-1-methyl cyclopentane	96	1121063	0.43	C_8H_{16}	0.00429	0.41
3-Methyl heptane	96	3316068	1.27	C_8H_{18}	0.01270	1.22
3,4,5-Trimethyl heptane	93	734376	0.27	$C_{10}H_{22}$	0.00281	0.27
2-Methyl 1-propene	92	2296408	0.88	C_4H_8	0.00879	0.85
2-Methyl butane	91	43797108	16.77	C_5H_{12}	0.16771	16.12
Isobutane	90	6289679	2.41	C_4H_{10}	0.02408	2.31
Acetylene	90	3336728	1.28	C_2H_2	0.01278	1.23
1-Hexene	89	795771	0.31	C_6H_{12}	0.00305	0.29
Propene	89	3735068	1.43	C_3H_6	0.01430	1.37
(2-Aziridinylethyl)amine	82	6304704	2.41	$C_4H_{10}N_2$	0.02414	2.32
Dimethylamine	81	3079337	1.18	C_2H_7N	0.01179	1.13

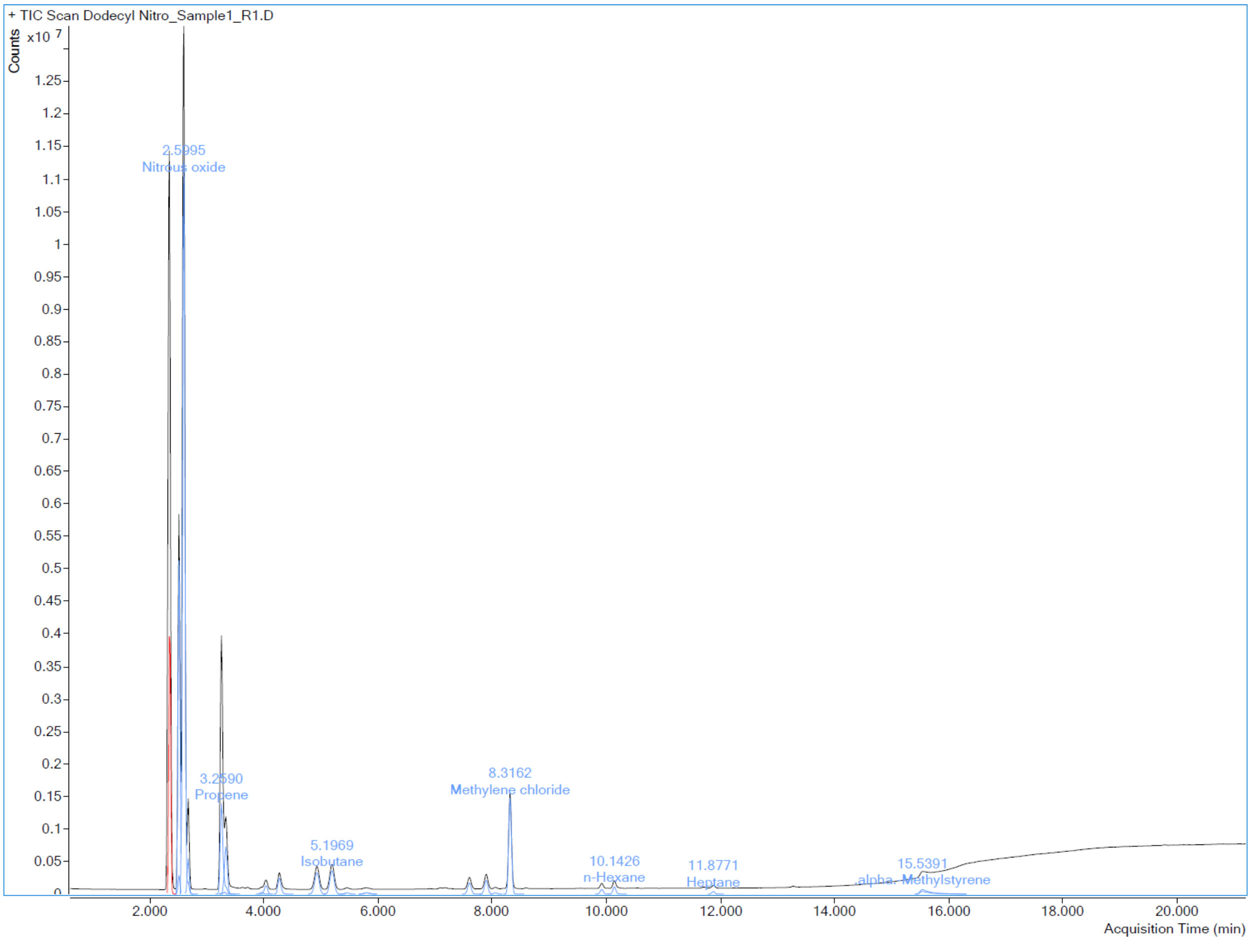

Figure S25: Total ion chromatograph (TIC) for the headspace gas of dodecyl nitro (D-NO_2).

The TIC for the headspace gas of irradiated D-NO_2 is found above while the tabulated information is found in the table following. The only species displayed were those which had a library match factor of at least 80, so not all of the gas was able to be identified. The results show a mixture of alkanes and alkenes, as well as products which suggest a break in the trigger linkage, as discussed in the main text.

Table S8: Quantitative H_2 and semi-quantitative unknowns for GC-MS of headspace gas in dodecyl nitro (D-NO_2).

**Mol% H_2 in sample**	**21.93**					
**Compound Name**	**Match Factor**	**Base Peak Deconvoluted Area**	**Area %**	**Formula**	**Area mole fraction**	**Mol% of detected compound**
Methylene chloride	99	1445372	3.46	CH_2Cl_2	0.03457	2.70
Pentane	98	295749	0.71	C_5H_{12}	0.00707	0.55
Chloromethane	98	59657	0.14	CH_3Cl	0.00143	0.11
Heptane	97	27656	0.07	C_7H_{16}	0.00066	0.41
1-Pentene	97	176981	0.42	C_5H_{10}	0.00423	0.33
Acetaldehyde	97	335438	0.80	C_2H_4O	0.00802	0.63
n-Hexane	96	94614	0.23	C_6H_{14}	0.00226	0.18
2-Hexene	95	47299	0.11	C_6H_{12}	0.00113	0.09
Isobutane	94	807361	1.93	C_4H_{10}	0.01931	1.51
Methylstyrene	92	219109	0.52	C_9H_{10}	0.00524	0.41
2-Methyl-1-butene	92	30010	0.07	C_5H_{10}	0.00072	0.06
2-Methyl-1-propene	92	615316	1.47	C_4H_8	0.01472	1.15
Nitrous oxide	91	20693882	49.50	N_2O	0.49504	38.65
Propene	89	2151568	5.15	C_3H_6	0.05105	4.02
2-Butene	88	36235	0.09	C_4H_8	0.00087	0.07
(2-Aziridinylethyl)amine	83	12627167	30.21	$C_4H_{10}N_2$	0.30207	23.58

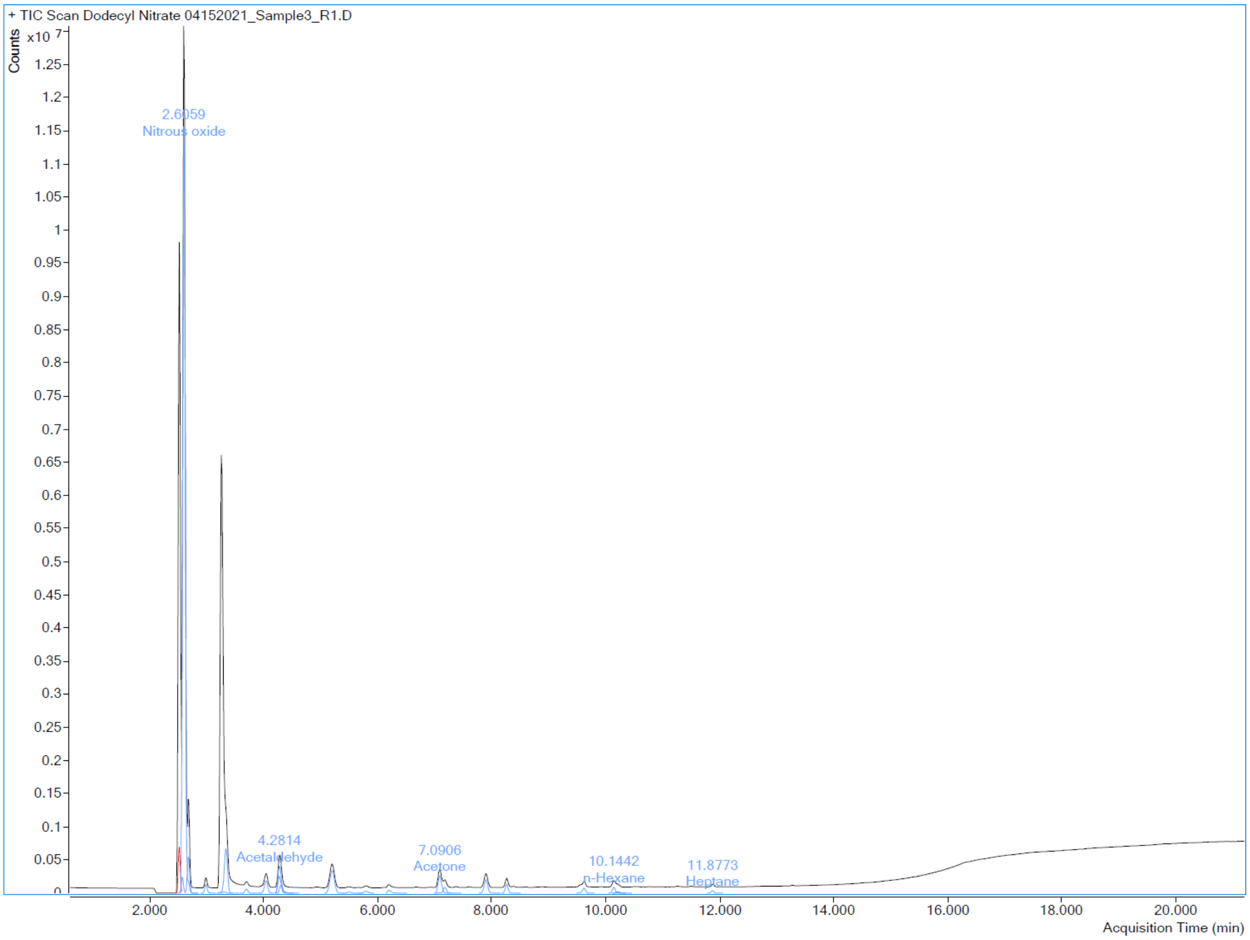

Figure S26: Total ion chromatograph (TIC) for the headspace gas of dodecyl nitrate ester (D-ONO_2).

The TIC for the headspace gas of irradiated D-ONO_2 is found above while the tabulated information is found in the table following. The only species displayed were those which had a library match factor of at least 80, so not all of the gas was able to be identified. The results show a mixture of alkanes and alkenes, as well as products which suggest a break in the trigger linkage, as discussed in the main text.

Table S9: Quantitative H_2 and semi-quantitative unknowns for GC-MS of headspace gas in dodecyl nitrate ester (D-ONO_2).

**Mol% H_2 in sample**	**2.62**					
**Compound Name**	**Match Factor**	**Base Peak Deconvoluted Area**	**Area %**	**Formula**	**Area mole fraction**	**Mol% of detected compound**
Pentane	98	279837	0.91	C_5H_{12}	0.00908	0.88
Methyl nitrate	97	342925	1.11	CH_3NO_3	0.01112	1.08
Acetone	97	690373	2.24	C_3H_6O	0.00239	2.18
Ethanol	97	74086	0.24	C_2H_6O	0.00240	0.23
Acetaldehyde	97	634921	2.06	C_2H_4O	0.02059	2.01
Trichloromethane	96	29420	0.10	$CHCl_3$	0.00095	0.09
n-Hexane	95	77779	0.25	C_6H_{14}	0.00252	0.25
Methyl formate	95	24693	0.08	$C_2H_4O_2$	0.00080	0.08
Isobutane	94	766791	2.49	C_4H_{10}	0.02487	2.42
Acetylene	94	362100	1.17	C_2H_2	0.01174	1.14
2-Butanone	92	169239	0.55	C_4H_8O	0.00549	0.53
Nitrous oxide	91	23604383	76.56	N_2O	0.76559	74.56
Heptane	90	19992	0.06	C_7H_{16}	0.00065	0.06
Methyl nitrite	90	140891	0.46	CH_3NO_2	0.00457	0.45
2-Butene	89	37788	0.12	C_4H_8	0.00123	0.12
Propene	89	235927	0.77	C_3H_6	0.00765	0.75
Dimethyl ether	88	769206	2.49	C_2H_6O	0.02495	2.43
Acetaldehyde	87	779440	2.53	C_2H_4O	0.02528	2.46
Formaldehyde	83	213501	0.69	CH_2O	0.00692	0.67
Propanal	81	123011	0.40	C_3H_6O	0.00399	0.39
Methyl alcohol	81	260557	0.85	CH_4O	0.00845	0.82

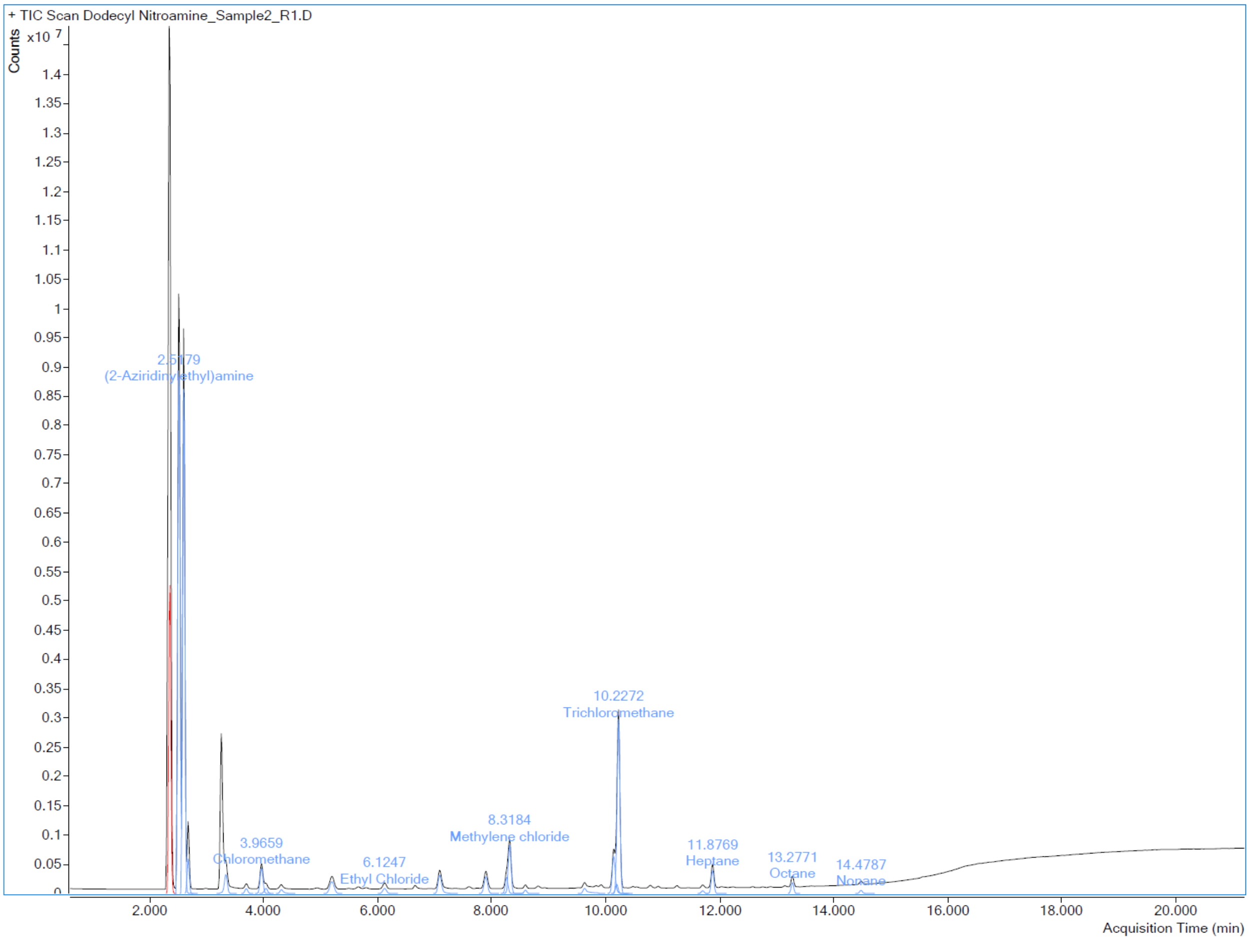

Figure S27: Total ion chromatograph (TIC) for the headspace gas of dodecyl nitramine (D-$NHNO_2$).

The TIC for the headspace gas of irradiated D-$NHNO_2$ is found above while the tabulated information is found in the table following. The only species displayed were those which had a library match factor of at least 80, so not all of the gas was able to be identified. The results show a mixture of alkanes and alkenes, as well as products which suggest a break in the trigger linkage, as discussed in the main text.

Table S10: Quantitative H_2 and semi-quantitative unknowns for GC-MS of headspace gas in dodecyl nitramine (D-$NHNO_2$).

**Mol% H_2 in sample**	**1.94**					
**Compound Name**	**Match Factor**	**Base Peak Deconvoluted Area**	**Area %**	**Formula**	**Area mole fraction**	**Mol% of detected compound**
Trichloromethane	100	4208042	6.59	$CHCl_3$	0.06588	6.46
Chloromethane	100	915938	1.43	CH_3Cl	0.01434	1.41
Methylene chloride	99	816112	1.28	CH_2Cl_2	0.01278	1.25
Oxygen	99	12057864	18.88	O_2	0.18876	18.51
Octane	98	130142	0.20	C_8H_{18}	0.00204	0.20
Acetone	98	827681	1.30	C_3H_6O	0.01296	1.27
Pentane	97	389154	0.61	C_5H_{12}	0.00609	0.60
Methyl alcohol	97	106222	0.17	CH_4O	0.00166	0.16
Nonane	96	35045	0.05	C_9H_{20}	0.00055	0.05
n-Hexane	96	571517	0.89	C_6H_{14}	0.00895	0.88
2-Butanone	96	296685	0.46	C_4H_8O	0.00464	0.46
Methyl nitrate	96	696725	1.09	CH_3NO_3	0.01091	1.07
Ethyl chloride	96	116206	0.18	C_2H_5Cl	0.00182	0.18
Heptane	95	233233	0.37	C_7H_{16}	0.00365	0.36
Nitro methane	95	64659	0.10	CH_3NO_2	0.00101	0.10
1-Heptene	94	25614	0.04	C_7H_{14}	0.00040	0.04
Isobutane	94	453897	0.71	C_4H_{10}	0.00711	0.70
Nitrous oxide	91	16618783	26.02	N_2O	0.26016	25.51
Propene	89	106274	0.17	C_3H_6	0.00166	0.16
(2-Aziridinylethyl)amine	83	23828623	37.30	$C_4H_{10}N_2$	0.37303	36.58

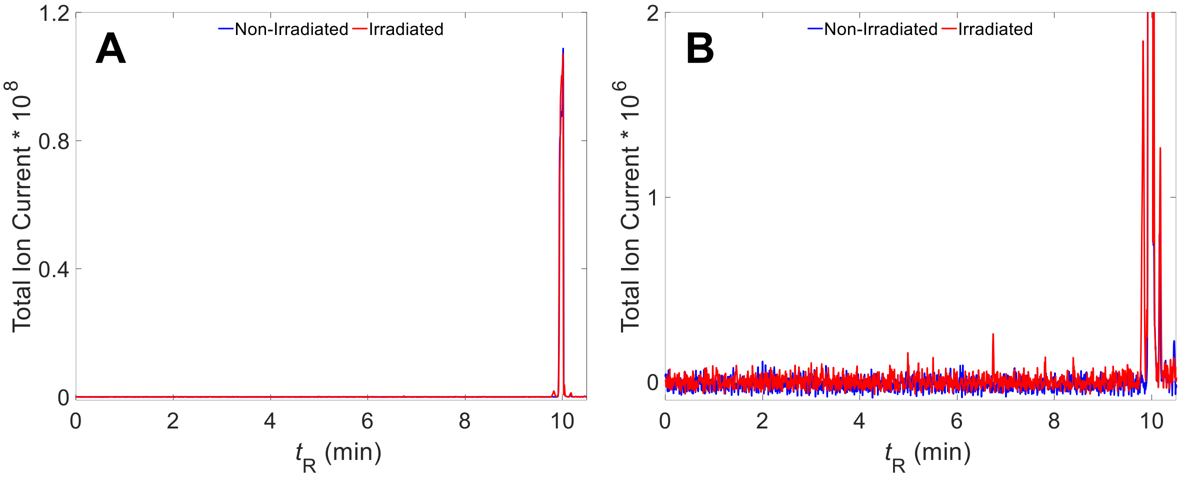

Figure S28: (**A**) GC-TOFMS TIC chromatograms for analysis of D-H. (**B**) Zoom-in of A.

The TIC chromatograms for GC-TOFMS analysis of D-H can be found in Figure S27. Minimal chemical changes were observed in the TIC for D-H.

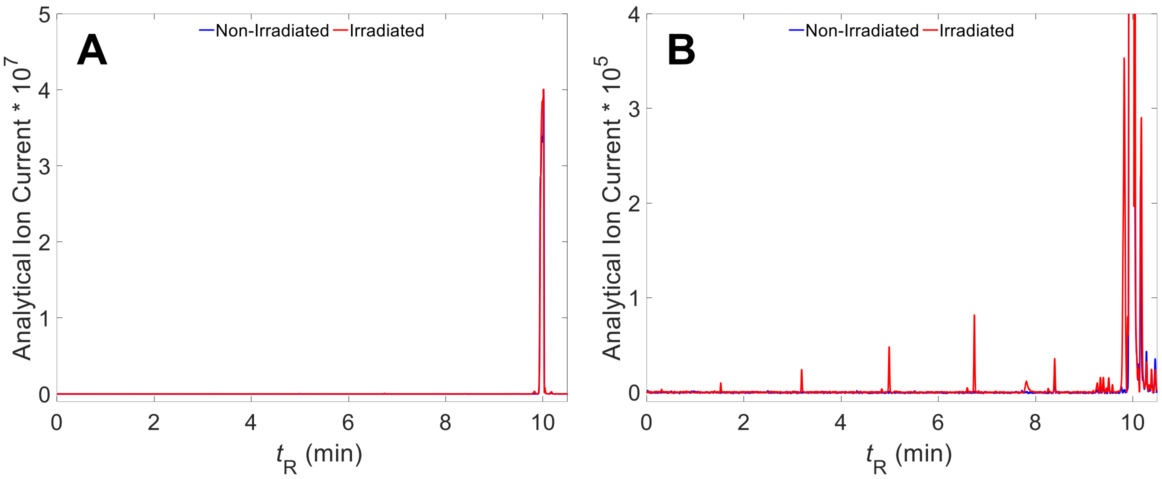

Figure S29: (**A**) GC-TOFMS AIC (m/z 41 and 43) chromatograms for analysis of dodecane. (**B**) Zoom-in of A.

The AIC chromatograms for GC-TOFMS analysis of D-H can be found in Figure S28. Numerous minor chemical changes were observed but dodecene was the only hit identified as shown in Table S10.

Table S11: Fisher ratio results for the comparison of the irradiated and non-irradiated D-H.

Hit #	Avg F-Ratio	Tile #	Grid #	t_R (min)	m/z with max F-ratio	Tentative Identification	Match Value
1	4988.6	148	1	9.83	111	Dodecene	881
2	1210.1	102	1	6.75	85		
3	460.0	75	2	4.99	99		
4	234.7	141	1	9.34	69		
5	213.9	153	1	10.19	168		
6	211.5	144	2	9.59	69		
7	133.3	48	2	3.19	43		
8	124.7	99	2	6.60	70		
9	101.7	140	1	9.28	57		
10	71.6	124	2	8.27	69		
11	63.1	142	2	9.46	70		
12	62.7	73	1	4.84	41		
13	58.0	141	2	9.39	69		
14	55.1	23	2	1.53	43		
15	51.5	151	1	10.06	168		
16	44.5	116	1	7.73	71		
17	40.7	156	1	10.39	70		
18	30.7	5	1	0.31	57		
19	29.9	149	1	9.90	168		
20	22.3	154	2	10.30	71		
21	20.1	138	1	9.16	73		
22	19.5	100	2	6.66	55		
23	17.8	146	2	9.77	55		
24	13.4	127	2	8.45	71		
25	12.4	126	2	8.40	70		

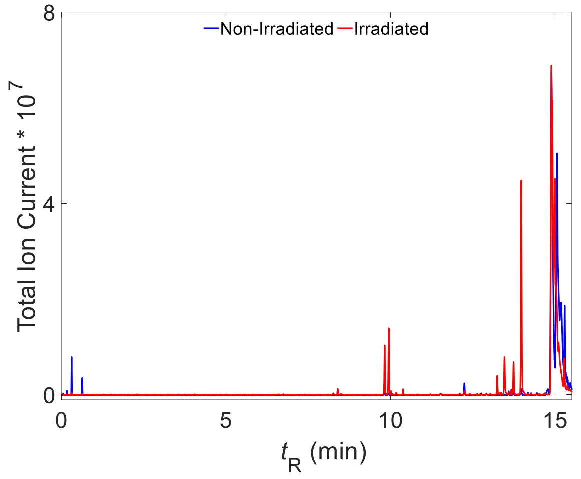

Figure S30: (**A**) GC-TOFMS TIC chromatograms for analysis of D-N_3.

The TIC chromatogram for GC-TOFMS analysis of D-N_3 can be found in Figure S29. Numerous chemical changes were observed in the TIC as discussed in the main text.

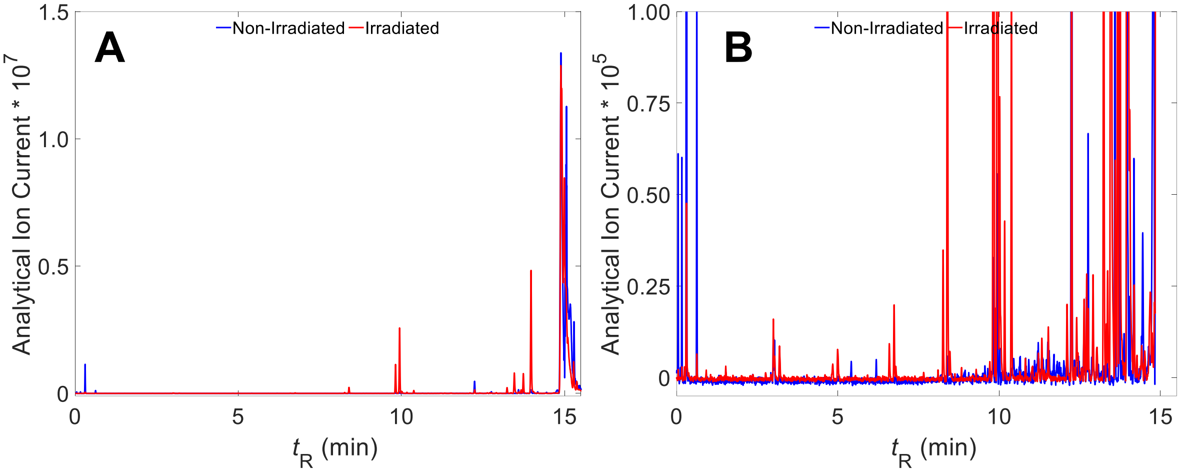

Figure S31: (**A**) GC-TOFMS AIC (m/z 55 and 43) chromatograms for analysis of D-N_3. (**B**) Zoom-in of A showing the numerous small level concentration compounds.

The AIC chromatograms for GC-TOFMS analysis of D-N_3 can be found in Figure S30. Numerous chemical changes were observed and many were able to be identified, as discussed in the main text and highlighted in Table S11.

Table S12: Fisher ratio results for the comparison of the irradiated and non-irradiated D-N_3.

**Hit #**	**F-Ratio**	**Tile #**	**Grid #**	t_R **(min)**	**m/z with max F-ratio**	**Tentative Identification**	**Match Value**
1	1489.3	150	1	9.95	86	Dodecanenitrile	881
2	888.6	206	1	13.74	77	1-azido-octane	822
4	666.1	124	2	8.27	69		
5	437.7	209	2	13.97	44	1-Decene	881
6	418.7	220	2	14.70	67		
7	352.8	126	2	8.40	84	Octanenitrile	840
8	310.9	220	1	14.67	55		
9	273.7	151	2	10.04	67		
10	237.8	200	2	13.36	70		
11	182.9	200	1	13.33	96	Dodecene	801
12	171.5	211	2	14.05	136		
13	169.8	99	2	6.60	69		
14	167.4	205	1	13.67	99	Decanenitrile	757
15	157.5	127	2	8.48	69		
16	147.0	202	1	13.46	41		
17	141.6	190	1	12.64	70		
18	141.1	188	2	12.56	70		
19	141.0	75	2	5.00	85		
20	137.2	214	2	14.28	69	Undecanenitrile	924
21	135.0	218	1	14.52	58		
22	120.7	102	1	6.75	85		
23	119.8	186	2	12.44	58		
24	118.4	14	2	0.92	67		
25	115.5	148	1	9.83	62	Octanenitrile	840
26	110.1	146	1	9.72	82	Nonanenitrile	881
27	103.5	204	1	13.60	82		
28	95.4	182	1	12.11	59		
29	83.5	156	2	10.38	98		
30	81.3	223	2	14.87	135		
31	78.3	221	2	14.74	138		
32	73.0	192	1	12.76	59		
33	72.0	199	1	13.24	98		
34	66.7	212	2	14.11	81		
35	63.7	231	2	15.42	46		
36	63.1	166	1	11.03	58		
37	57.3	213	1	14.14	138	Dodecamine	916
38	56.3	213	2	14.19	138		

39	56.0	231	1	15.38	61		
40	54.7	170	1	11.33	85		
41	54.6	48	2	3.20	85		
42	51.7	169	1	11.25	72		
43	49.9	133	1	8.84	69		
44	45.4	207	2	13.80	122		
45	44.9	173	1	11.48	85		
46	44.2	73	1	4.84	69		
47	40.5	203	2	13.51	100		
48	40.2	195	2	13.02	84		
49	33.6	216	2	14.43	54		
50	32.0	191	1	12.72	71		
51	30.2	186	1	12.39	91		
52	29.8	173	2	11.53	70		
53	29.0	178	1	11.85	82		
54	28.7	192	2	12.79	45		
55	27.9	168	2	11.22	96		
56	27.1	171	2	11.41	45		
57	25.7	168	1	11.14	113		
58	25.5	209	1	13.90	122		
59	23.5	153	2	10.17	79		
60	22.4	215	2	14.31	80		
61	22.0	224	2	14.93	52		
62	17.8	163	1	10.82	70		
63	17.7	11	1	0.68	69		
64	14.3	45	2	3.01	43		

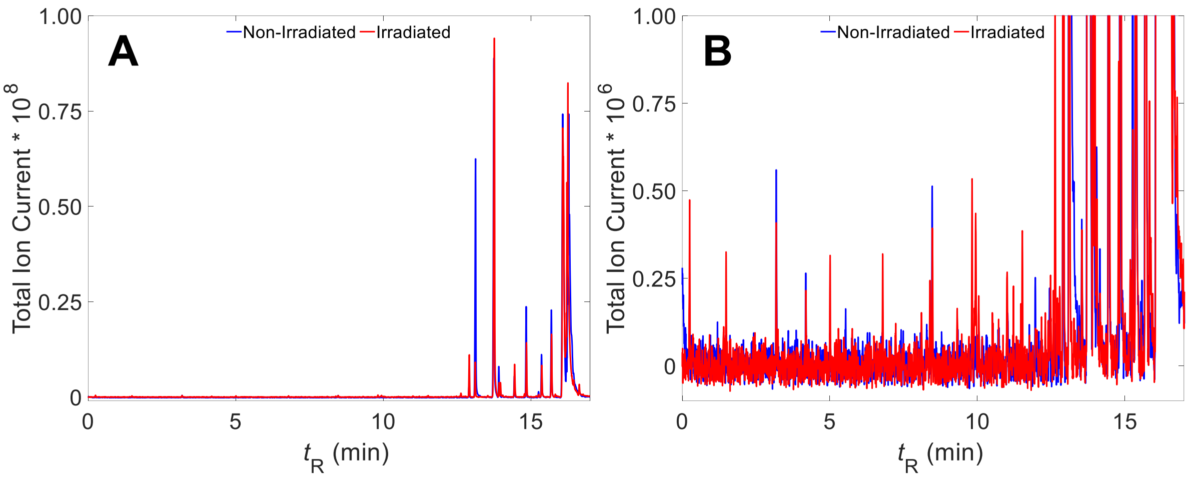

Figure S32: (**A**) GC-TOFMS TIC chromatograms for analysis of D-NO_2. (**B**) Zoom-in of A.

The TIC chromatograms for GC-TOFMS analysis of D-NO_2 can be found in Figure S31. While there are numerous impurities present from the synthesis, minimal chemical changes were observed in the TIC for D-NO_2.

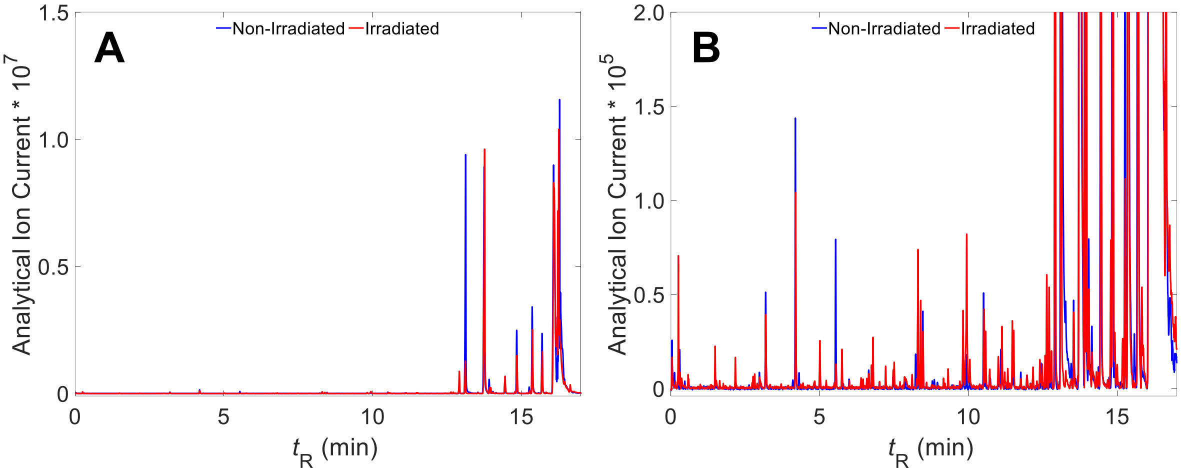

Figure S33: (**A**) GC-TOFMS AIC (m/z 61 and 43) chromatograms for analysis of D-NO_2. (**B**) Zoom-in of A.

The AIC chromatograms for GC-TOFMS analysis of D-NO_2 can be found in Figure S32. Numerous minor chemical changes were observed but only dodecane, dodecene, and nitrohexane were able to be identified as shown in Table S12.

Table S13: Fisher ratio results for the comparison of the irradiated and non-irradiated D-NO_2.

**Hit #**	**F-Ratio**	**Tile #**	**Grid #**	t_R **(min)**	**m/z with max F-ratio**	**Tentative Identification**	**Match Value**
1	300.5	197	1	13.13	61		
2	293.9	70	1	4.61	54		
3	197.6	76	1	5.02	68		
4	187.5	4	1	0.26	72		
5	164.1	165	2	11.01	55		
6	150.4	234	1	15.59	85		
7	144.6	166	1	11.04	123		
8	140.9	188	1	12.49	79		
9	137.3	190	1	12.64	124		
10	117.0	200	2	13.31	41		
11	115.8	201	1	13.34	55		
12	112.5	148	1	9.83	57	Dodecane	910
13	110.8	103	1	6.81	69		
14	106.0	122	1	8.11	68		
15	103.6	151	1	10.06	57		
16	95.5	199	1	13.21	110		
17	94.8	169	1	11.22	96		
18	94.4	238	1	15.84	109		
19	90.7	199	2	13.24	66		
20	88.2	96	2	6.40	82		
21	87.6	109	1	7.22	69		
22	87.2	201	2	13.37	56		
23	86.1	126	2	8.40	70		
24	85.9	200	1	13.27	56		
25	85.2	22	2	1.49	58		
26	81.4	239	1	15.89	109		
27	79.1	198	2	13.17	39		
28	77.4	140	2	9.32	55	Nitrohexane	849
29	67.0	208	1	13.81	99		
30	65.5	173	2	11.53	99		
31	63.8	233	2	15.55	115		
32	48.6	132	2	8.83	68		
33	47.8	202	1	13.41	56		
34	46.7	210	1	13.98	153		
35	44.5	149	2	9.95	69	Dodecene	944
36	43.2	45	2	2.98	67		
37	40.8	171	1	11.36	81		
38	40.2	206	2	13.74	94		

39	39.5	249	2	16.60	41		
40	38.7	21	2	1.41	56		
41	38.3	83	2	5.55	40		
42	37.1	180	1	11.99	89		
43	35.4	124	2	8.27	83		
44	33.8	167	2	11.17	95		
45	33.0	196	1	13.03	68		
46	30.6	204	1	13.56	135		
47	29.7	250	1	16.65	88		
48	29.3	146	1	9.72	54		
49	28.7	230	2	15.37	94		
50	28.6	209	1	13.93	91		
51	28.3	254	2	16.91	124		
52	27.5	191	1	12.67	82		
53	25.2	235	2	15.70	60		
54	24.5	246	1	16.36	259		
55	24.3	202	2	13.46	56		
56	24.2	222	2	14.80	59		
57	24.2	118	2	7.90	70		
58	23.7	170	2	11.33	58		
59	23.5	186	2	12.43	127		
60	23.4	184	1	12.21	83		
61	23.0	226	1	15.05	95		
62	22.6	195	2	12.98	83		
63	22.4	172	2	11.48	68		
64	22.2	228	1	15.19	81		
65	21.6	192	1	12.80	96		
66	21.0	204	2	13.59	56		
67	19.7	191	2	12.73	95		
68	19.2	205	2	13.68	79		
69	18.9	196	2	13.10	96		
70	18.6	229	2	15.29	87		
71	18.4	92	1	6.13	105		
72	18.0	156	2	10.39	68		
73	18.0	223	1	14.85	80		
74	17.9	216	1	14.38	60		
75	16.9	248	2	16.52	41		
76	16.6	63	2	4.19	58		
77	16.4	245	2	16.32	235		
78	16.1	161	1	10.70	82		
79	15.7	240	1	15.94	54		
80	15.5	184	2	12.26	141		

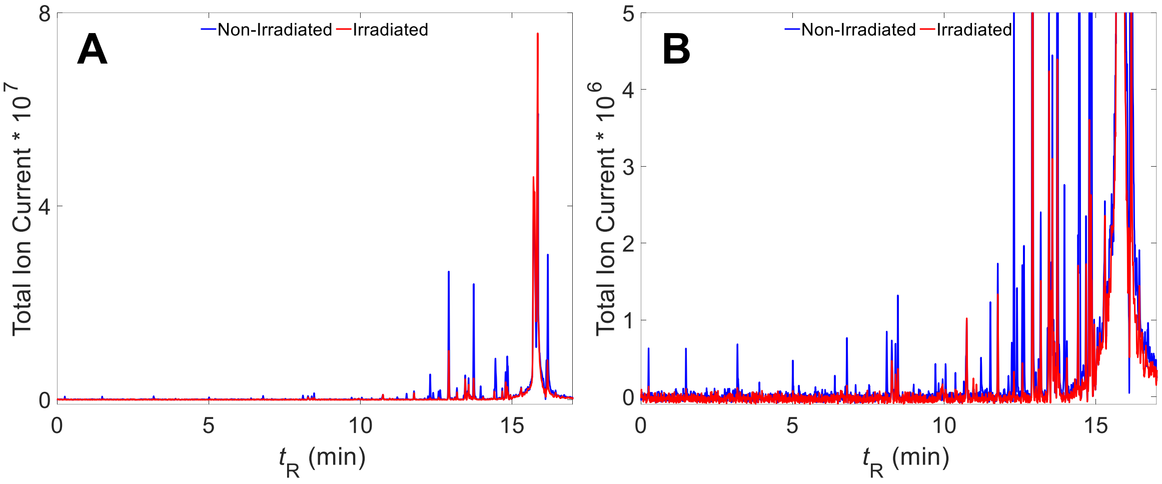

Figure S34: (**A**) GC-TOFMS TIC chromatograms for analysis of D-ONO_2. (**B**) Zoom-in of A.

The TIC chromatograms for GC-TOFMS analysis of D-ONO_2 can be found in Figure S33. While there are numerous impurities present from the synthesis, minimal chemical changes were observed in the TIC for D-ONO_2.

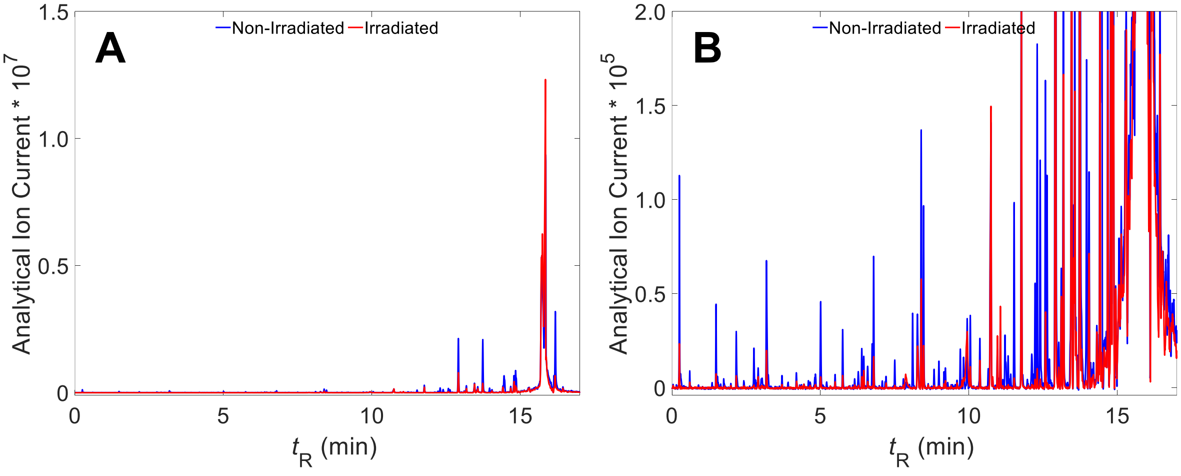

Figure S35: (**A**) GC-TOFMS AIC (m/z 43) chromatograms for analysis of D-NO_3. (**B**) Zoom-in of A.

The AIC chromatograms for GC-TOFMS analysis of D-ONO_2 can be found in Figure S34. Numerous minor chemical changes were observed but not all compounds could be identified, as shown in Table S13.

Table S14: Fisher ratio results for the comparison of the irradiated and non-irradiated D-ONO_2.

Hit #	F-Ratio	Tile #	Grid #	t_R (min)	m/z with max F-ratio	Tentative Identification	Match Value
1	833.3	210	1	13.97	111	Dodecanol	915
2	656.4	184	1	12.24	99		
3	565.5	185	1	12.31	99		
4	553.2	190	1	12.64	70	Undecanol	911
5	500.3	122	2	8.11	70	Octanitrate	741
6	457.7	182	2	12.12	76		
7	440.6	243	1	16.19	37		
8	434.2	200	2	13.33	83		
9	421.9	160	2	10.66	67	Heptanitrate	732
10	404.3	169	1	11.22	83		
11	380.9	173	2	11.53	70		
12	358.0	245	2	16.36	87		
13	354.9	201	2	13.42	97	Nonanitrate	807
14	353.1	217	2	14.46	62		
15	344.9	242	2	16.14	80		
16	342.5	186	2	12.41	126		
17	327.7	96	2	6.40	82		
18	327.0	193	2	12.90	97		
19	324.2	76	1	5.01	55		
20	319.4	180	1	11.98	69		
21	292.8	146	2	9.72	67		
22	266.1	49	1	3.21	85		
23	235.3	189	1	12.58	85		
24	232.7	192	1	12.76	83		
25	223.0	191	2	12.72	58		
26	222.8	214	1	14.26	67		
27	216.3	69	1	4.60	68		
28	214.9	171	1	11.36	81		
29	205.8	171	2	11.42	45	Undecanal	922
30	184.8	169	2	11.26	73		
31	168.1	181	2	12.06	76		
32	157.3	128	1	8.48	58		
33	151.2	103	1	6.81	67	Octanal	939
34	150.4	222	1	14.78	89		
35	146.6	206	2	13.75	36		
36	144.7	223	2	14.86	45		
37	138.3	167	2	11.15	71		
38	135.7	186	1	12.34	97		

39	131.2	188	1	12.51	127		
40	121.1	241	1	16.05	59		
41	114.6	232	2	15.46	76		
42	88.5	102	1	6.75	70		
43	80.3	195	1	12.95	87		
44	72.6	165	2	11.03	58		
45	66.5	151	2	10.06	55	Decanal	902
46	66.0	226	1	15.04	141		
47	61.1	198	1	13.17	126	Dodecanal	900
48	60.4	203	1	13.49	76		
49	60.0	208	1	13.84	113		
50	50.1	148	1	9.85	58		
51	49.3	199	2	13.28	87		
52	48.7	244	1	16.24	59		
53	46.6	219	1	14.55	100		
54	46.4	99	2	6.59	58		
55	39.9	228	2	15.20	59		
56	36.7	236	2	15.75	182		
57	30.2	219	2	14.59	100		

References

1 J. H. Hubbell and S. M. Seltzer, X-Ray Mass Attenuation Coefficients, *NIST Stand. Ref. Database 126*, , DOI:10.18434/T4D01F.

2 K. K. Gorai, A. Shastri, P. J. Singh and S. N. Jha, Experimental and theoretical studies on the absorption spectra of n-dodecane in the IR and VUV regions, *J. Quant. Spectrosc. Radiat. Transf.*, 2019, **236**, 106582.

3 E. Lieber, C. N. R. Rao, C. W. W. Hoffman and T. S. Chao, Infrared Spectra of Organic Azides, *Anal. Chem.*, 1957, **29**, 916–918.

4 M. S. Eroglu, B. Hazer and O. Güven, Synthesis and characterization of hydroxyl terminated poly(butadiene)-g-poly(glycidyl azide) copolymer as a new energetic propellant binder, *Polym. Bull.*, 1996, **36**, 695–701.

5 D. A. Dows, E. Whittle and G. C. Pimentel, Infrared spectrum of solid ammonium azide: A vibrational assignment, *J. Chem. Phys.*, 1955, **23**, 1475–1479.

6 B. D. Roos and T. B. Brill, Thermal decomposition of energetic materials 82. Correlations of gaseous products with the composition of aliphatic nitrate esters, *Combust. Flame*, 2002, **128**, 181–190.

7 C. Y. Panicker, H. T. Varghese and Y. S. Mary, FTIR, FT-Raman and DFT Calculations of 5-nitro-1,3-Benzodioxole, *Orient. J. Chem.*, 2012, **28**, 1037–1041.

8 H. Yamawaki, Raman spectroscopy of solid-phase n-dodecane and methyl oleate under high pressure, *Spectrochim. Acta - Part A Mol. Biomol. Spectrosc.*, 2020, **227**, 117756.

9 J. Šebek, L. Pele, E. O. Potma and R. Benny Gerber, Raman spectra of long chain hydrocarbons: Anharmonic calculations, experiment and implications for imaging of biomembranes, *Phys. Chem. Chem. Phys.*, 2011, **13**, 12724–12733.

10 G. C. Guo, Q. M. Wang and T. C. W. Mak, Structure refinement and Raman spectrum of silver azide, *J. Chem. Crystallogr.*, 1999, **29**, 561–564.

11 J. Wiss and A. Zilian, Online Spectroscopic Investigations (FTIR/Raman) of Industrial Reactions: Synthesis of Tributyltin Azide and Hydrogenation of Chloronitrobenzene, *Org. Process Res. Dev.*, 2003, **7**, 1059–1066.

12 S. Almaviva, S. Botti, L. Cantarini, A. Palucci, A. Puiu, A. Rufoloni, L. Landstrom and F. S. Romolo, Trace detection of explosives and their precursors by surface enhanced Raman spectroscopy, *Opt. Photonics Counterterrorism, Crime Fight. Def. VIII*, 2012, **8546**, 854602.

13 I. R. Lewis, N. W. Daniel and P. R. Griffiths, Interpretation of Raman spectra of nitro-containing explosive materials. Part I: Group frequency and structural class membership, *Appl. Spectrosc.*, 1997, **51**, 1854–1867.
